# Sequence‐Modulated Active Tripeptide Condensates for Tandem Catalysis

**DOI:** 10.1002/anie.202517620

**Published:** 2026-02-17

**Authors:** Hao Han, Siyu Song, Jianqiang Wang, Tsvetomir Ivanov, Dongdong Zhou, Hao Su, Katharina Landfester, Shoupeng Cao

**Affiliations:** ^1^ College of Polymer Science and Engineering National Key Laboratory of Advanced Polymer Materials Sichuan University Chengdu 610065 P. R. China; ^2^ Life‐Like Materials and Systems Department of Chemistry University of Mainz University of Mainz 55128 Mainz Germany; ^3^ Department of Physical Chemistry of Polymers Max Planck Institute For Polymer Research 55128 Mainz Germany

**Keywords:** active coacervates, cascade reaction, microreactor, short peptides, synthetic cells

## Abstract

Biomolecular condensates formed via liquid‐liquid phase separation function as dynamic organelles that are vital to regulating cellular activities. Peptide‐based coacervates have emerged as appealing candidates to resemble key properties of biomolecular condensates. However, their application as adaptive organelles has been hindered by structural complexity and limited control over phase‐separation. Here, we present short tripeptide coacervates with tunable phase‐separation behaviors governed by composition and peptide sequence, significantly reducing molecular complexity. These tripeptide condensates exhibit enzyme‐regulated phase‐separation, closely mimicking the dynamic nature of biomolecular condensates. A key attractive feature of the tripeptide coacervates is their capability to sequester both hydrophobic active species and hydrophilic enzymes. This unique property enables the execution of confined tandem reactions in aqueous conditions. When incorporated into membrane‐bound artificial cells, this tripeptide coacervates serve as adaptive sub‐organelles, orchestrating compartmentalized catalytic cascades. This work highlights the potential of minimalistic peptide systems as functional microreactors with biomimetic and catalytic capabilities.

## Graphical abstract

Sequence‐modulated tripeptide coacervates mimic biomolecular condensates with controllable, enzyme‐regulated phase separation. They co‐sequester hydrophobic active species and hydrophilic enzymes, enabling tandem catalysis in aqueous media. Incorporated into artificial cells, they function as adaptive sub‐organelles, orchestrating compartmentalized reactions, demonstrating minimalist peptide systems as tunable, functional microreactors with biomimetic and catalytic capabilities.

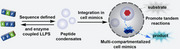

## 1 Introduction

Intracellular membraneless organelles, also known as biomolecular condensates, are ubiquitous in cells and play a critical role in regulating vital cellular processes and functions [1, 2, 3, 4, 5]. These condensates form reversibly through liquid‐liquid phase separation (LLPS) of proteins and nucleic acids, driven by multivalent interactions and dynamically regulated by enzymatic modifications [6, 7, 8, 9, 10]. By spatially concentrating active bio(macro)molecules in the confined liquid‐like environments, biomolecular condensates serve as functional compartments that modulate reaction rates and control reaction specificity [1, 11, 12, 13]. The spatial organization of biochemical pathways in biomolecular condensates enables complex networks of chemical transformations [14, 15, 16]. The importance of these natural dynamic structures has inspired the development of synthetic analogues that mimic their biophysical properties and catalytic functionalities [15]. Coacervate‐based systems formed through LLPS offer unique opportunities to emulate the crowding, dynamicity, and compartmentalization features in natural condensates [13, 16]. The coacervate droplets (usually 1–100 µm) provide a distinct microenvironment to sequestrate and concentrate a series of active species (e.g., enzymes and ribozymes) [17]. This enables coacervate droplets to act as a powerful and attractive platform to develop life‐like microreactors to perform complex catalytic pathways for various biotechnology purposes [18].

Protein‐derived (poly)peptides are biologically relevant building blocks in the formation of synthetic coacervate droplets [13, 19]. Peptide‐based coacervates are typically generated via complex coacervation between charged macromolecules—such as cationic polypeptides (e.g., poly‐L‐lysine, polyarginine) and polyanions (e.g., RNA, DNA, ATP)—or via self‐coacervation of intrinsically disordered peptides (e.g., elastin‐ or resilin‐like polypeptides) [19, 20, 21, 22]. Peptide materials exhibit attractive merits, such as the precise design of molecular building blocks, tailored sequences, and tunable structures [23, 24, 25, 26, 27, 28]. It provides a powerful means to systematically study the fundamental mechanisms underlying LLPS of peptides, revealing how composition, charge distribution, and sequence motifs govern the properties and functions of peptide coacervates. This led to an enhanced understanding of molecular grammar affecting LLPS and reducing the design complexity for condensate formation [18, 27, 29]. Notably, the development of short‐peptide (2‐10 amino acids) systems that retain coacervation ability while offering greater synthetic control has emerged [30, 31]. However, most short peptides tend to form rigid fiber‐like or gel‐like structures because the strong aromatic interactions and the extensive hydrogen bonding promote the alignment of peptide chains and ordered self‐assembly [32, 33, 34, 35]. Understanding the structural complexity and phase behavior at the molecular level remains a critical step toward expanding the utility of short peptide‐based coacervates as dynamic and functional biomimetic systems.

Short peptide‐based coacervates represent a minimalistic yet powerful platform for exploring the fundamental principles of biomolecular condensation [30, 36, 37, 38]. These systems are typically formed via self‐coacervation of a single peptide, driven by multivalent weak interactions such as hydrophobic, π–π, cation–π, and hydrogen bonding [23, 29, 38, 39]. Owing to their precisely defined molecular structures and precise controlled sequences, short peptide coacervates offer an unparalleled level of control, making them ideal scaffolds for studying the sequence–structure–function relationship and the underlying mechanisms governing phase separation [30, 40, 41]. Beyond their utility in fundamental research, short peptide coacervates also exhibit application potential through their customizable internal microenvironments, such as hydrophobicity, dynamics, and molecular crowding [27, 40]. Notably, they have been exploited as microreactors for chemical transformations, enabling organic reactions that typically involve poorly soluble substrates in aqueous media [25, 30, 32]. Despite these advances, the catalytic capabilities of short peptide coacervates remain underdeveloped, particularly in the context of complex tandem reactions where multiple, sequential steps must occur within a single, confined droplet. Emulating such functionality is critical, as it mirrors a key hallmark of natural organelles, the ability to integrate and coordinate multistep processes in a spatiotemporally controlled manner [41, 42]. Another important feature of living systems is multi‐compartmentalization, wherein distinct subdomains within a larger droplet enable hierarchical organization and functional segregation, akin to the internal architecture of eukaryotic cells [4, 5, 13, 43, 44]. Challenges in the compositional design and limited dynamics of peptide coacervates have hindered their utility as an active model of biomolecular condensates and limit their potential as functional modules to resemble biological hierarchical architecture and catalytic functions.

In this article, we present sequence‐defined liquid‐liquid phase separation of tripeptides to engineer active condensates that promote complex tandem catalysis. By precisely controlling the molecular composition and sequence, liquid coacervate droplets with minimal tripeptides can be obtained in a highly selective and controlled manner. The tripeptide condensates exhibited reversible and dynamic phase separation regulated by enzymatic reactions, resembling the adaptive formation of intracellular biomolecular condensates. The tripeptide condensates exhibited selective sequestration and partitioning capabilities for both hydrophobic and hydrophilic catalytic species. This dual compatibility enables the execution of complex tandem catalytic reactions—including sequential organic and enzymatic steps—within a single aqueous‐phase droplet. Acting as biomimetic microreactors, the tripeptide condensates achieve spatial confinement, catalytic co‐localization, and enhanced reaction efficiency, which are critical features of natural organelles but rarely demonstrated in such minimal synthetic systems. To further mimic cellular organization, we integrated tripeptide condensates into a model membrane‐bound artificial cell, where they function as dynamic sub‐organelles. These peptide‐based sub‐compartments not only retained their adaptive features but also enabled complex tandem catalytic reactions and the generation of functional outputs—such as fluorescent reporters—within a multi‐compartmentalized, cell‐like environment. Our results advance the frontiers of synthetic biomolecular condensates and open new avenues for engineering minimal, tunable, and functional materials for systems chemistry, synthetic biology, and the bottom‐up construction of protocells.

## 2 Result and Discussion

Among the peptide building blocks for engineering bioinspired systems, phenylalanine dipeptides (FF) were widely utilized to construct phase‐separation materials [45, 46]. However, short peptide coacervates with FF moieties usually exhibited a strong tendency to transform into thermodynamically more stable fibrous structures during the aging process. This conversion can be ascribed to the strong aromatic, hydrophobic interactions and hydrogen bonding guidance facilitated the dehydration of the coacervates, promoting a nucleation process and formation of fibrous structures [32, 47]. Recent studies have shown that the liquid state of FF coacervates can be maintained via the incorporation of bulky capping groups or mixing of binary peptides [29, 48]. The enhanced meta‐stability of peptide coacervates is likely due to the prevention of ordered crystalline domain formation via manipulating the intra/inter‐molecular interactions. In this regard, we hypothesized that alternation of aromatic interaction of peptides via inserting an amino acid in a different position of FF peptides would be a viable strategy to modulate their phase separating behaviors. Our primary objective was to establish minimal tripeptide condensates as adaptive biomimicry systems and explore their functional utility rather than to exhaustively map the physical phase space of the system.

**SCHEME 1:**
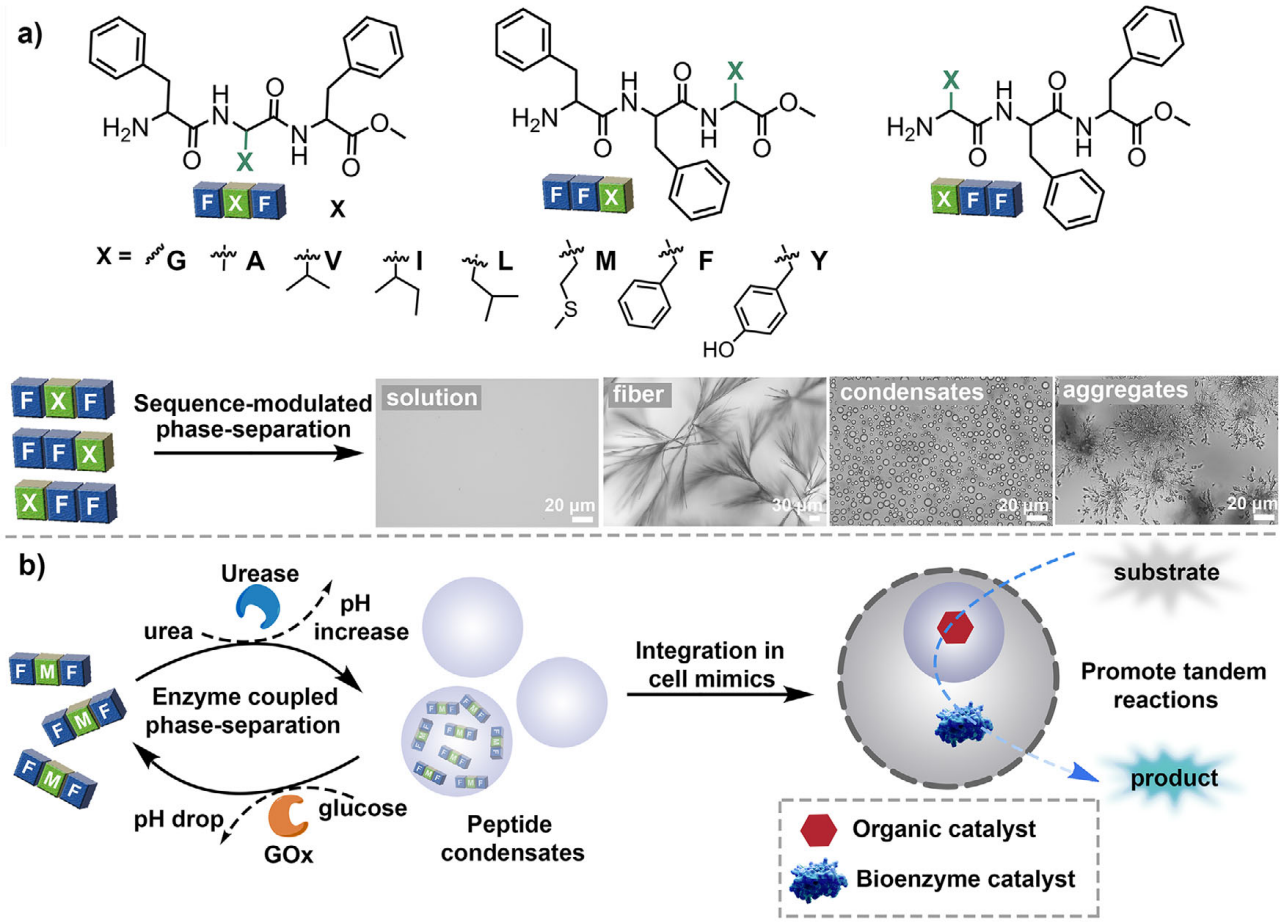
Design and assembly of active controlled short peptide‐based coacervates. (a) Tripeptides with tailored composition and sequence used in controlling their phase separation behaviors. (b) Schematic illustration of enzymatic‐reaction‐controlled phase‐separation behaviors of tripeptides, which allow them to be integrated as sub‐organelles in a model synthetic cell. This further enables an organic‐enzymatic tandem reaction to occur in the compartmentalized cell mimics.

A series of tripeptides FFX, FXF, XFF (where X = G, A, V, I, L, M, F, Y) were selected and prepared to investigate the effect of peptide composition and sequence in LLPS (Scheme 1 and Schemes S1–S3, Figures S1–S24, and Table S2). The presence of an amino group enables tripeptides to exhibit pH responsiveness. All peptides were dissolved as a transparent solution in 5 mM Hepes buffer at pH ∼6, but underwent significant phase‐separation behaviors at a higher pH by adding a few drops of 0.1 M NaOH solution (Table S2 and Figure S25). When X amino acids were short‐chain aliphatic hydrocarbons such as G and A, the tripeptides displayed sequence‐modulated and concentration‐dependent phase separation behaviors. For instance, the tripeptides (GFF, FGF, FFG, and AFF, FAF, FFA) still remained solubilized below 5 mg mL^−1^ at pH ∼8, but assembled at a higher concentration (≥ 10 mg mL^−1^) (Figures S26–S31). All phase behaviors described were reproducibly observed across independently prepared samples. Particularly, the tripeptides FGF, FFG, FAF, and FFA (20 mg mL^−1^) assembled into coacervate droplets at pH ∼8 and remained stable during 30 min observation (Figures S27 and S28, S30 and S31). However, GFF and AFF (20 mg mL^−1^) formed aggregate structures at pH ∼8 (Figures S26 and S29). The sequence‐modulated phase‐separation behaviors can be ascribed to different chain flexibility and hydration level of capping groups, as well as alteration of aromatic interactions. The insertion of a G or A amino acid between phenylalanine groups (e.g., FGF and FAF) may enhance the flexibility of the backbone and fluidity of condensates, thus preventing the aging process [23, 49, 50].

**FIGURE 1:**
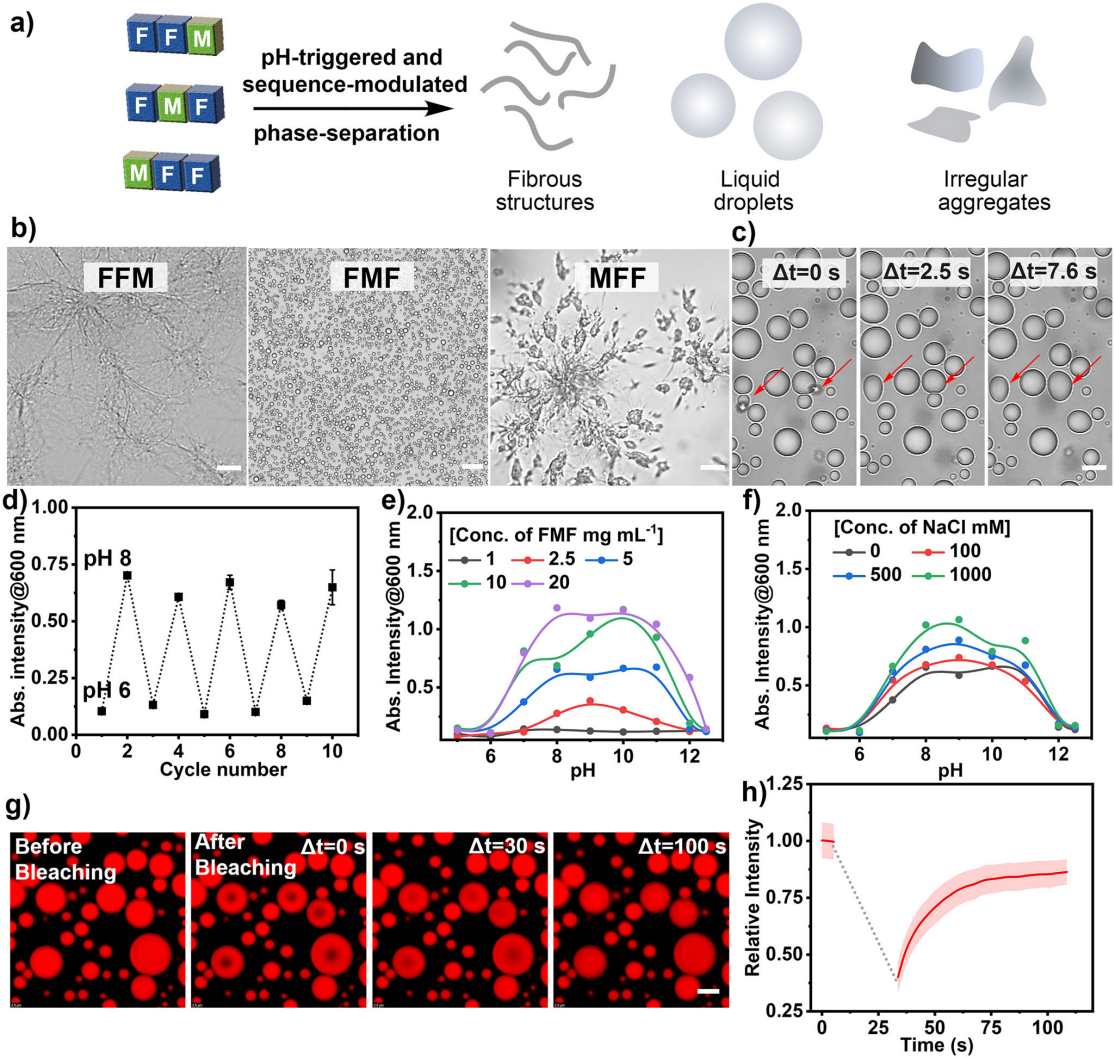
Sequence‐modulated tripeptide coacervates and key properties of coacervates: (a) Schematic illustration of liquid‐liquid phase separation in tripeptide coacervates, depending on modulation of peptide sequence. (b) Microscope images of FFM, FMF, and MFF assembled at pH ∼8 (in 5 mM Hepes buffer) post 30 min observation. Scale bar = 10 µm, 30 µm, and 10 µm, respectively. Three experiments were repeated independently with similar results. (c) The FMF coacervates are liquid, as evidenced by their coalescence into larger droplets. Scale bar = 5 µm. Three experiments were repeated independently with similar results. (d) Turbidity changes of the FMF peptide solution (5 mg mL^−1^dissolved in 5 mM Hepes buffer) at pH ∼6 and pH ∼8, indicating reversible phase‐separation behaviors of FMF at different pH. Data represent mean ± SD for n  =  3 independent samples. Error bars depict the standard deviation (SD) obtained from microplate reader analysis. (e) Turbidity changes of FMF coacervates under different pH and concentration conditions. (f) Turbidity changes of FMF condensates (5 mg mL^−1^dissolved in 5 mM Hepes buffer) under different pH and NaCl conditions. (g, h) Confocal images corresponding to FRAP and FRAP traces of FMF coacervates (5 mg mL^−1^dissolved in 5 mM Hepes buffer, 100 mM NaCl) over time. Data represent mean ± SD for n  =  5 representative microscopic images. Error bars (red shaded area) depict the standard deviation (SD) from confocal imaging analysis. Scale bar = 5 µm.

Modification of G and A in the right position of phenylalanine dipeptides (e.g., FFG and FFA) possibly enhanced the hydration level of the hydrophobic FF domain, thereby enhancing the metastability of the coacervates [29]. When the X amino acids were medium or long aliphatic chain (V, I, L), the position of X amino acids also induced a significant effect on the phase‐separation behaviors. The assembly of VFF, IFF, and LFF (5 mg mL^−1^) at base conditions resulted in the formation of typical aggregates (Figure S32, S36, and S40) in 5 min incubation at pH∼8. However, the tripeptides FFV, FFI, and FFL formed liquid coacervates and were stable during 30 min observation (Figures S34 and S35, S38 and S39, and S42). The stable coacervate droplets can benefit from bulky capping groups that suppress the formation of supramolecular nanofibers via steric hindrance [51]. Interestingly, FVF and FIF initially formed coacervate droplets at pH ∼8 but quickly converted into fiber‐like structures (less than 5 min) (Figure S33 and S37). But the assembly of FLF resulted in coacervate droplets, which were stable during 30 min incubation (Figure S41). The different observations possibly can be the balancing results of aromatic and hydrophobic interactions, where disrupting the FF π‐π interactions inhibits the transition into solid‐type aggregates while enhanced hydrophobic interactions promote formation of an ordered fiber structure [52]. In addition, V and I amino acids as side chain groups were recognized to promote fiber formation due to their strong tendency for *β*‐sheet formation [53]. When the X amino acid was a polar group like methionine, MFF and FFM initially formed coacervate droplets at pH ∼8 but quickly transformed into fiber structure or irregular aggregates, respectively (Figure 1b and Figures S43 and S44) [48]. The phase‐separation of FMF resulted in stable droplets, which can possibly be ascribed to the decreased aromatic interactions that maintain the disordered assembly state (Figures S45 and S46). When X was aromatic structures like F and Y, the tripeptide structure (FFF, FYF, FFY) initially formed droplets that rapidly solidified and formed fiber‐like or irregular aggregates under alkaline conditions, possibly due to the enhanced aromatic interactions that induced ordered molecular packing (Figures S47–S49) [54]. Interestingly, although the assembly of YFF formed spherical morphology like coacervate droplets, they showed diminished fluorescent recovery after photobleaching, suggesting their internally rigid nature (Figures S50 and S51). These results indicate that the molecular composition and sequence would be of great importance in guiding their assembly and phase separation behaviors. In the above cases, adjusting the aromatic interactions, altering hydrophobic interactions, and modification of capping groups would be considered as critical factors that altered the phase‐separation behaviors of short peptides.

After investigation of the general phase‐separation behaviors of the tripeptides, we select FMF to further investigate the properties of tripeptide coacervates, including their dynamic formation and life‐like behaviors. FMF coacervates showed fusion, coalescence, and growth behaviors, and were found to contain a substantial amount of water (80.4 ± 6.4%), indicating liquid‐like features (Figure 1c). The formation of tripeptide FMF coacervates was highly pH‐reversible and could be repeated over several cycles between pH ∼6 and ∼8 due to the altered hydration level of the amino group in the peptide (Figure 1d and Figures S52 and S53). The minimal concentration for the formation of coacervate droplets is ∼ 2.5 mg mL^−1^ (Figure 1e and Figure S46). Conventional complex coacervates formed via electrostatic interactions are usually affected by the presence of a higher concentration of salt; however, FMF (5 mg mL^−1^) coacervates displayed robust resistance to salt, where the turbidity of FMF coacervates even increased as the NaCl concentration increased from 0 to 1000 mM, possibly due to the enhanced hydrophobic interactions (Figure 1f and Figure S54) [55]. The FMF coacervate solution displayed decreased turbidity with prolonged incubation time due to coalescence or sediment on the surface, while their liquid droplet state after sediment on the surface of glass was found to remain relatively stable over ∼1 week observation (Figures S55 and S56, the capture of optical images were illustrated in Supporting Information Section 4.1). To further investigate the liquid feature of FMF coacervates, fluorescence recovery after photobleaching (FRAP) (Figure 1g,h) was utilized [56]. After photobleaching, FMF coacervates were found to recover to ∼80% fluorescence intensity of the initial level, indicating their inherent liquid‐like properties. In contrast, MFF assemblies barely showed fluorescence recovery after photobleaching, displaying rigid properties (Figure S57). To investigate the role of hydrophobic interaction in driving the phase‐separation of FMF peptides, 1,6‐hexanediol, a compound known to disrupt weak hydrophobic interactions, was added to the solution of FMF coacervate (5 mg mL^−1^, 10.9 mM, pH ∼7) [55]. The presence of 1,6‐hexanediol (HDO) significantly decreased the solution turbidity of FMF coacervate solution (at *λ_abs_* = 600 nm). Microscopic images indicated that FMF coacervates almost all disassembled upon addition of 1207 mM 1,6‐hexanediol (Figure S58). This indicated that hydrophobic interactions play a significant role in driving the droplet formation. The hydrogen bonding effect in the phase‐separation of FMF peptides was then investigated by treating FMF coacervates with urea, a compound known to disrupt non‐covalent polar interactions in aqueous solution [57]. The addition of urea induced a sharp decrease in the turbidity of FMF coacervate solution, and microscopic images revealed a significant decrease in the peptide droplets when treated with 3 M urea. An increased concentration of urea (5 M) led to the total disassembly of the FMF coacervates (5 mg mL^−1^, 10.9 mM, pH ∼7) (Figure S59). These observations indicated that hydrogen bonding plays an important role in driving the liquid‐liquid phase separation of FMF peptides. The presence of the thioether group rendered the FMF coacervates oxidation‐responsive and allowed the dynamic dissolution by oxidation chemistry under mild conditions [9]. Treating the FMF coacervate solution with 1% H_2_O_2_ (∼332 mM) induced dissolution of the droplets within 10 min, due to the oxidation of the thioether group into a more polar sulfoxide or sulfone format that enhanced water solubility of the peptides, confirmed by HPLC analysis (Figures S60 and S61).

**FIGURE 2:**
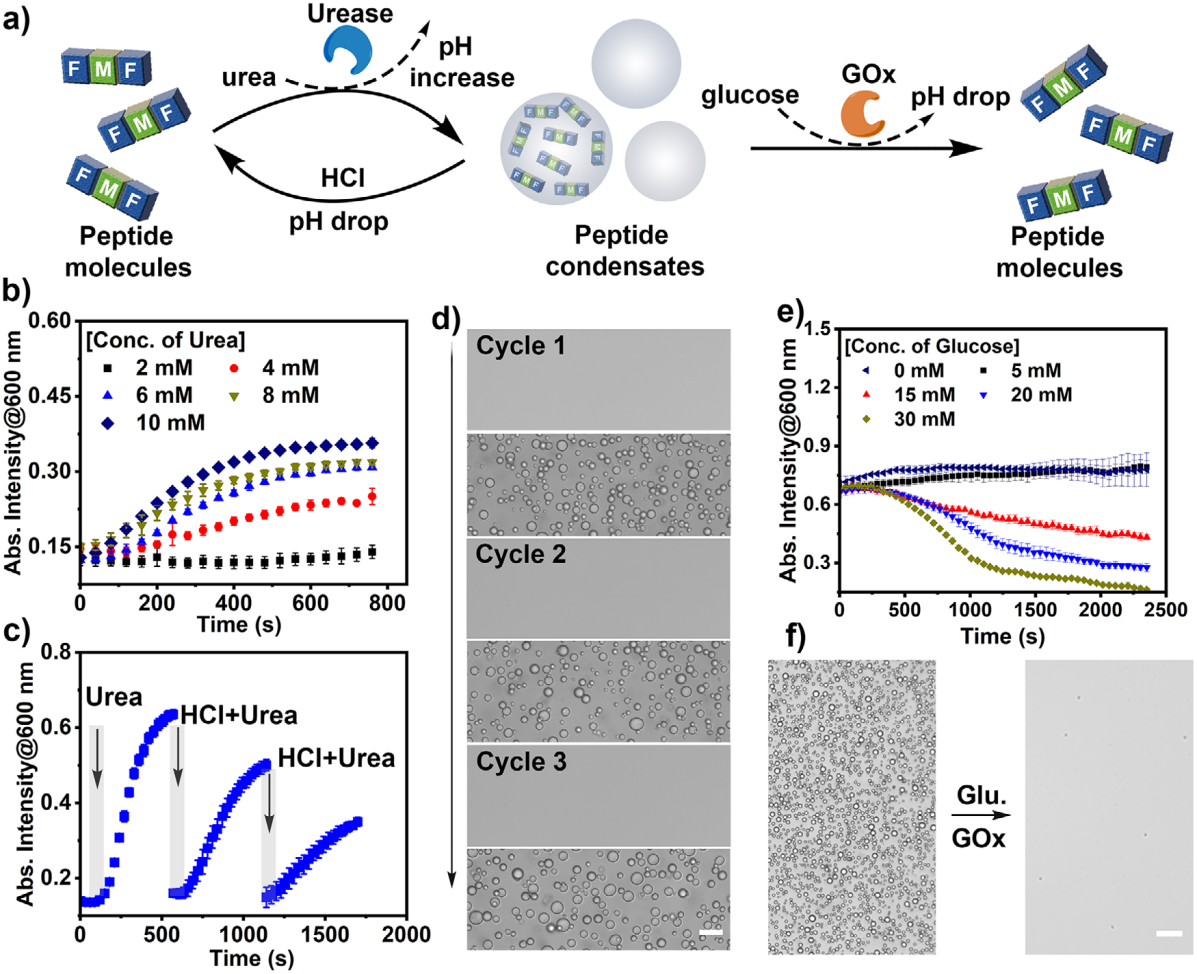
Enzymatic‐regulated dynamic phase‐separation of FMF peptide. (a) Schematic illustration of reversible liquid‐liquid phase separation of FMF coacervates controlled by enzymatic reactions. (b) Turbidity changes of FMF peptide solution (10.9 mM FMF peptide, 0.02 g L^−1^ urease) incubated with different concentrations of urea. Data represent mean  ±  SD for n  =  3 independent samples. Error bars depict the standard deviation (SD) obtained from microplate reader analysis. (c) Reversible generation and disassembly of FMF coacervates controlled by sequentially adding urease/urea and hydrochloric acid. Scale bar = 10 µm. Data represent mean  ±  SD for n  =  3 independent samples. Error bars depict the standard deviation (SD) obtained from microplate reader analysis. (d) Microscopy images of FMF peptides were obtained by sequentially adding urease/urea and hydrochloric acid. (e) Turbidity changes of FMF coacervates (10.9 mM FMF peptide, 0.2 g L^−1^ GOx) at different concentrations of glucose. Data represent mean  ±  SD for n  =  3 independent samples. Error bars depict the standard deviation (SD) obtained from microplate reader analysis. (f) Microscopy images of FMF peptides treated with 30 mM glucose (Glu.) after 40 min. Scale bar = 30 µm.

One of the key features of biomolecular condensates is their dynamic regulation by enzymatic reactions [58]. This enzymatic control allows condensates to reversibly assemble and disassemble in response to biochemical signals, thereby regulating compartmentalization, reaction rates, and molecular exchange within cells [59]. Considering FMF coacervates displayed superior pH‐responsive formation and dissolution (Figure 1d), we then investigated whether the dynamic formation of FMF coacervates can be coupled and regulated by enzymatic reactions. To this end, the use of glucose oxidase (GOx) and urease, commonly as pH‐regulators, was utilized in the dynamic formation of FMF coacervates [60]. Glucose oxidase reduces the solution pH value by converting glucose into gluconic acid, while urea increases the solution pH value by hydrolysis of urea into ammonia. Enzyme‐triggered phase transitions were characterized by continuous turbidity tracking, allowing quantification of formation and dissolution kinetics. The FMF peptide solution (5 mg mL^−1^, 10.9 mM, pH ∼6) was first treated with urease (∼ 0.02 g L^−1^) and a varied concentration of urea (2 mM to 10 mM). With a concentration of urea higher than 4 mM, the turbidity of FMF peptide solution (at *λ_abs_* = 600 nm) increased significantly after ∼ 600 s incubation, indicating assembly and phase‐separation of FMF peptide upon urease‐mediated pH elevation (Figure 2b and Figure S62). The urease‐mediated regulation of FMF coacervation was highly reversible via sequentially adding urea and HCl, as revealed by turbidity change and microscopic imaging (Figure 2c,d and Figure S63). After that, the dynamic dissolution of FMF coacervates was then investigated by the addition of GOx and glucose (Figure 2e). As GOx oxidizes glucose to gluconic acid (lowering pH) while generating H_2_O_2_, dissolution of FMF coacervates could be the result of either acidification‐induced disassembly or H_2_O_2_‐mediated oxidation of the methionine (M) thioether bond, both increasing the solvation level of FMF peptides. FMF coacervate solution (5 mg mL^−1^, 10.9 mM, pH ∼8.0) was then treated with GOx (0.2 g L^−1^) and different concentrations of glucose. The addition of glucose (5 mM to 30 mM) induced dynamic control of the dissolution kinetics of FMF coacervates. For instance, when fed with above 30 mM glucose, the turbidity of the FMF coacervate solution gradually decreased during ∼40 min incubation. This indicated the dissolution of FMF coacervates by GOx‐mediated reaction, confirmed by microscopy imaging (Figure 2f). Triplicate measurements confirmed consistent concentration‐dependent LLPS and enzyme‐triggered transitions, underscoring the robustness of the sequence‐defined condensates. Further HPLC analysis and pH titration experiment demonstrated that the GOx‐mediated dissolution of peptide coacervates is primarily due to the solution pH decrease, rather than the oxidation‐mediated pathway (Figure S64).

**FIGURE 3:**
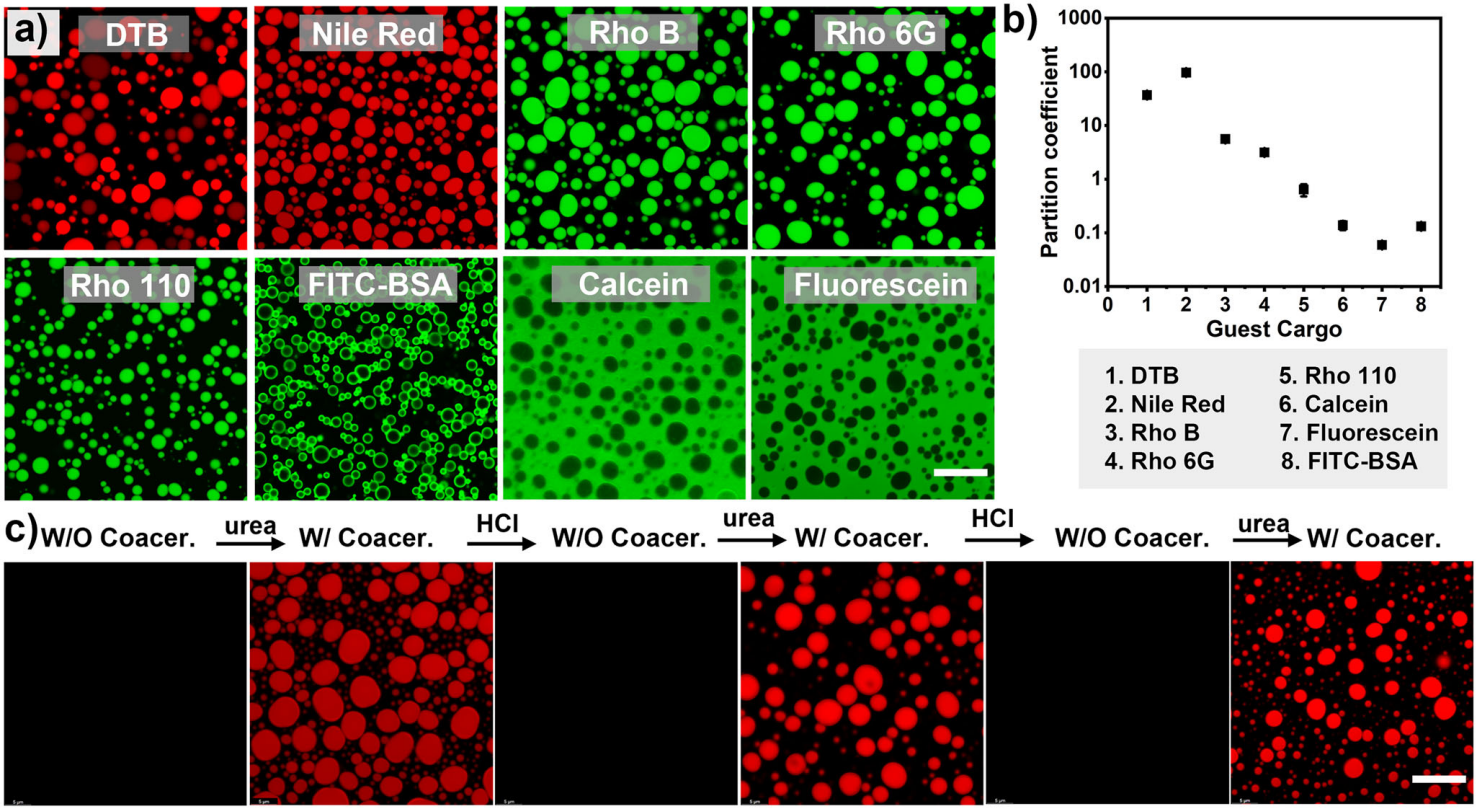
Dynamic sequestration of guest molecules in FMF coacervates. (a) Confocal images of FMF coacervates (5 mg mL^−1^, 10.9 mM) after incubation with different guest molecules (dye concentrate: 0.005 mg mL^−1^). Scale bar = 20 µm. Three experiments were repeated independently with similar results. (b) Partitioning coefficients of different guest molecules in FMF coacervates were determined by fluorescent intensity measurement after centrifugation, by comparing dye content in the concentrated coacervate phase and in the surrounding diluted phase. Data represent mean  ±  SD for n  =  3 representative fluorescent measurements. Error bars depict the standard deviation (SD) from fluorescent intensity analysis. (c) Reversible sequestration of guest molecules (Nile Red, 0.005 mg mL^−1^) in FMF coacervates by sequentially adding urease/urea and HCl, revealed by confocal imaging. Scale bar = 20 µm.

Coacervate droplets serve as an ideal model of natural compartments in terms of molecular crowding and partitioning of active biomolecules, which allows them to regulate the efficiency and selectivity of biochemical reactions. We then investigate the potential of FMF peptide coacervates for the sequestration of guest species. The ability of FMF coacervates to concentrate small molecules and biomacromolecules was then evaluated using confocal microscopy. Aromatic fluorophores such as DTB (4,7‐di(2‐thienyl)−2,1,3‐benzothiadiazole, a hydrophobic photocatalyst, Scheme S4) and Nile Red were concentrated in FMF coacervates, with partition coefficients (K = content_droplet phase_/content_diluted phase_) of ∼36 and ∼96, respectively, which were extracted from fluorescence intensities in dilute and dense phases to quantify molecular sequestration. Positively charged dyes such as rhodamine B and rhodamine 6G were also enriched inside FMF coacervates, with partition coefficients of 5.5 and 3.1, respectively. Zwitterion dye Rhodamine 110 and negatively charged dyes, including fluorescein and calcein, showed decreased concentrating effect, with partition coefficients of ∼0.60, ∼0.05, and ∼0.13, respectively (Figure 3a,b and Figures S65 and S66). The above results indicate that FMF coacervates displayed a higher propensity to concentrate aromatic and cationic species, which is probably facilitated by π ‐π and cation‐π interactions between the guest cargoes and the phenylalanine residues within the droplets [61]. When comparing the fluorescent intensity of a typical hydrophobic cargo (e.g., Nile red) in organic solution (e.g., acetonitrile), the fluorescent intensity of Nile red in FMF coacervates is ∼70% of that in acetonitrile, but ∼300 times that in aqueous solution (Figure S67). This indicates the internal microenvironment of FMF is more similar to an organic solvent than an aqueous environment. Interestingly, biomacromolecules such as FITC‐BSA can also be confined within the FMF coacervates, with a partition efficiency of ∼0.13 (Figure 3a). This indicates that FMF coacervates display potential to serve as versatile compartments to concentrate various active species. In addition, the dynamic formation and dissolution of FMF coacervates, regulated by enzymatic reactions, enabled the active and reversible concentration of guest cargoes. This was confirmed by reversible sequestration of Nile red inside FMF coacervates upon sequential addition of urea and HCL to a FMF solution containing urease (Figure 3c).

**FIGURE 4:**
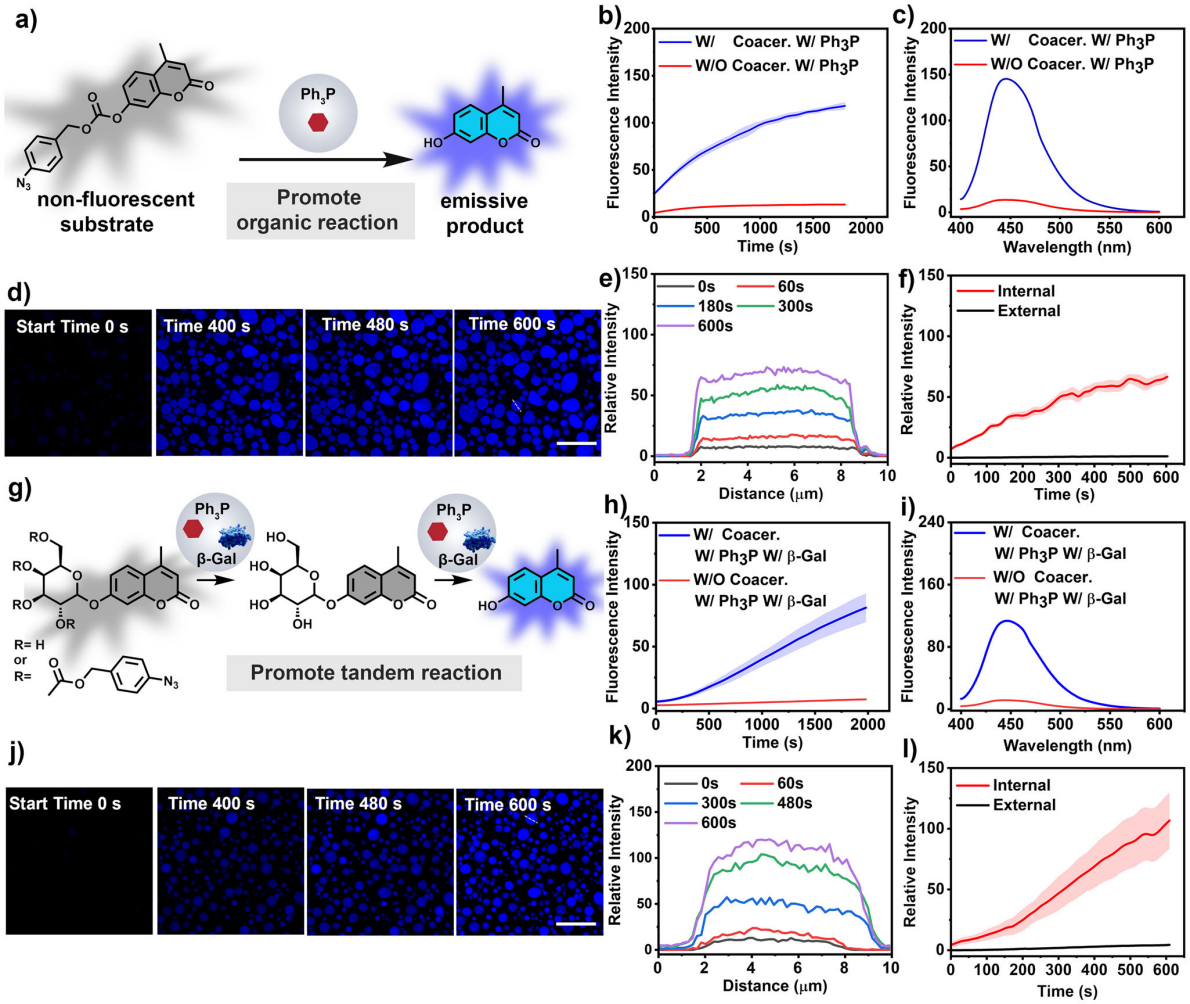
FMF coacervates as a microreactor for cascade catalysis. (a) Schematic illustration of a Staudinger Reaction occurring in FMF coacervates. The reaction results in the generation of a highly emissive fluorophore. (b) Fluorescence emission from the Staudinger reaction. In the presence of Ph_3_P‐containing microreactors (10.9 mM peptides, pH ∼7, 100 mM NaCl, 0.228 mM Ph_3_P, 0.285 mM N_3_‐coumarin), a significant increase of fluorescence emission (λ*_em_*  =  450 nm) was observed. In contrast, barely increase in emission was observed in the absence of Ph_3_P microreactors (the same condition without coacervates). Data represent mean  ±  SD for n  =  3 independent samples; Error bars (blue shaded area) represent the standard deviation (n  =  3) from microplate reader analysis. (c) Corresponding fluorescence emission spectra of the reaction after 30 min incubation under different conditions. (d) Confocal images showed a gradual increase in fluorescence intensity within the coacervates (10.9 mM peptides, pH ∼7, 100 mM NaCl, 0.19 mM Ph_3_P, 0.57 mM N_3_‐coumarin), scale bar = 25 µm. Blue colour indicated the generation of coumarin derivatives; (e) Fluorescence intensity profile of coumarin across microreactors shown in (d); (f) Comparison of the fluorescence intensity inside and outside the microreactors shown in (d), data represent mean  ±  SD for n  =  5 representative microscopic images of view. (g) Schematic representation of the tandem reaction in FMF coacervates. (h) Comparison of fluorescence intensity in the presence or absence of coacervates (0.228 mM Ph_3_P, 3 U mL^−1^ *β*‐Gal, 0.29 mM N_3_‐g‐coumarin). There is a significant increase in fluorescence emission (λ*_em_*  =  450 nm) when coacervate was present (10.9 mM FMF peptide, pH∼7, 100 mM NaCl). Data represent mean  ±  SD for n  =  3 independent samples; Error bars (blue shaded area) represent the standard deviation (n  =  3) from microplate reader analysis. (i) Corresponding fluorescence emission spectra of the tandem reaction after 30 min incubation under different conditions. (j) Confocal images show a gradual increase in fluorescence intensity within the coacervates (10.9 mM peptides, pH ∼7, 100 mM NaCl, 0.19 mM Ph_3_P, 2.5 U mL^−1^ *β*‐Gal, 0.29 mM N_3_‐g‐coumarin). Scale bar = 25 µm. Blue colour indicated the generation of coumarin derivatives; (k) Fluorescence intensity profile of coumarin across microreactors shown in (j); (l) Comparison of the fluorescence intensity inside and outside the microreactors shown in (j), data represent mean  ±  SD for n  =  5 representative microscopic images of view.

Having confirmed the ability to enrich both hydrophobic and hydrophilic species within FMF coacervates, their potential as a biomimetic microreactor to perform biochemical reactions and synthesize active compounds was further explored. Integration of active species inside coacervate droplets provides a valuable toolbox to design life‐like systems with application in bio‐inspired catalysts [48, 62]. We then explored the utility of FMF coacervates as a reaction centre to perform localized and tandem catalytic reactions in aqueous media. To demonstrate the utility of organic reactions in coacervate systems, we selected the Staudinger reduction as a model reaction. In this transformation, an azide compound reacts with triphenylphosphine (Ph_3_P) to form a transient iminophosphorane intermediate, which subsequently undergoes hydrolysis to yield the corresponding amine along with triphenylphosphine oxide (Ph_3_P = O) as a byproduct [63, 64, 65, 66]. This reaction proceeds under mild conditions and is highly chemoselective, making it an ideal candidate for integration into compartmentalized systems in the aqueous phase. Hydrophobic Ph_3_P and para‐azidobenzyl carbonate‐4‐methylcoumarin (N_3_‐coumarin, Scheme S5 and S6) were used as the active species in the Staudinger reduction, which were found to be effectively concentrated inside coacervates (the loaded efficiency of Ph_3_P and N_3_‐coumarin inside coacervates was calculated to be ∼ 98% and 97% respectively, Figures S68 and S69). FMF peptide coacervates (10.9 mM, pH ∼7, 100 mM NaCl) were then treated with Ph_3_P (76 mM in DMSO, final concentration 0.228 mM) and N_3_‐coumarin (57 mM in DMSO, final concentration 0.285 mM), which did not impact the inherent liquid state, stability and dynamic properties of peptide coacervates (Figures S70 and S71). An increased fluorescent intensity (λ*_em_* = 450 nm) was observed during ∼1500 s incubation, monitored by a microplate reader, due to the reduction of the azide group and the hydrolysis of the carbonate, which releases hydroxymethyl coumarin that showed strong blue fluorescence (Figure 4a–c and Figure S72). However, in the absence of FMF coacervates, there was barely fluorescence increase during the observation. The fluorescent intensity of the emissive product in the presence of FMF coacervates was ∼33 times that without FMF coacervates. The production of emissive coumarin with FMF coacervates was further confirmed by confocal microscopy imaging, in which the emission was mainly confined inside FMF coacervates (Figure 4d–f). The increased fluorescent intensity with FMF coacervates can be ascribed to the enhanced solvation of the hydrophobic active species, as well as the concentrating effect by compartmentalization that boosted the reaction efficiency in aqueous media [61].

Biomolecular condensates are pivotal in coordinating complex metabolic networks such as enzymatic cascades, clusters of interconnected metabolic pathways with multiple steps, or tandem reactions involving metal complexes and biological enzymes in molecularly crowded environments [62, 67]. We then investigate the potential utility of FMF coacervates to perform complex reactions, such as organic active species and biocatalyst‐mediated tandem reactions. In this regard, we employed small triphenylphosphine molecules as an active species and *β*‐galactosidase (*β*‐Gal) as a hydrophilic enzymatic catalyst to facilitate organic‐enzymatic cascades within coacervate microreactors. The design is based on the hydrolysis of the carbonate after azide reduction, exposing the galactose group, which was then hydrolysed by the enzyme, yielding a fluorescent product (Figure 4g). An azido‐masked galactose‐coumarin derivative (N_3_‐g‐coumarin, Scheme S7) was prepared as previously reported and used as the substrate [68]. The substate N_3_‐g‐coumarin, compared with the intermediate g‐coumarin, displayed preferred partitioning inside the peptide coacervates, which also displayed a concentrating effect toward *β*‐Gal (the loaded efficiency of β‐Gal inside coacervates was calculated to be ∼ 18.7%, Figures S73–S75). Then, to a 5 mg mL^−1^ FMF peptide (10.9 mM) coacervate solution, Ph_3_P (DMSO solution, final concentration 0.228 mM) and *β*‐Gal (Tris buffer solution, final concentration 3 U mL^−1^) were added and followed by the introduction of N_3_‐g‐coumarin (DMSO solution, final concentration 0.29 mM). The change of fluorescent intensity was monitored (*λ_em_* = 450 nm) by a microplate reader. In the presence of FMF coacervates, a gradual increase in fluorescence was observed during 30 min incubation, which was significantly ∼11 times higher than that without FMF coacervate droplets (Figure 4h,i and Figure S76). Confocal laser scanning microscopy (CLSM) further revealed a progressive rise in fluorescence values inside the peptide microreactor (Figure 4j–l). The above results revealed the successful establishment of tandem reaction pathway inside the coacervate microreactor, which hold the potential to build more complex metabolic networks, closer mimicking the function of biomolecular condensates. The value of this platform lies in enabling confined, multi‐step chemistry inside minimal synthetic organelles, rather than in the preparative synthesis of chemical products.

**FIGURE 5:**
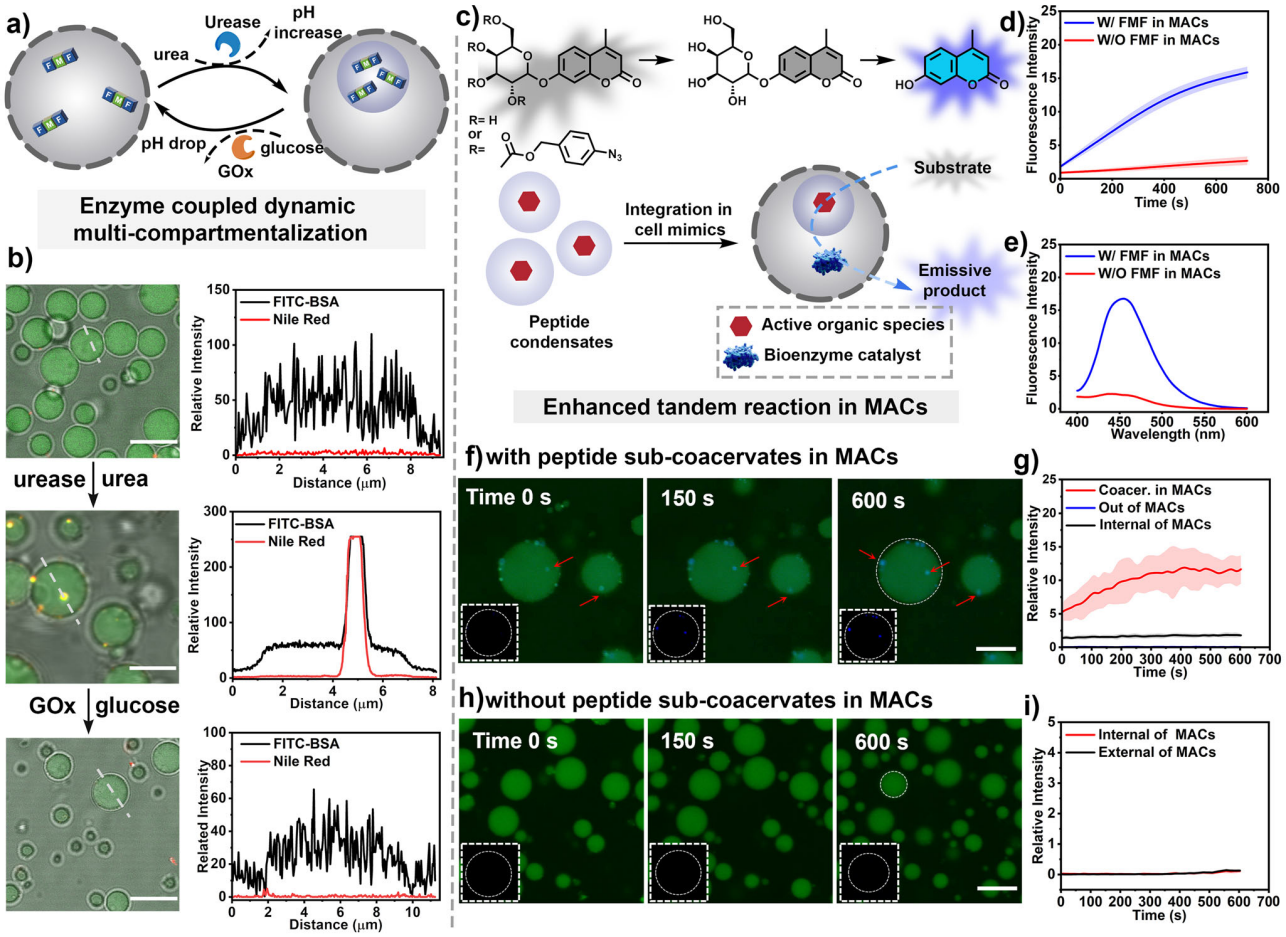
FMF coacervates as an adaptive and active model of biomolecule organelles for tandem catalysis in MACs. (a) Schematic representation of dynamic generation and disassembly of peptide‐based organelles using urease/urea and GOx/glucose‐based reactions. (b) Confocal images showed the absence, generation, and subsequent disassembly of subcellular organelles triggered by enzymatic reactions, and the corresponding fluorescence intensity comparison indicated reversible partition toward hydrophobic Nile red. Scale bar = 10 µm. (c) Schematic representation of a cascade reaction occurring in a multicompartment artificial cell (MACs). (d) Comparison of fluorescence intensity in the presence or absence of coacervates (0.114 mM Ph_3_P, 4 U mL^−1^ *β*‐Gal, 0.145 mM N_3_‑g‑coumarin). There is a significant increase in fluorescence emission (λ*_em_*  =  450 nm) when coacervate was present (10.9 mM FMF, pH∼7) in MACs. Data represent mean  ±  SD for n  =  3 independent samples; Error bars (blue shaded area) represent the standard deviation (n  =  3) from microplate reader analysis. (e) Corresponding fluorescence emission spectra of the reaction progress after 10 min incubation. (f) Confocal images show a gradual increase in fluorescence intensity mainly within the peptide coacervates (0.114 mM Ph_3_P, 4 U mL^−1^ *β*‐Gal, 0.145 mM N_3_‑g‑coumarin), scale bar = 10 µm. Blue colour indicated the generation of coumarin derivatives; (g) Comparison of fluorescence intensity (product coumarin derivatives) inside and outside cell‐mimics with organelles, data represent mean  ±  SD for n  =  3 representative microscopic images of view. (h) Confocal images show nearly no fluorescence increase both in the artificial cell without coacervate‐based sub‐organelles (0.114 mM Ph_3_P, 4 U mL^−1^ *β*‐Gal, 0.145 mM N_3_‑g‑coumarin), scale bar = 10 µm. (i) Comparison of fluorescence intensity (product coumarin derivatives) inside and outside the artificial cell without coacervate‐based sub‐organelles.

One of the hallmark features of living cells is their multi‐compartmentalization, which includes the integration of biomolecular condensates as sub‐organelles in a hierarchical organization manner [4, 5]. Having demonstrated the active formation of FMF coacervates regulated by enzymatic reaction and their utilities as biomimetic microreactors, we further explored their potential as models of active biomolecular condensates and catalytic centres in an artificial cell system (Figure 5a,c). First, the membrane‐bound artificial cells were constructed from the complex coacervation between quaternized amylose (Q‐Am) and negatively charged carboxymethylated amylose (C‐Am), followed by interfacial membranization with BSA modified MnO_2_ nanoparticles, as previously reported (Figure S77) [29, 48]. To realize the adaptive formation of FMF coacervates inside the artificial cells, FMF peptide solution, urease, and GOx were initially mixed with the complex coacervates between Q‐AM and C‐AM, and then interfacial membranization was executed (Figure 5a). The addition of urea increased the solution pH value, which triggered the phase‐separation of FMF inside the artificial cells (Figure 5b). The formation of internal FMF coacervate organelles was verified by the enhanced emission of hydrophobic Nile red, as the complex coacervate‐based artificial cells initially did not show partitioning toward hydrophobic dyes (Figure 5b and Figure S78). The active formation of FMF peptide coacervates as internal organelles was further confirmed by 3D construction of the artificial cells in the CLSM imaging (Figure S79). In addition, upon the addition of glucose, the oxidation of glucose produced gluconic acid, decreasing the solution pH value, which induced the dissolution of the coacervate organelles (Figure 5b). Enzymatic‐controlled dynamic generation and dissolution of active synthetic organelles in artificial cells resembled the adaptive formation of biomolecular condensates in living cells.

FMF coacervates demonstrated an ability to partition hydrophobic active species, while complex coacervates from Q‐AM and C‐AM displayed a concentrating effect toward bioenzymes due to their highly charged nature. We then investigate the potential of performing tandem reactions in the above “coacervate‐in‐coacervate” type multicompartment artificial cells (MACs) to emulate complex intracellular metabolism pathways. The introduction of peptide coacervates as sub‐organelles inside membrane‐bound artificial cells created a spatially organized reaction centre in the multi‐compartmentalized cell mimic. Then, triphenylphosphine (Ph_3_P) loaded FMF coacervates were integrated as sub‐organelles inside the *β*‑Gal encapsulated complex‐coacervate‐based artificial cells. N_3_‑g‑coumarin was fed as the substrate to initiate the cascade reaction (Figure 5c). The multi‐compartmentalized cell mimics (MACs) exhibited significantly increased reaction efficiency compared with the membrane‐bound artificial cells without peptide organelles (Figures 5d,e and Figure S80). CLSM revealed progressively intensifying blue fluorescence within the subcellular compartments, confirming successful in situ tandem catalysis inside the multi‐compartmentalized cell mimics (Figure 5f,g and Figure S81). The control artificial cells lacking embedded peptide organelles displayed low detectable blue fluorescence (Figure 5h,i). The ability to perform complex tandem reactions in multi‐compartmentalized cell mimics closely resembles the catalytic function of biomolecular condensates in living cells. This approach provides further guidance to extend the construction of complex biomimetic systems, in which controlled dynamics and catalytic functions are integrated to construct active materials with life‐like properties and functions [67, 69, 70].

## 3 Conclusion

In summary, we have presented the design and construction of short peptide‐based coacervate droplets with tunable phase‐separation behaviors, active formation, and application as a reaction centre for tandem catalysis. By tailoring the composition and sequence of short tripeptides, a sequence−structure−property relationship was investigated. Our findings indicate that the modulation of amino acid composition and peptide sequence significantly affects the phase‐separation behaviors, allowing the generation of coacervate droplets in a highly controlled manner. Short peptide coacervates exhibited highly tunable dynamics regulated by enzymatic reactions, closely resembling the active formation of biomolecular condensates. In addition, the peptide coacervates displayed an ability to partition and sequestration toward both hydrophobic and hydrophilic active species. The unique features allow short peptide coacervates to serve as versatile microreactors, promoting biochemical transformations, such as cleavage reactions and organic‐enzymatic tandem reactions in aqueous media. Upon integration within a model membrane‐bound synthetic cell, the peptide coacervates act as dynamic sub‐organelles and facilitate the execution of multi‐step reactions. The value of this work lies not in discovering a new peptide, a new enzyme, or a new reaction, but in demonstrating how minimal peptide condensates can be integrated into adaptive artificial cell architectures to execute complex chemical functions. The ability to finely control the dynamic assembly and disassembly through bio‐relevant factors promotes understanding of intracellular phase separation. The integration of functional synthetic sub‐organelles inside artificial cells opens an avenue to engineering more complex systems that can show life‐like properties. This research paves the way to develop more sophisticated biomimetic systems with potential applications in synthetic biology.

## Conflicts of Interest

The authors declare no conflicts of interest.

## Supporting information




**Supporting File 1**: anie71590‐sup‐0001‐SuppMat.docx.

## Data Availability

The data that support the findings of this study are available from the corresponding author upon reasonable request.
