# Supplementary material for: Sequence‐Modulated Active Tripeptide Condensates for Tandem Catalysis
Source: Angew Chem Int Ed Engl. 2026 Feb 17;65(13):e17620. doi: 10.1002/anie.202517620 (PMC13007586; doi:10.1002/anie.202517620)
Supplement: Supplementary file 1 — Supporting File 1: anie71590‐sup‐0001‐SuppMat.docx. [file ANIE-65-e17620-s001.docx]

Supplementary Information

**Sequence-Modulated Active Tripeptide Condensates for Tandem Catalysis**

Hao Han^+^, Siyu Song^+^, Jianqiang Wang, Tsvetomir Ivanov, Dongdong Zhou, Hao Su, Katharina Landfester*, Shoupeng Cao*

[^+^] These authors contributed equally to this work.

**Table of Contents**

1. Materials

2. Instruments

3. Synthesis procedures

4. Peptide droplet formulations and guest cargoes encapsulation

5. Biomimicry application with peptide droplets

6. As sub-organelles inside complex coacervate-based synthetic cells

7. The assembly behaviors of tripeptides

8. FMF tripeptide coacervates and active condensates microreactors

9. Supplementary References

## 1. Materials

Boc-*L*-Phenylalanine (Boc-Phe-OH, 98%, Innochem), Boc-*L*-Glycine (Boc-Gly-OH, 99%, Adamas), Boc-*L*-Alanine (Boc-Ala-OH, 99%, Adamas), Boc-*L*-Valine (Boc-Val-OH, 99%, Adamas), Boc-*L*-Leucine (Boc-Leu-OH, 99%, Adamas), Boc-*L*-Isoleucine (Boc-Ile-OH, 99%, Adamas), Boc-*L*-Methionine (Boc-Met-OH, 99%, Adamas), Boc-*L*-Tyrosine (Boc-Try-OH, 98%, Innochem), *L*-Phenylalanine methyl ester hydrochloride (NH_2_-Phe-OMe, 98%, Innochem), *L*-Glycine methyl ester hydrochloride (NH_2_-Gly-OMe, 99%, Innochem), *L*-Alanine methyl ester hydrochloride (NH_2_-Ala-OMe, 98%, Innochem), *L*-Valine methyl ester hydrochloride (NH_2_-Val-OMe, 97%, Innochem), *L*-Leucine methyl ester hydrochloride (NH_2_-Leu-OMe, 98%, leyan), *L*-Isoleucine methyl ester hydrochloride (NH_2_-Ile-OMe, 97%, Adamas-beta), *L*-Methionine methyl ester hydrochloride (NH_2_-Met-OMe, 99%, Aladdin), *L*-Tyrosine methyl ester hydrochloride (NH_2_-Try-OMe, 98%, leyan), 1-hydroxybenzotriazole (HOBt, 99%, Adamas-beta), 2-(1H-benzotriazole-1-yl)-1,1,3,3-tetramethyluronium tetra fluoroborate (HBTU, 99%, Adamas-beta), N,N-diisopropylethylamine (DIPEA, 99%, Adamas-beta), pyridine (99.8%, Aladdin), N,N-Dimethylformamide (DMF, 99.5%, Macklin), Triethylamine (TEA, 99%, Innochem), Triphenylphosphine (pph_3_, 99%, Aladdin), Bovine albumin (BSA, 98%, Innochem), *β*-Gal (1 kU /2 mL, Sigma), 4-Methylumbelliferyl beta-D-galactoside (99%, Macklin), (4-Azidophenyl)methanol (≥95%，Chemxyz), Glucose oxidase (GOx, 130 U mg^-1^, Innochem), Urease 200~300 U mg^-1^, Macklin), Urea (99%, Macklin), 1,2-Hexanediol (HDO, 98%, Innochem), D-(+)-Glucose (≥99.5%, Aladdin), 4 M hydrogen chloride solution in dioxane (Innochem), CDCl_3_ (99.8% D, Innochem), DMSO-*d_6_* (99.8% D, Adamas-beta), HEPES buffer solution (1 M, Macklin), phosphate-buffered saline (PBS, Macklin). All other solvents, chemicals and salts used were purchased without further treatment unless otherwise stated.

## 2. Instruments

**Nuclear magnetic resonance (NMR) spectroscopy:** Routine proton nuclear magnetic resonance (1H NMR) measurements were performed on a Bruker Avance III 400 MHz Ultra shield TM spectrometer equipped with a Bruker Sample Case autosampler, using CDCl_3_ or DMSO-*d_6_* as solvent and TMS as internal standard.

**High performance liquid chromatography (HPLC)**: Analytical high performance liquid chromatography (HPLC) was recorded on a Essentia LC-16 instrument (Shimadzu, Japan) using a Innoval ODS-2 C18 column (5 µm, 250 × 4.6 mm) with a flow rate of 1 mL/min for 15 mins from 15% acetonitrile to 100% acetonitrile, holding for 5 min, and monitored at 220 nm. The HPLC mobile phases were water and ACN containing 0.1% v/v TFA.

**High Performance Liquid Chromatography-Mass Spectrometry (HPLC-MS):** The final product was analyzed for molecular weight using liquid mass spectrometry. Mass spectra (MS) were acquired on an ISQEM mass spectrometer (Thermo Scientific, American) employing the Electrospray Ionization (ESI) technique to generate ions from the sample before analysis. The MS mobile phases were water and ACN containing 0.1% v/v formic acid.

**Microplate reader:** Catalytic reactions were evaluated using a microplate reader (TECAN, infinite M1000). Reaction progress was monitored by the absorbance or fluorescence signal on the microplate reader.

**Bright-field microscopy imaging:** The bright-field images were captured with the Leica Mateo TL microscope (MATEO TL RUO).

**Confocal scanning microscopy imaging:** confocal microscopy was performed using the Leica stellaris 5.

**UV-vis**: The analysis of UV-vis spectroscopy was using Perkin Elmer Lambda 650.

**Fluorescence Spectrometer**: The analysis of Fluorescence Spectrometer was using Fluorospectrophotometer F98.

## 3. Synthesis procedures

**Synthesis of XFF**

###

**Scheme S1** Synthesis route of tripeptide XFF. Derivatives of diphenylalanine-based compounds were synthesized via simple multi-step reactions according to literature reports with slight modifications^[1,2]^.

### **MFF:** The synthesis of MFF was realized in a four-step reaction as below, as previously reported ^[1]^. The same procedures were applied to the synthesis of **GFF, AFF, VFF, IFF, LFF, FFF and YFF**.

**(i)** First, N-(tert-butoxycarbonyl)-L-phenylalanine (Boc-Phe-OH) (5.8 g, 22 mmol), HBTU (8.26 g, 22 mmol) and HOBt (2.94 g, 22 mmol) were dissolved in 30 mL DMF in a round-bottom flask and the mixture was stirred with a magnetic stirrer. N, N-diisopropylethylamine (DIPEA) (7 mL, 44 mmol), and then L-phenylalanin-methylester-hydrochlorid (4.24 g, 20 mmol) were added and the reaction mixture was stirred for 24 h at room temperature. The reaction mixture was poured into 800 mL of water. The white precipitate was collected by filtration and washed with water. The white solid was dissolved in DCM, washed twice with 1% HCl and saturated sodium bicarbonate to remove the remaining raw materials and salt, and finally washed with saturated brine, dried over MgSO_4_, and then the solvent was removed under vacuum to obtain the solid product 3.64 g (Boc-FF).

**(ii)** The intermediate compound (3.6 g) was dissolved in 25 mL of 4 M hydrogen chloride solution in dioxane for deprotection. After stirring for 3 hours, the solvent was evaporated on a rotary evaporator to give an oily residue. Diethyl ether was added to the flask, and the contents were stirred gently. A white precipitate formed and was separated by centrifugation, yielding about 3.2 g of a white product (FF).

**(iii)** Boc-*L*-Methionine (Boc-Met-OH) (137 mg, 0.55 mmol), HBTU (208.6 mg, 0.55 mmol) and HOBt (74.3 mg, 0.55 mmol) were dissolved in 0.75 mL DMF in a round-bottom flask and the mixture was stirred with a magnetic stirrer. N, N-diisopropylethylamine (DIPEA) (175 μL, 1.1 mmol), and then FF (189 mg, 0.5 mmol) were added and the reaction mixture was stirred for 24 h at room temperature. The reaction mixture was poured into 20 mL of water. The white precipitate was collected by filtration and washed with water. The white solid was dissolved in DCM, washed twice with 1% HCl and saturated sodium bicarbonate to remove the remaining raw materials and salt, and finally washed with saturated brine, dried over MgSO_4_, and then the solvent was removed under vacuum to obtain 220 mg solid product (Boc-MFF).

**(iv)** Finally, the intermediate compound (100 mg) was dissolved in 2 mL of 4 M hydrogen chloride solution in dioxane for deprotection. After stirring for 3 hours, the solvent was evaporated on a rotary evaporator to give an oily residue. Diethyl ether was added to the flask and the contents were stirred gently. A white precipitate formed and was separated by centrifugation, yielding about 80 mg of a white product (MFF). The product was characterized by NMR and HPLC-MS.^1^H NMR (400 MHz, DMSO-*d_6_*): *δ* 8.73 (d, 2H), 8.26 (d, 2H), 7.28 (d, *J* = 3.0 Hz, 10H), 4.49 (d, *J* = 5.4 Hz, 2H), 3.78 (s, 1H), 3.58 (d, *J* = 1.9 Hz, 3H), 3.16 – 2.88 (m, 3H), 2.88 – 2.72 (m, 1H), 2.45 (t, *J* = 8.5 Hz, 2H), 2.02 (t, 1H). HPLC-MS(EI), calculated for C_24_H_31_N_3_O_4_S:458.2 (M+H^+^); found 458.3 (M ^+^).

###
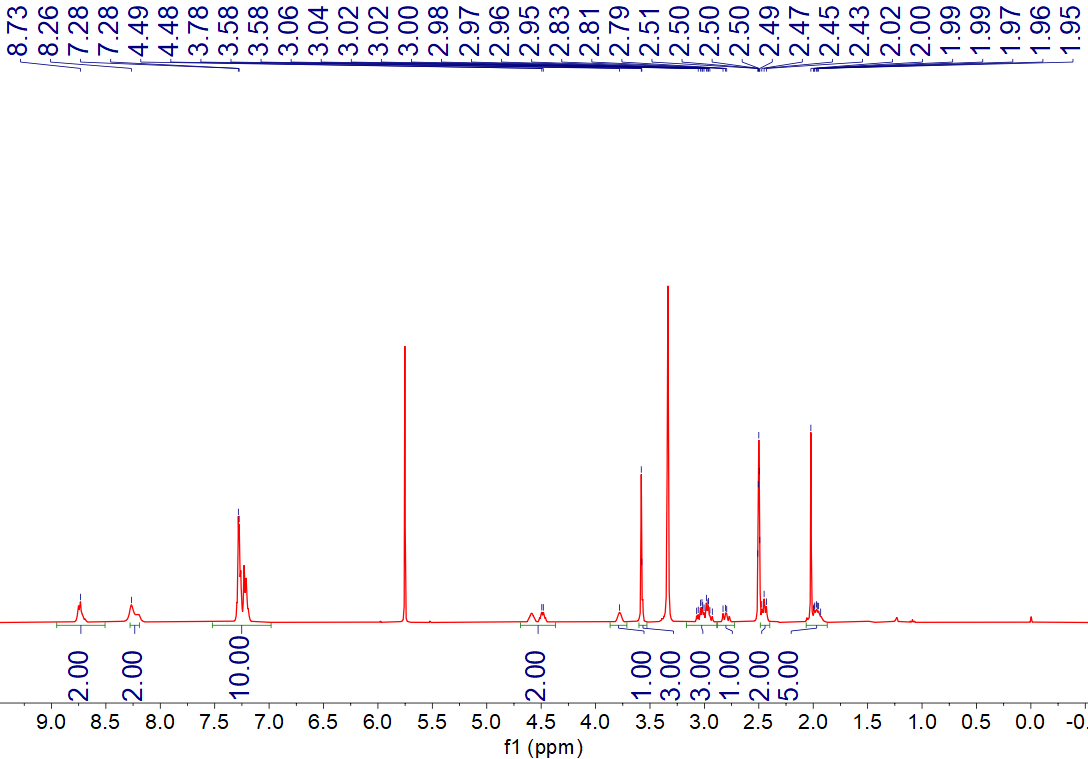


###
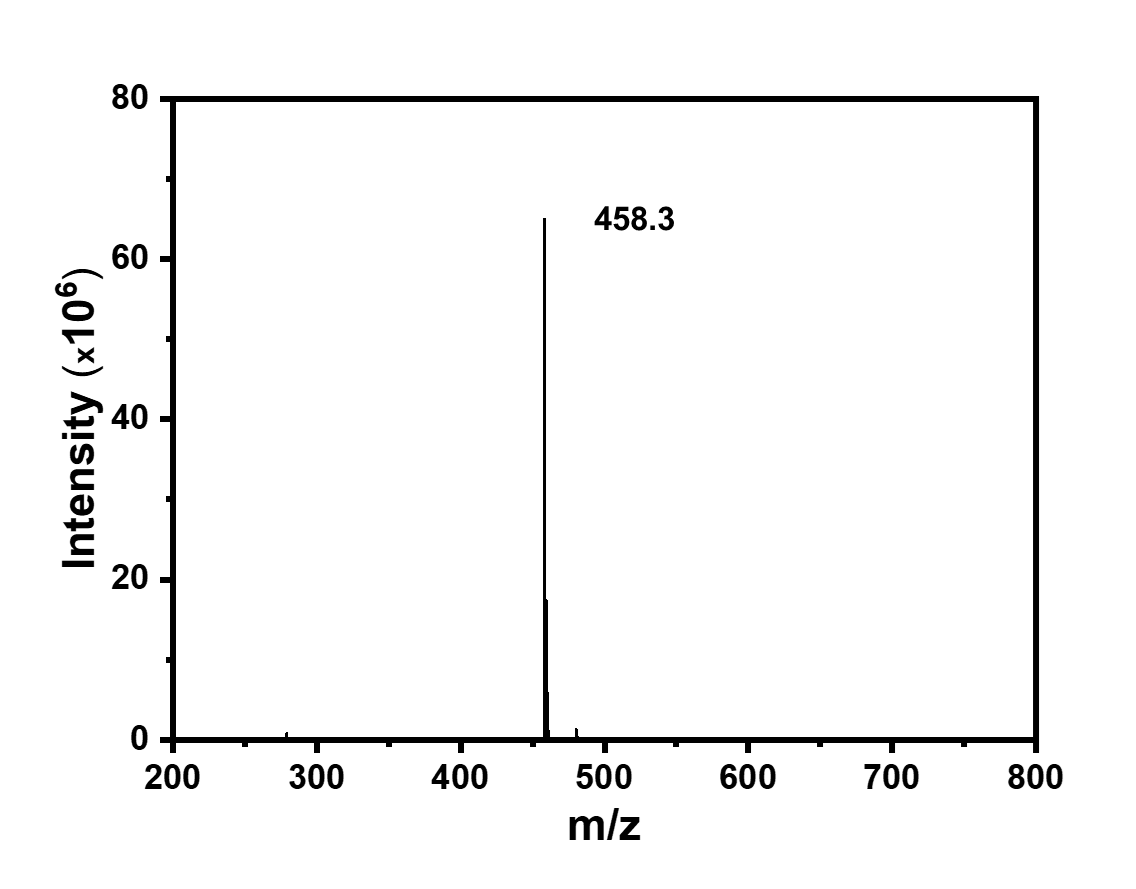


**Figure S1.** ^1^H NMR, and LC-MS spectra of MFF (NMRs in DMSO-*d_6_*)

**GFF** ^1^H NMR (400 MHz, DMSO-*d_6_*): *δ* 8.78 (dd, *J* = 24.1, 8.0 Hz, 2H), 8.18 – 8.13 (m, 2H), 7.33 – 7.17 (m, 10H), 4.61 (m, *J* = 9.1, 4.3 Hz, 1H), 4.49 (td, *J* = 8.3, 5.9 Hz, 1H), 3.59 (s, 3H), 3.56 (s, 2H), 3.09 – 2.94 (m, 3H), 2.75 – 2.68 (m, 1H).^13^C NMR (101 MHz, DMSO-*d_6_*): *δ* 171.65, 170.85, 165.51, 137.40, 137.12, 129.22, 129.09, 128.27, 128.20, 128.07, 126.57, 126.37, 66.34, 53.86, 53.74, 51.88, 38.24, 37.80, 36.51. HPLC-MS(EI), calculated for C_21_H_25_N_3_O_4_:384.2 (M+H^+^); found 384.3 (M ^+^).

###
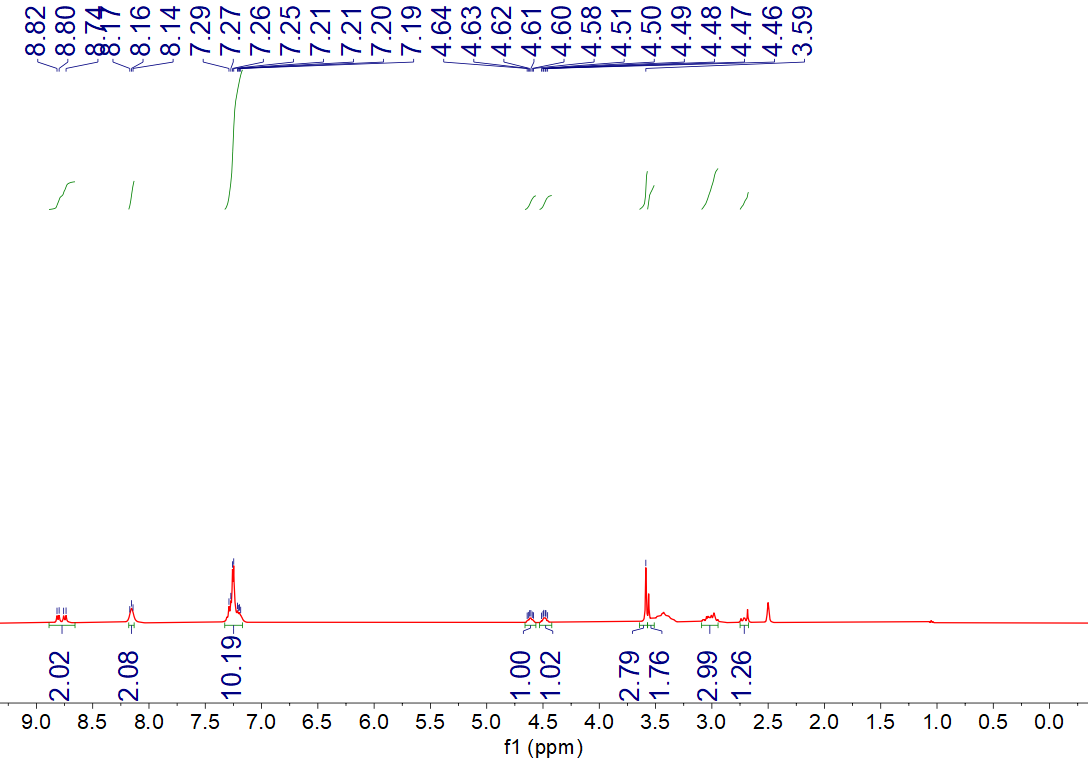


###
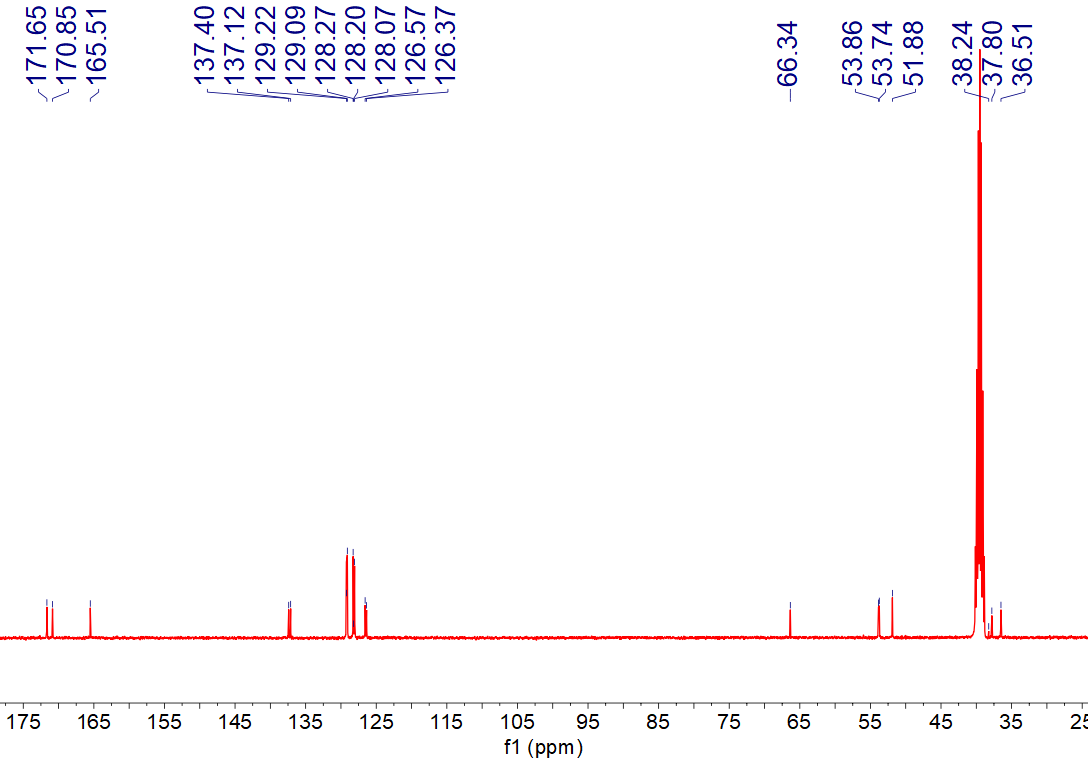


###
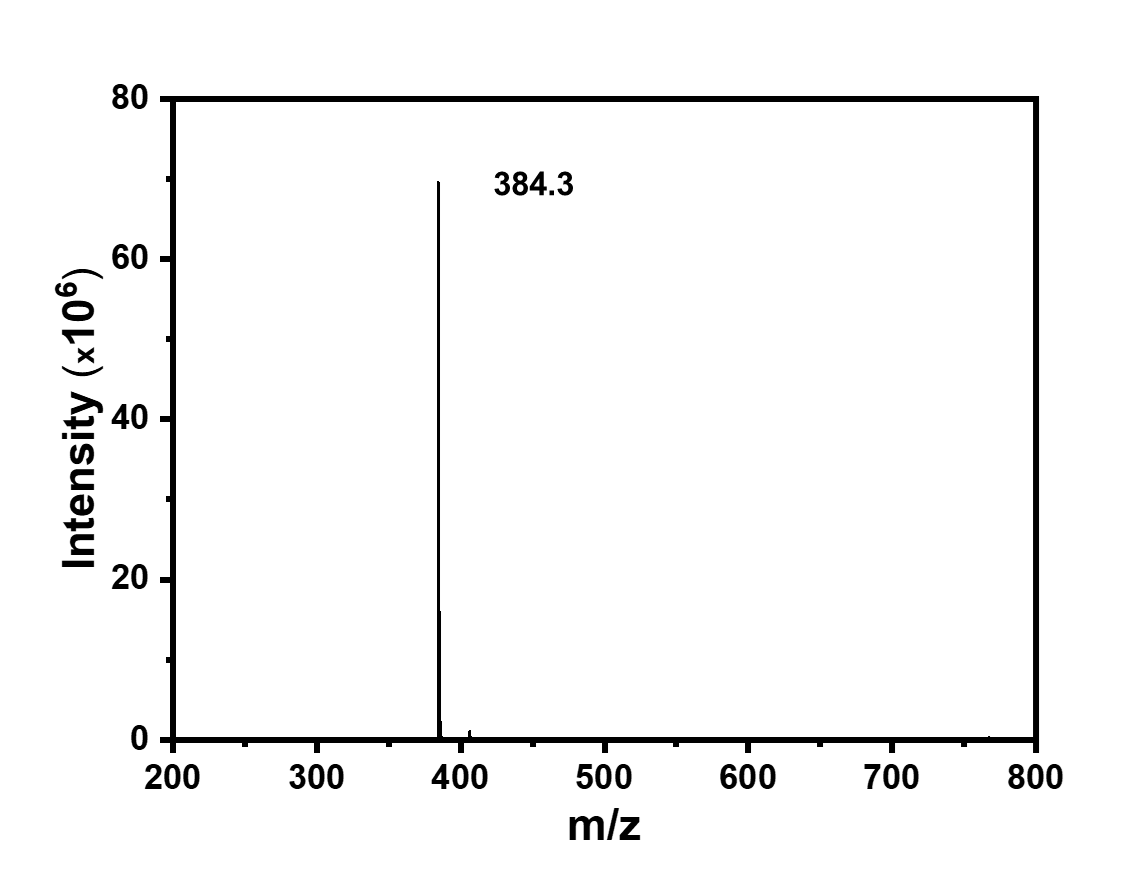


**Figure S2.** ^1^H NMR, ^13^C NMR and LC-MS spectra of GFF (NMRs in DMSO-*d_6_*)

**AFF:** ^1^H NMR (400 MHz, DMSO-*d_6_*): *δ* 8.75 (dd, *J* = 21.8, 8.0 Hz, 2H), 8.27– 8.11 (m, 2H), 7.38– 7.15 (m, 10H), 4.51 (m, *J* = 24.6, 8.6, 5.2 Hz, 2H), 3.83 – 3.67 (m, 1H), 3.57 (s, 3H), 2.99 (m, *J* = 14.7, 9.6, 5.1 Hz, 3H), 2.85 – 2.60 (m, 1H), 1.32 (d, *J* = 6.9 Hz, 3H).^13^C NMR (101 MHz, DMSO-*d_6_*): *δ* 171.66, 170.83, 169.34, 137.49, 137.11, 129.16 (d, *J* = 10.0 Hz), 128.25, 128.11, 126.54, 126.36, 54.27, 53.69, 51.86, 47.95, 37.37, 36.53, 17.17. HPLC-MS(EI), calculated for C_22_H_27_N_3_O_4_:398.2 (M+H^+^): found 398.3 (M ^+^).

###
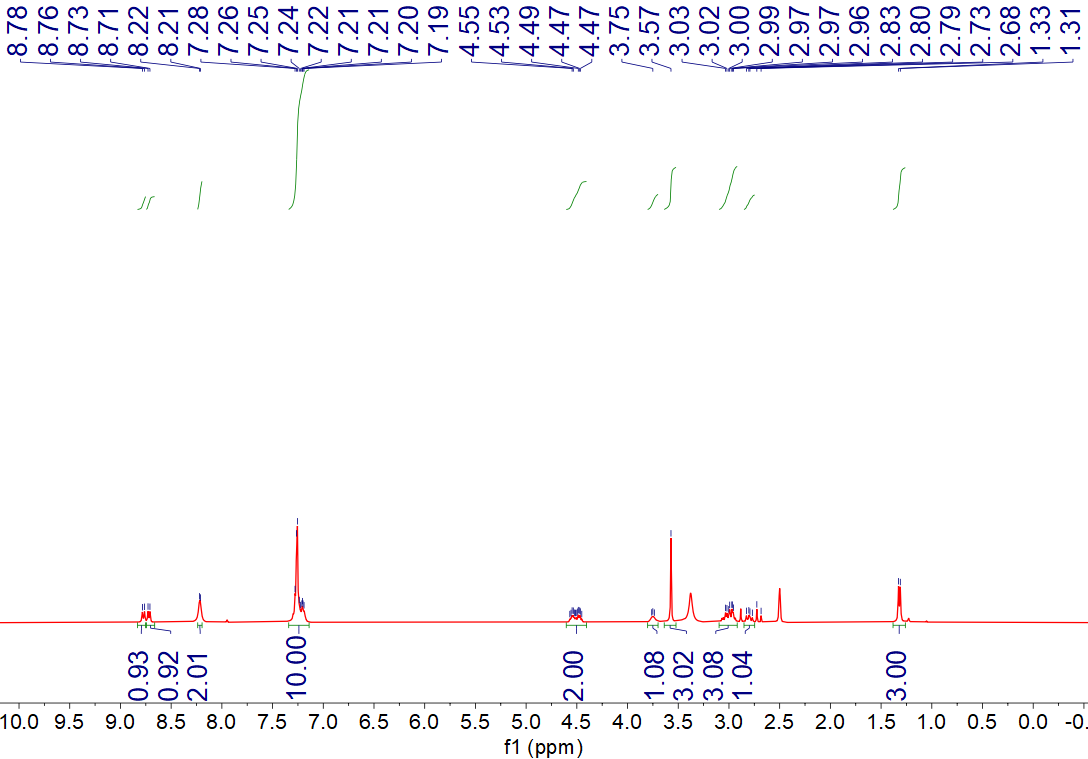


###
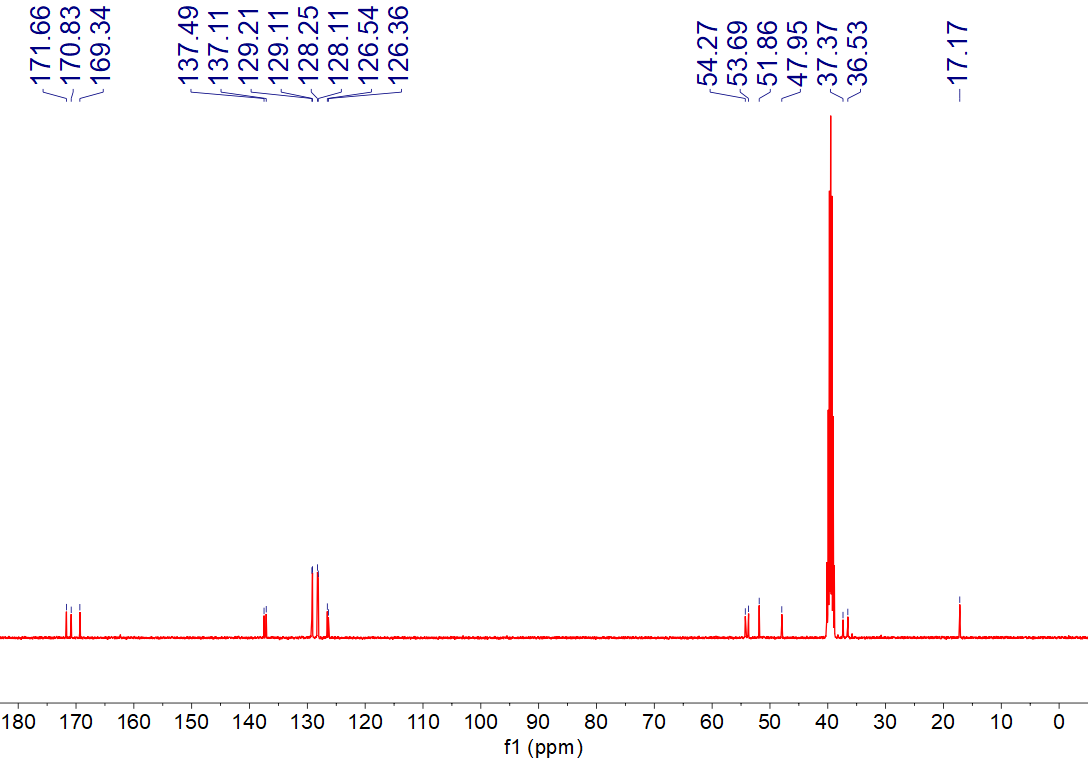


###
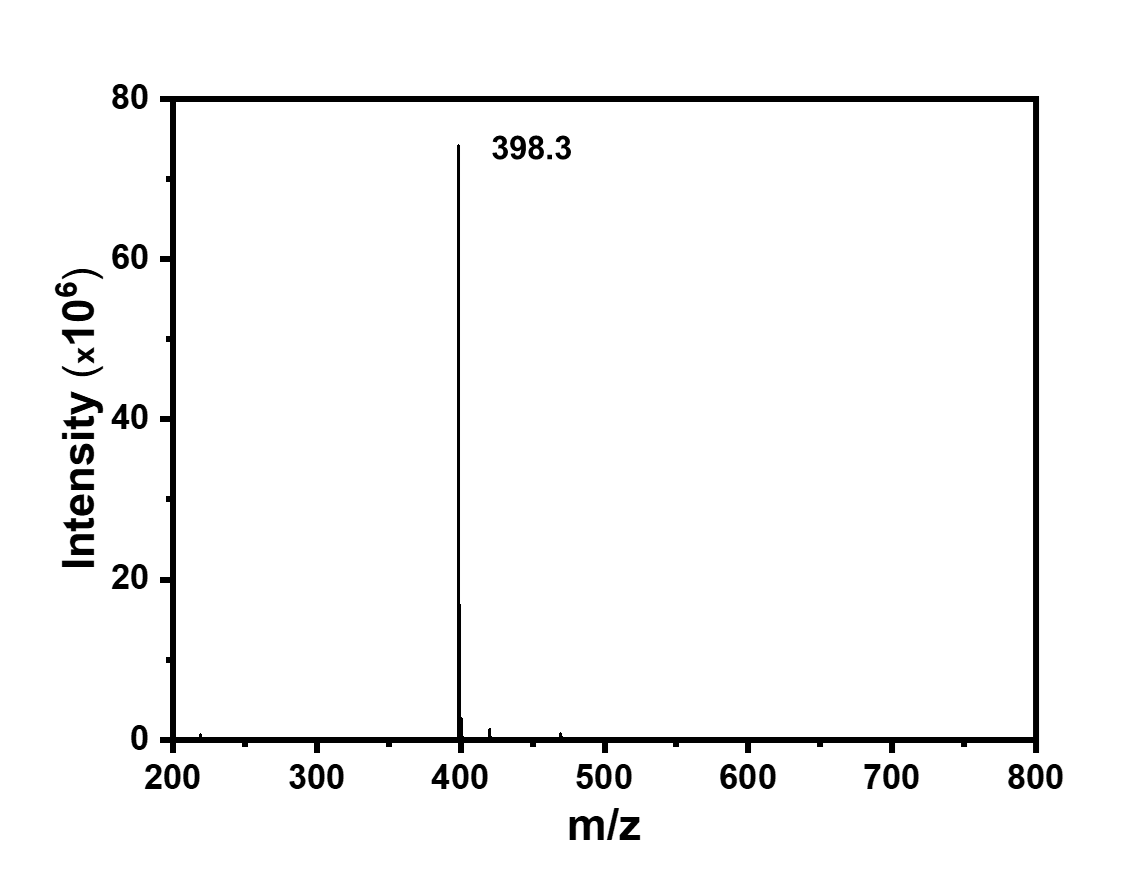


**Figure S3.** ^1^H NMR, ^13^C NMR and LC-MS spectra of AFF (NMRs in DMSO-*d_6_*)

**VFF：**^1^H NMR (400 MHz, DMSO-*d_6_*): *δ* 8.72 (dd, *J* = 7.9, 2.7 Hz, 2H), 8.20 (d, *J* = 5.3 Hz, 2H), 7.32 – 7.17 (m, 10H), 4.60 (m, J = 8.4, 5.2 Hz, 1H), 4.48 (m, *J* = 8.4, 5.6 Hz, 1H), 3.56 (s, 3H), 3.52 – 3.43 (m, 1H), 3.10 – 2.91 (m, 3H), 2.83 (dd, *J* = 13.9, 8.7 Hz, 1H), 2.08 (dd, *J* = 13.3, 6.7 Hz, 1H), 0.86 (m, *J* = 6.4 Hz, 6H).^13^C NMR (101MHz, DMSO-*d_6_*): *δ* 171.57, 170.67, 167.65, 137.39, 137.12, 129.22, 129.02, 128.20, 128.10, 126.48, 126.33, 72.16, 70.52, 60.16, 56.98, 53.99, 53.55, 51.84, 43.62, 38.24, 37.55, 36.47, 29.81, 18.36, 17.46. HPLC-MS(EI), calculated for C_24_H_31_N_3_O_4_:426.2 (M+H^+^): found 426.3 (M ^+^).

###
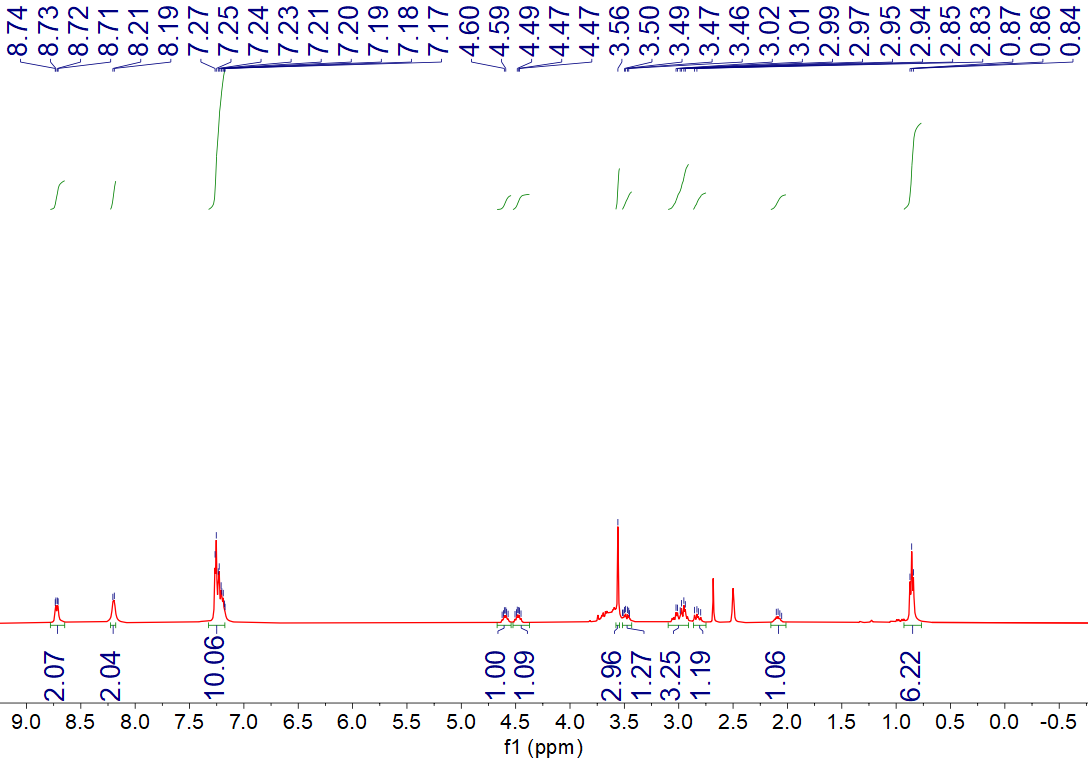


###
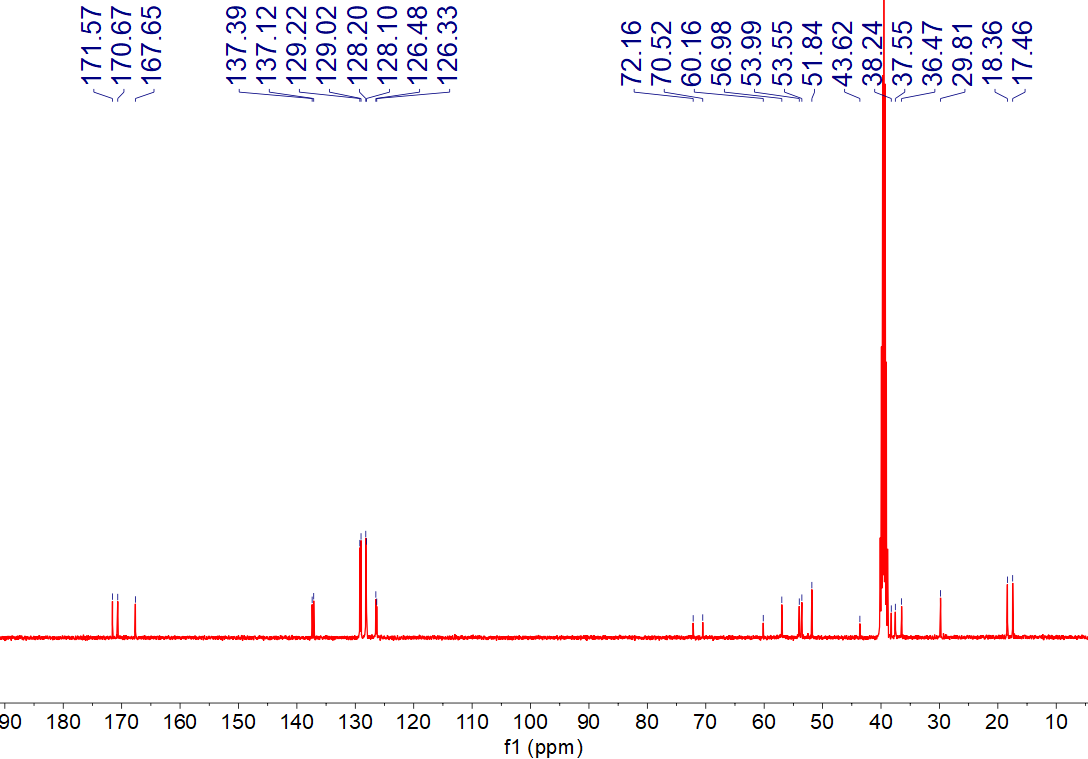


###
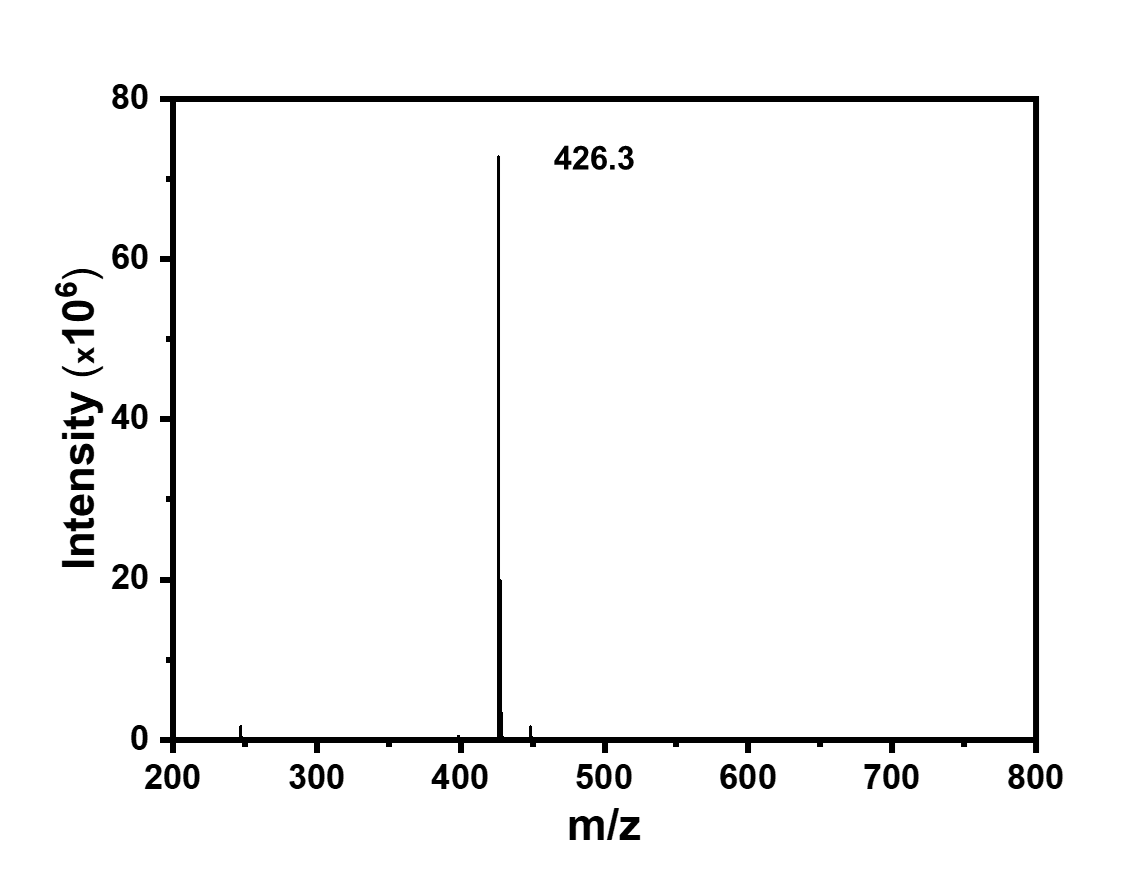


**Figure S4.** ^1^H NMR, ^13^C NMR and LC-MS spectra of VFF (NMRs in DMSO-*d_6_*)

**LFF:** ^1^H NMR (400 MHz, DMSO-*d_6_*): *δ* 8.84 (d, *J* =8.2 Hz, 1H), 8.71 (d, *J* =7.6 Hz, 1H), 8.28 (s, 2H), 7.25 (t, *J* =5.4 Hz, 10H), 4.52 (m, *J* =41.8, 8.3, 2.9 Hz, 2H), 3.75–3.65 (m, 1H), 3.56 (s, 3H), 3.10–2.77 (m, 4H), 1.70–1.57 (m, 1H), 1.51 (t, *J* =7.1 Hz, 2H), 0.85 (dd, *J* =6.4, 3.3 Hz, 6H).^13^C NMR (101 MHz, DMSO-*d_6_*): *δ* 172.08, 171.12, 169.30, 137.92, 137.63, 129.69, 129.55, 128.70, 128.59, 126.98, 126.80, 72.64, 71.00, 60.64, 54.53, 54.11, 52.32, 51.18, 44.09, 38.72, 37.94, 36.98, 23.81, 23.26, 22.33. HPLC-MS(EI), calculated for C_25_H_33_N_3_O_4_: 440.3 (M+H^+^): found 440.4 (M ^+^).

###
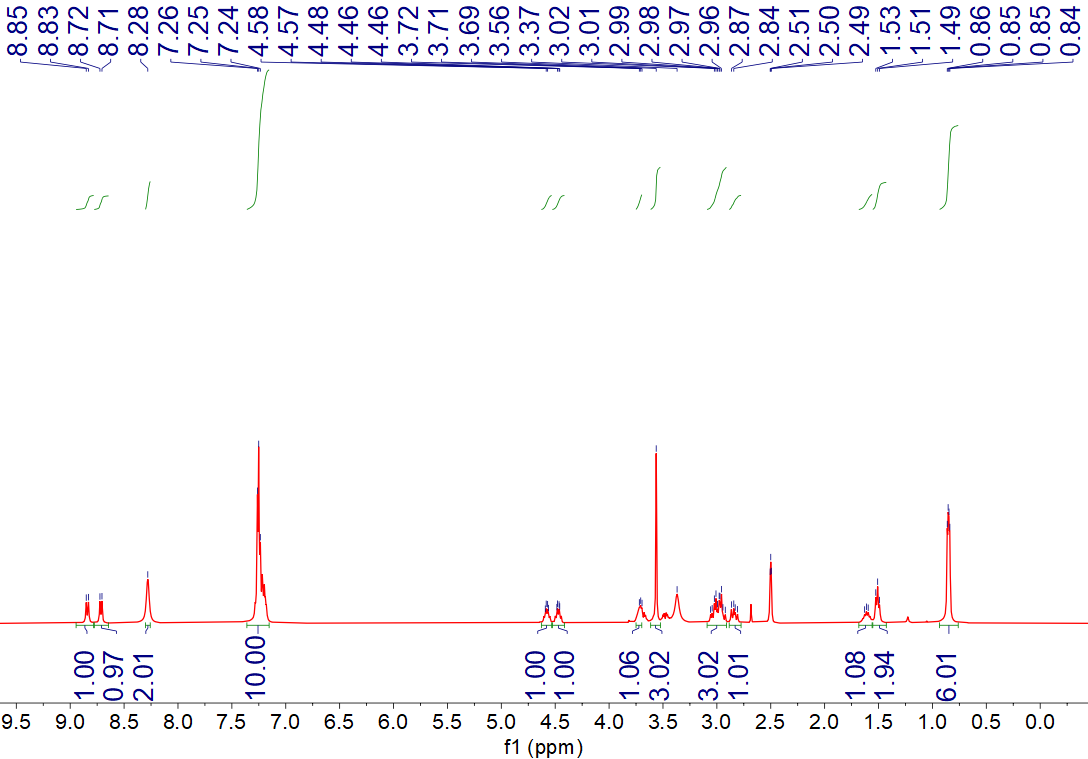


###
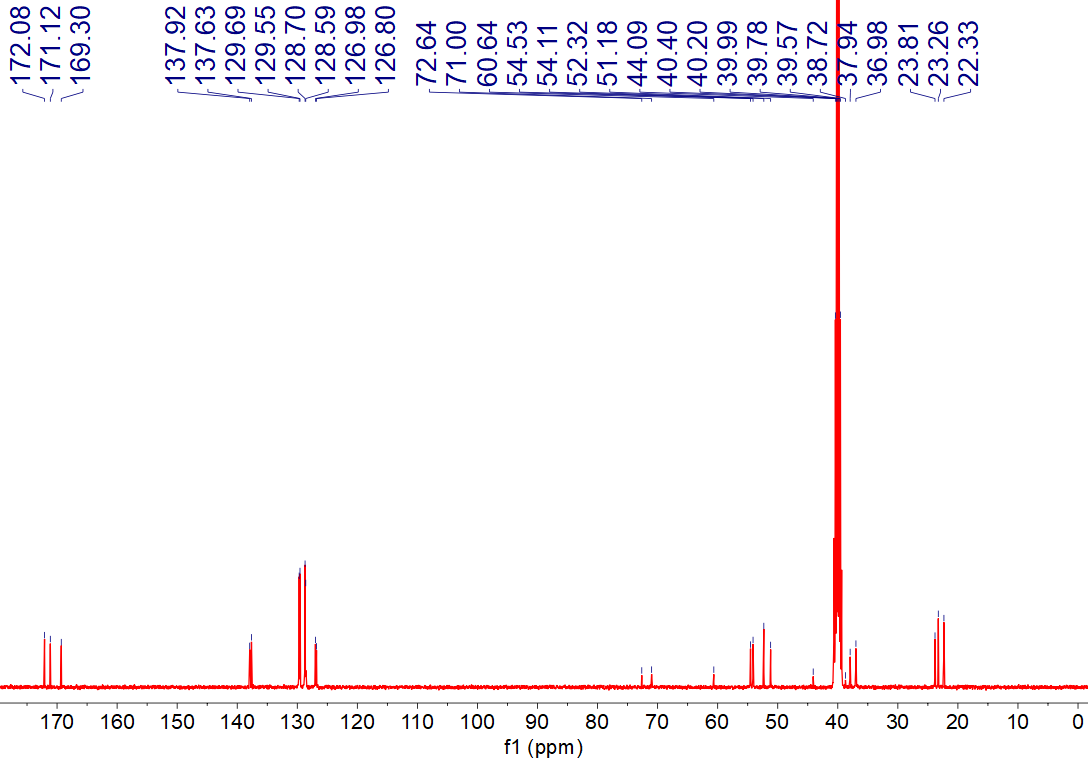


###
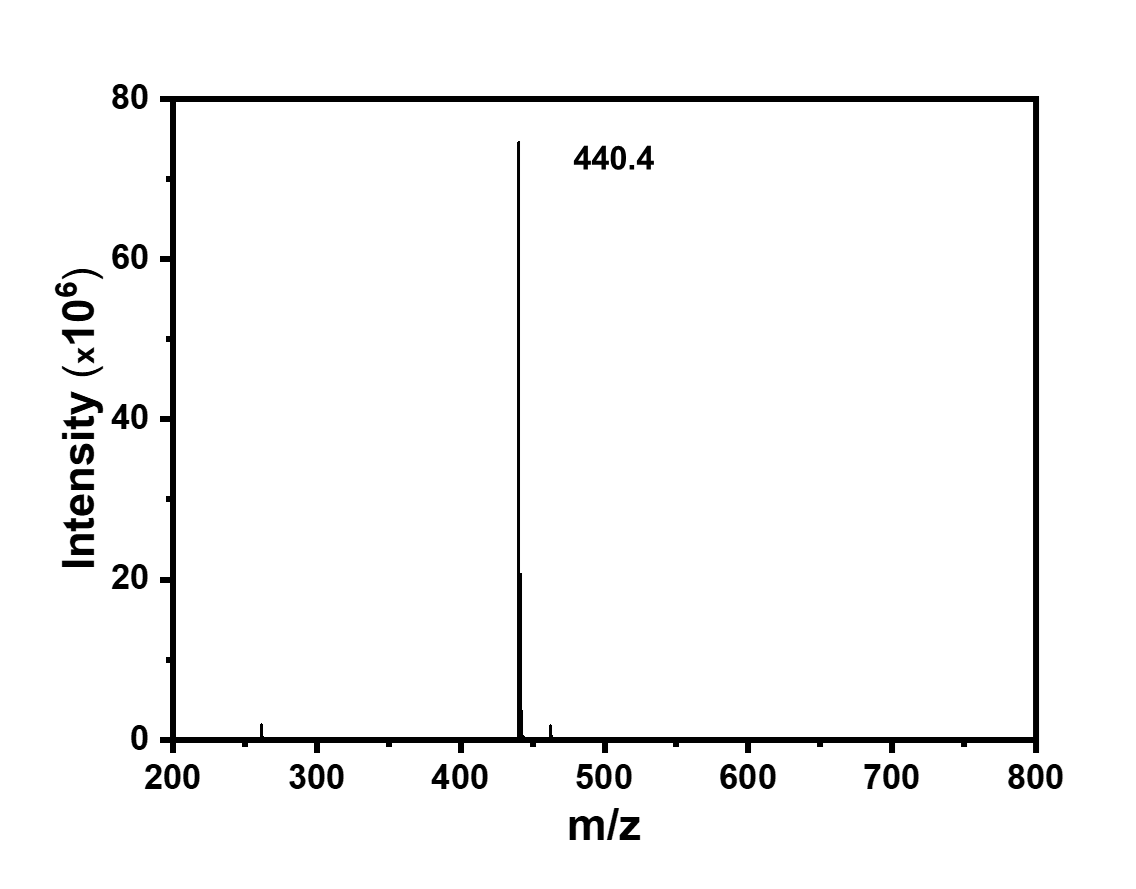


**Figure S5.** ^1^H NMR, ^13^C NMR and LC-MS spectra of LFF (NMRs in DMSO-*d_6_*)

**IFF：**^1^H NMR (400 MHz, DMSO-*d_6_*): *δ* 8.69 (dd, J = 15.8, 7.9 Hz, 2H), 8.20 (s, 2H), 7.29 – 6.96 (m, 10H), 4.60 (dd, *J* = 8.4, 5.3 Hz, 1H), 4.47 (m, *J* = 8.4, 4.2 Hz, 1H), 3.69 (dd, *J* = 12.9, 4.8 Hz, 1H), 3.56 (s, 3H), 3.09 – 2.80 (m, 4H), 1.81 (t, *J* = 6.2 Hz, 1H), 1.44 (m, *J* = 10.9, 3.6 Hz, 1H), 1.11 – 0.99 (m, 1H), 0.81 (dd, *J* = 7.1, 2.8 Hz, 6H). ^13^C NMR (101 MHz, DMSO-*d_6_*): *δ* 171.54, 170.58, 167.61, 137.34, 137.10, 129.20, 128.98, 128.18, 128.08, 126.46, 126.30, 72.14, 70.50, 66.33, 60.15, 56.28, 53.87, 53.53, 51.83, 43.60, 38.23, 37.55, 36.47, 36.20, 23.70, 14.49, 11.17. HPLC-MS(EI), calculated for C_25_H_33_N_3_O_4_:440.3 (M+H^+^); found 440.3 (M ^+^).

###
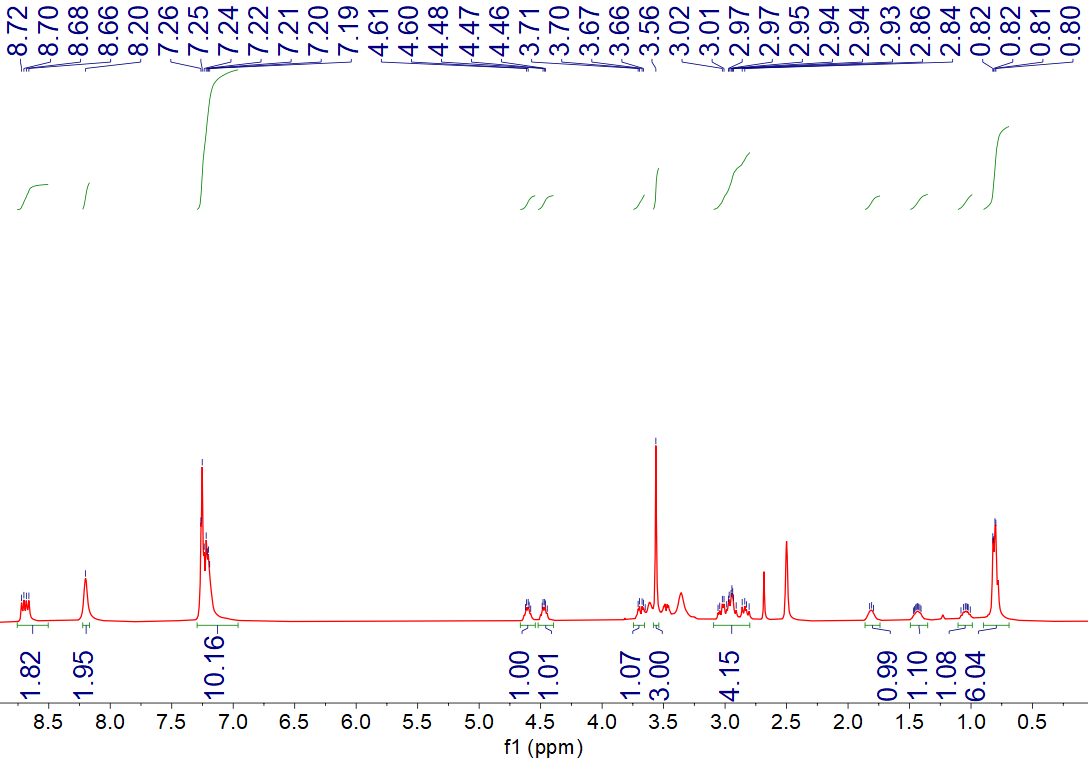


###
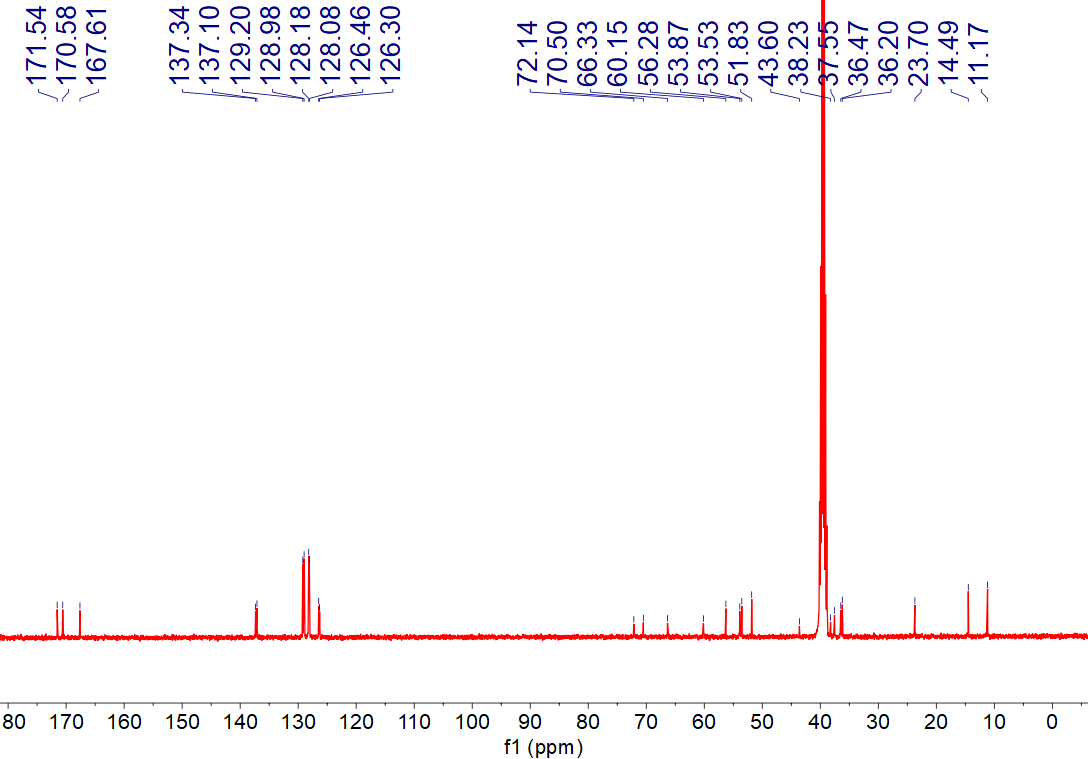


###
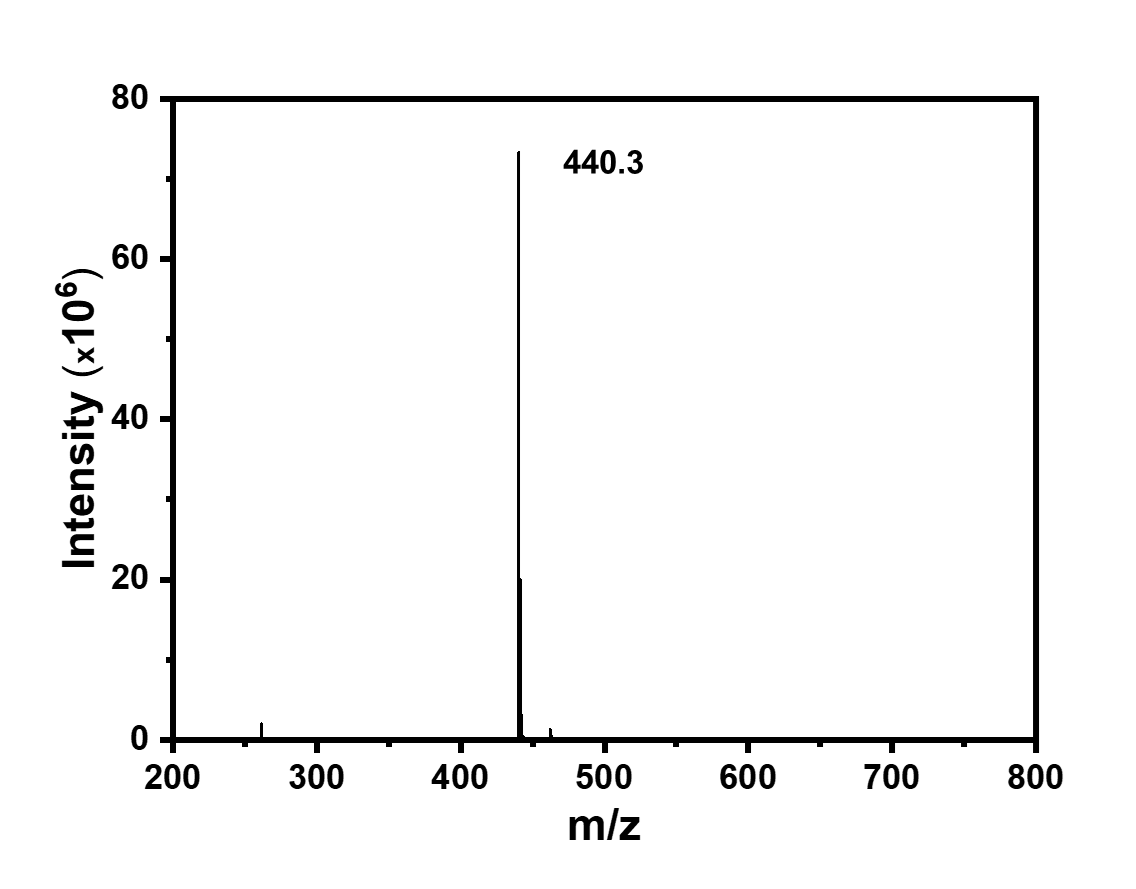


**Figure S6.** ^1^H NMR, ^13^C NMR and LC-MS spectra of IFF (NMRs in DMSO-*d_6_*)

**FFF:** The synthesis steps are the same as previously reported^[1,2]^.

^1^H NMR (400 MHz, DMSO-*d_6_*): *δ* 9.03 (t, *J* = 8.8 Hz, 1H), 8.79 (d, *J* = 7.2 Hz, 1H), 8.18 (s, 2H), 7.26 (m, *J* = 10.4, 4.0, 3.2 Hz, 15H), 4.55 (m, *J* = 24.8, 8.4, 5.5 Hz, 2H), 4.00 (dd, *J* = 7.9, 4.8 Hz, 1H), 3.58 (s, 3H), 3.22 – 2.78 (m, 6H). HPLC-MS(EI), calculated for C28H31N3O4: 474.3 (M+H^+^); found 474.3 (M ^+^).

###
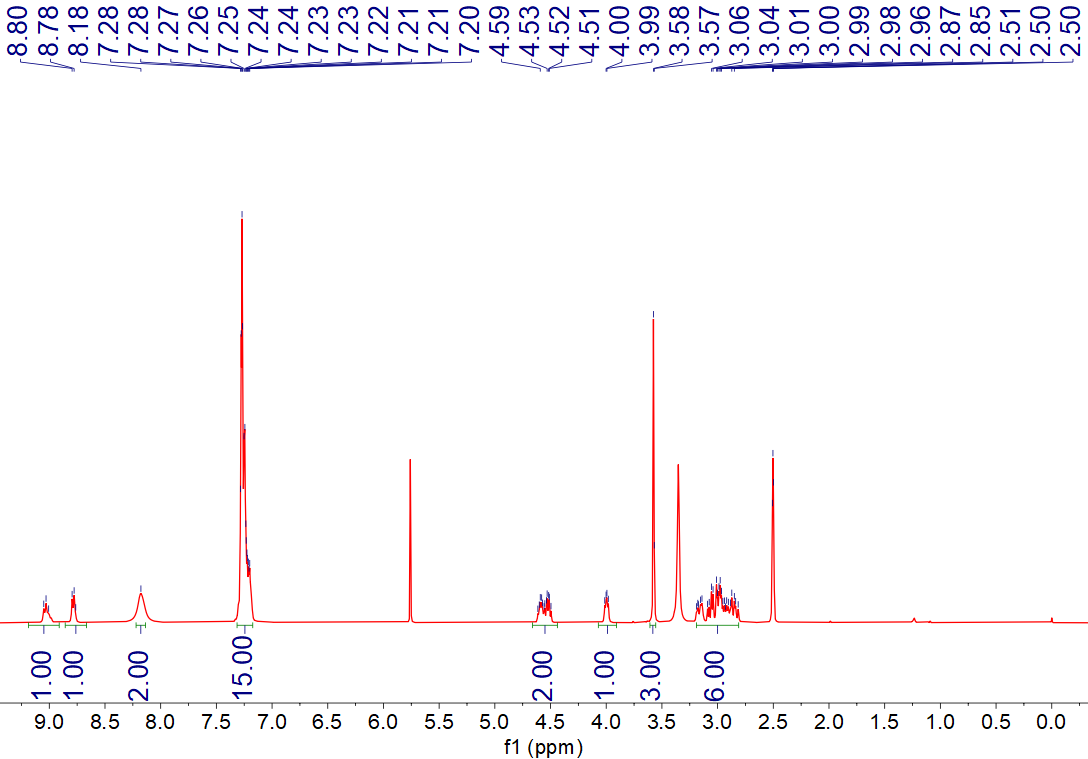


###
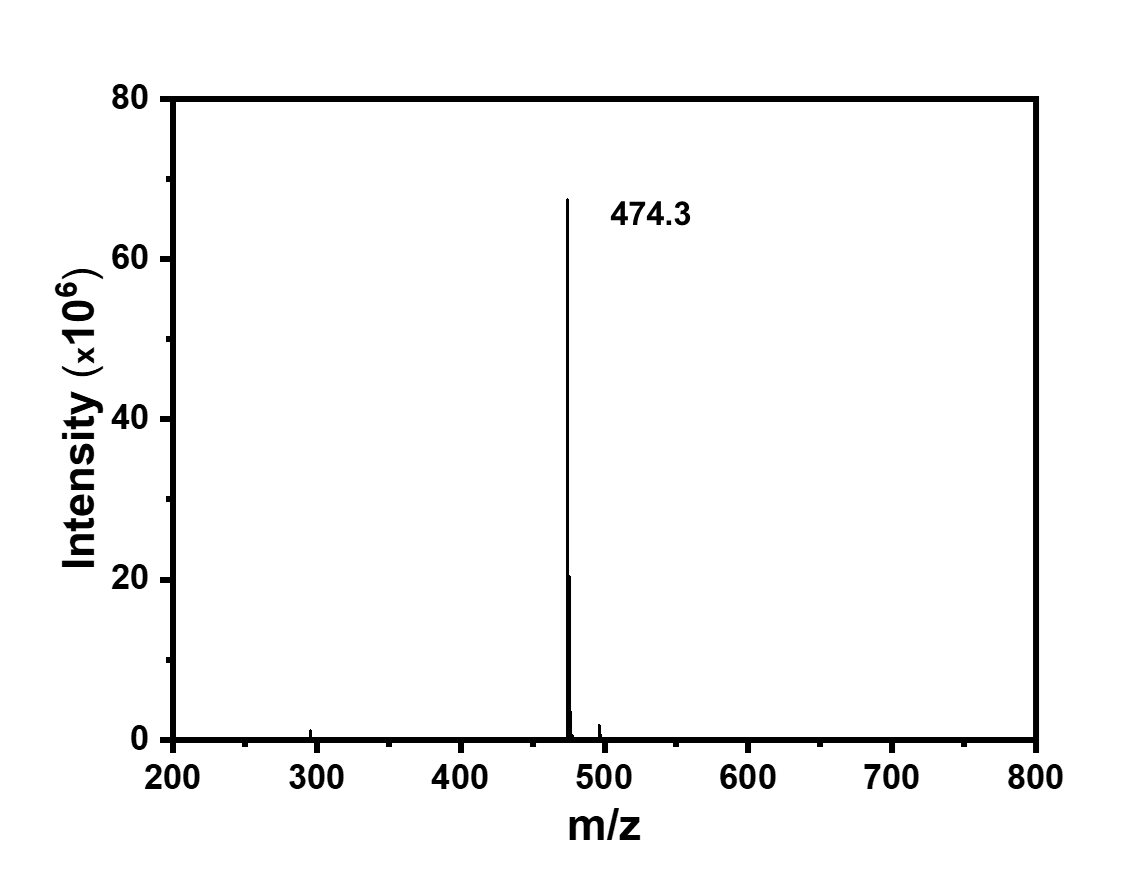


**Figure S7.** ^1^H NMR and LC-MS spectra of FFF (NMRs in DMSO-*d_6_*)

**YFF:**^1^H NMR (400 MHz, DMSO-*d_6_*): *δ* 9.41 (s, 1H), 8.97 (d, *J* = 8.3 Hz, 1H), 8.76 (d, *J* = 7.5 Hz, 1H), 8.07 (s, 2H), 7.37 – 7.14 (m, 10H), 7.05 (d, *J* = 8.2 Hz, 2H), 6.68 (d, *J* = 8.4 Hz, 2H), 4.54 (m, J = 21.7, 8.2, 4.8 Hz, 2H), 3.89 (dd, *J* = 8.2, 4.6 Hz, 1H), 3.57 (s, 3H), 3.10 – 2.93 (m, 4H), 2.80 (m, *J* = 21.9, 14.1, 8.5 Hz, 2H).^13^C NMR (101 MHz, DMSO-*d_6_*): *δ* 171.58, 170.69, 167.96, 156.56, 137.40, 137.05, 130.64, 129.26, 129.10, 128.27, 128.12, 126.56, 126.37, 124.67, 115.28, 54.20, 53.66, 53.48, 51.86, 37.61, 36.61, 36.03. HPLC-MS(EI), calculated for C_28_H_31_N_3_O_5_: 490.2 (M+H^+^); found 490.3 (M ^+^).

###
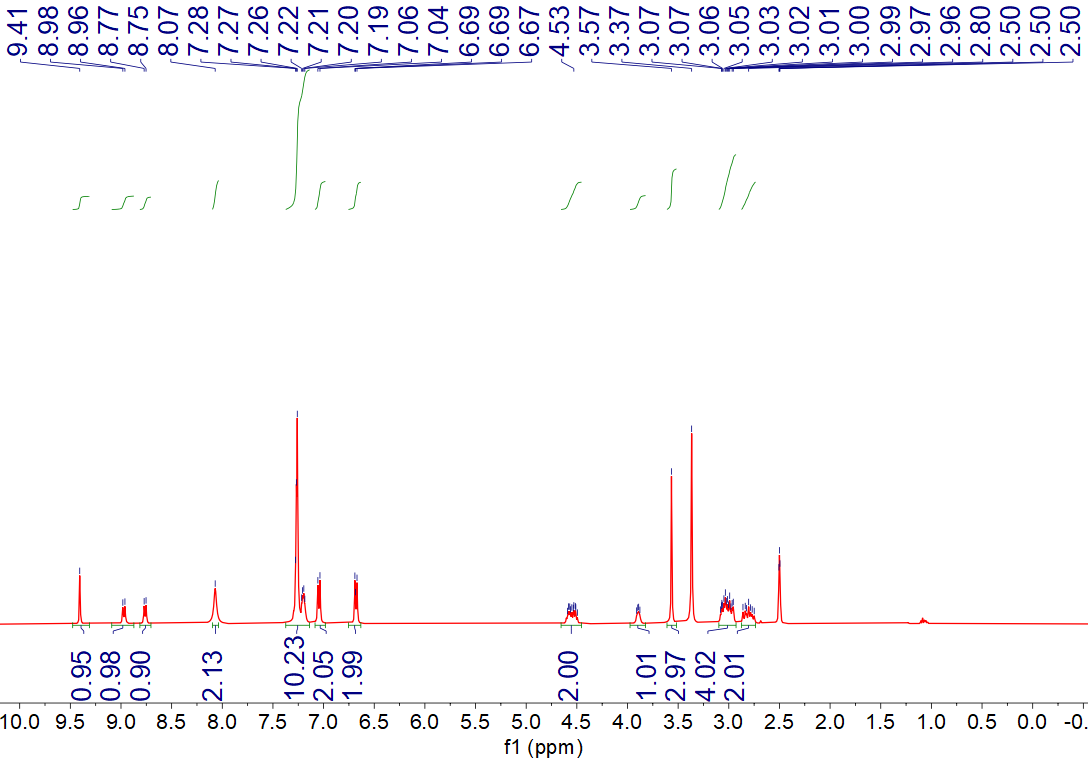


###
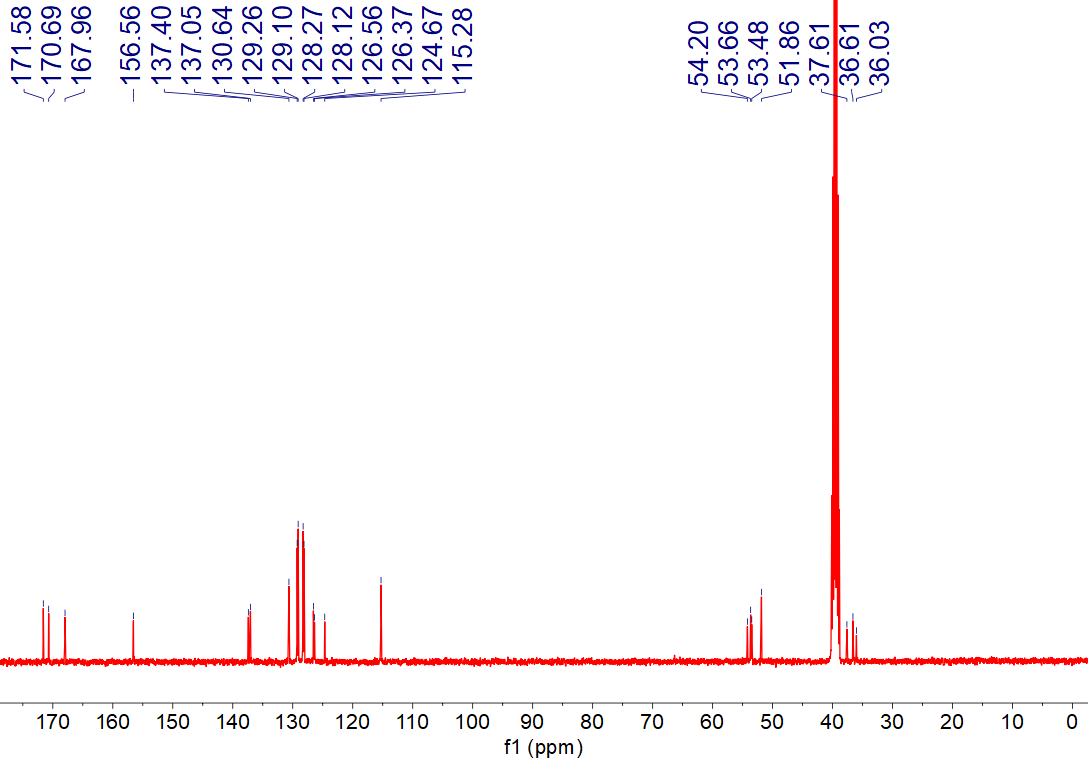


###
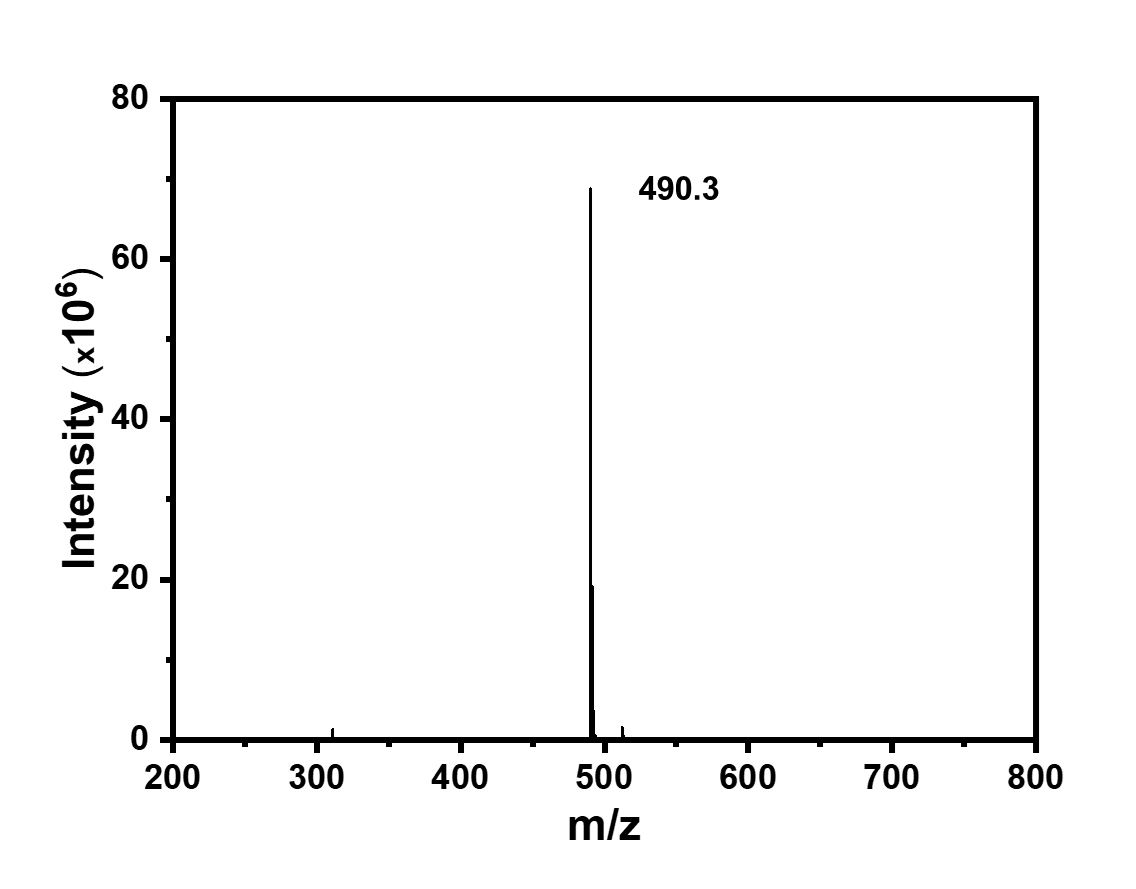


**Figure S8.** ^1^H NMR, ^13^C NMR and LC-MS spectra of YFF (NMRs in DMSO-*d_6_*)

**Synthesis of FXF**

###

**Scheme S2**. Synthesis route of tripeptide FXF. Derivatives of diphenylalanine-based compounds were synthesized via simple multi-step reactions according to literature reports with slight modifications^[1,2]^.

**FMF:** The synthesis of FMF was realized in a four-step reaction as below, as previously reported ^[1]^. The same synthesis steps will be applied to the synthesis of **FGF, FAF, FVF, FIF, FLF, FYF**.

**(i)** First, Boc-*L*-Methionine (Boc-Met-OH) (Boc-Met-OH) (274.3 mg, 1.1 mmol), HBTU (413 mg, 1.1 mmol) and HOBt (147 mg, 1.1 mmol) were dissolved in 1.5 mL DMF in a round-bottom flask and the mixture was stirred with a magnetic stirrer. N, N-diisopropylethylamine (DIPEA) (350 μL, 2.2 mmol), and then *L*-Phenylalanine methyl ester hydrochloride (NH_2_-Phe-OMe) (215 mg, 1 mmol) were added and the reaction mixture was stirred for 24 h at room temperature. The reaction mixture was poured into 50 mL of water. The white precipitate was collected by filtration and washed with water. The white solid was dissolved in DCM, washed twice with 1% HCl and saturated sodium bicarbonate to remove the remaining raw materials and salt, and finally washed with saturated brine, dried over MgSO_4_, and then the solvent was removed under vacuum to obtain the solid product 415 mg (Boc-MF).

**(ii)** The intermediate compound (300 mg) was dissolved in 6 mL of 4 M HCl in dioxane for deprotection. After stirring for 3 hours, the solvent was evaporated on a rotary evaporator to give an oily residue. Diethyl ether was added to the flask and the contents were stirred gently. A white precipitate formed and was separated by centrifugation, yielding about 274 mg of a white product (MF).

**(iii)** Then, N-(tert-butoxycarbonyl)-L-phenylalanine (Boc-Phe-OH) (146 mg, 0.55 mmol), HBTU (206.5 mg, 0.55 mmol) and HOBt (74 mg, 0.55 mmol) were dissolved in 0.75 mL DMF in a round-bottom flask and the mixture was stirred with a magnetic stirrer. N, N-diisopropylethylamine (DIPEA) (175 μL, 1.1mmol), and then L-MF hydrochloride (173.5 mg, 0.5 mmol) were added and the reaction mixture was stirred for 24 h at room temperature. The reaction mixture was poured into 50 mL of water. The white precipitate was collected by filtration and washed with water. The white solid was dissolved in DCM, washed twice with 1% HCl and saturated sodium bicarbonate to remove the remaining raw materials and salt, and finally washed with saturated brine, dried over MgSO_4_, and then the solvent was removed in vacuum to obtain the solid product 214 mg (Boc-FMF).

**(iv)** The intermediate compound (214 mg) was dissolved in 6 mL of 4 M hydrogen chloride solution in dioxane for deprotection. After stirring for 3 hours, the solvent was evaporated on a rotary evaporator to give an oily residue. Diethyl ether was added to the flask and the contents were stirred gently. A white precipitate formed and was separated by centrifugation, yielding about 180 mg of a white product (FMF). The product was characterized by NMR.^1^H NMR (400 MHz, DMSO-*d_6_*): *δ* 8.82 (s, 1H), 8.60 (dd, *J* = 8.1, 2.9 Hz, 1H), 8.23 (s, 2H), 7.39 – 7.07 (m, 10H), 4.56 – 4.38 (m, 2H), 4.09 (s, 1H), 3.60 (s, 3H), 3.17 – 2.84 (m, 4H), 2.48 – 2.32 (m, 2H), 2.05 (s, 3H), 1.95 – 1.71 (m, 2H). HPLC-MS(EI), calculated for C_24_H_31_N_3_O_4_S:458.2 (M+H^+^); found 458.3 (M ^+^).

###
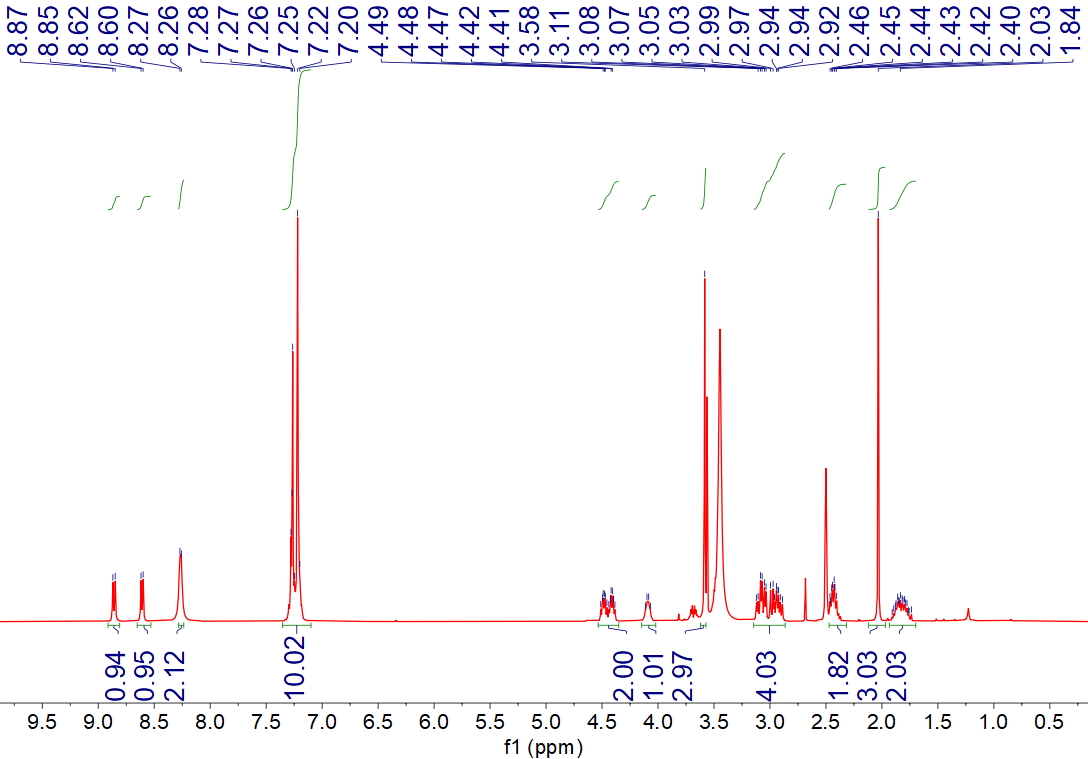


###
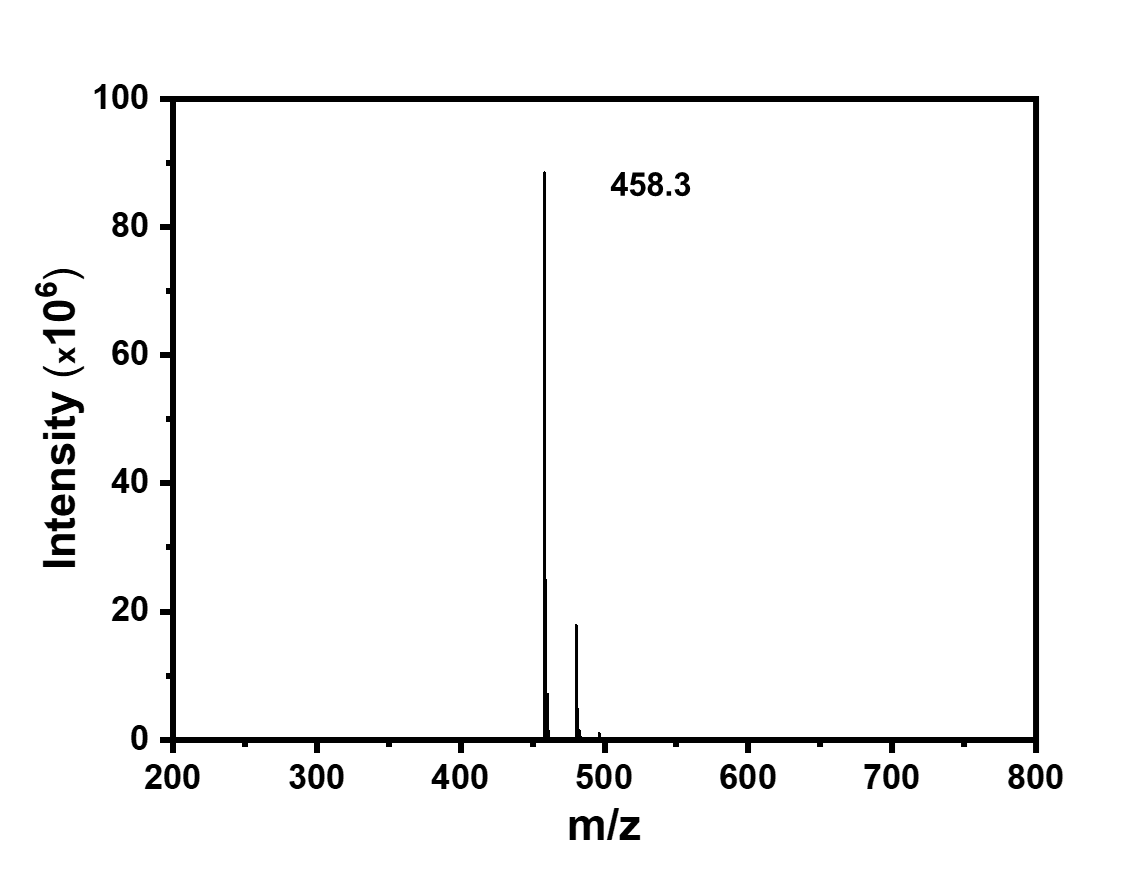


**Figure S9.** ^1^H NMR and LC-MS spectra of FMF (NMRs in DMSO-*d_6_*)

**FGF:** ^1^H NMR (400 MHz, DMSO-*d_6_*): *δ* 8.89 (t, *J* = 5.6 Hz, 1H), 8.61 (d, *J* = 7.6 Hz, 1H), 8.39 (s, 2H), 7.36 – 7.14 (m, 10H), 4.49 (td, *J* = 8.2, 5.9 Hz, 1H), 4.10 (q, *J* = 6.7, 5.8 Hz, 1H), 3.82 (dd, *J* = 16.9, 5.7 Hz, 1H), 3.69 (dd, *J* = 16.8, 5.5 Hz, 1H), 3.59 (s, 3H), 3.18 – 2.93 (m, 4H).^13^C NMR (101 MHz, DMSO-*d_6_*): *δ* 171.77, 168.25, 168.17, 137.14, 134.97, 129.58, 129.11, 128.43, 128.26, 127.05, 126.57, 53.73, 53.33, 51.87, 41.57, 36.74, 36.67. HPLC-MS(EI), calculated for C_21_H_25_N_3_O_4_:384.2 (M+H^+^); found 384.2 (M ^+^).

###
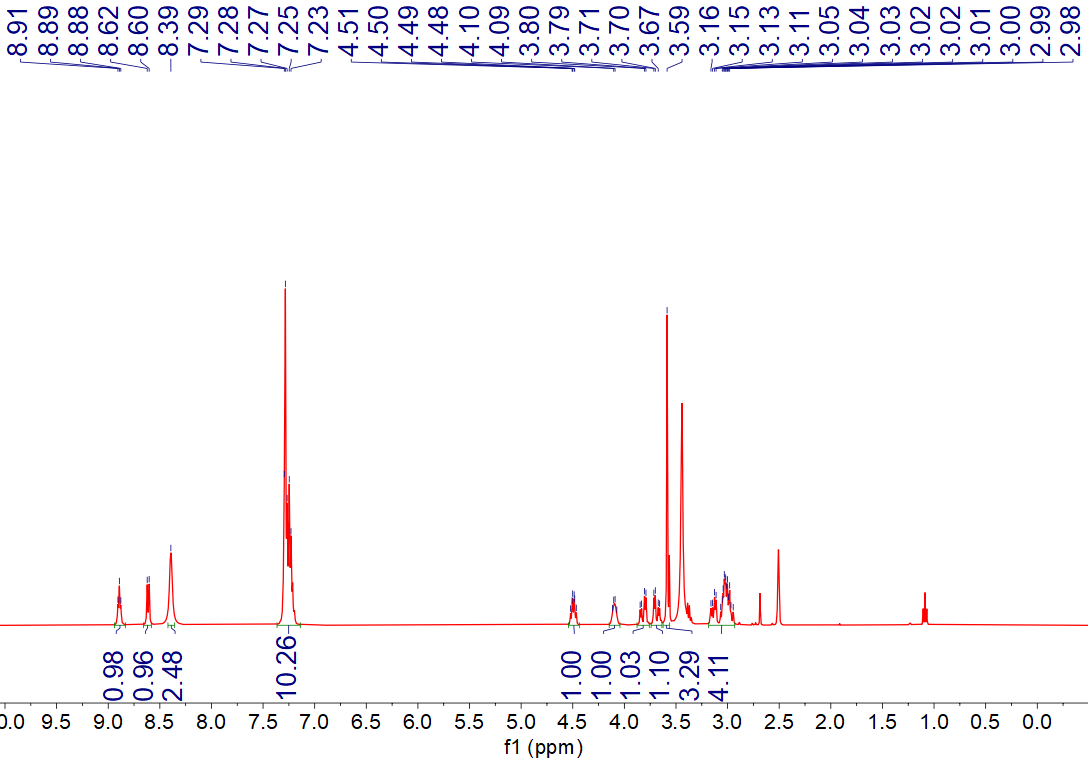


###
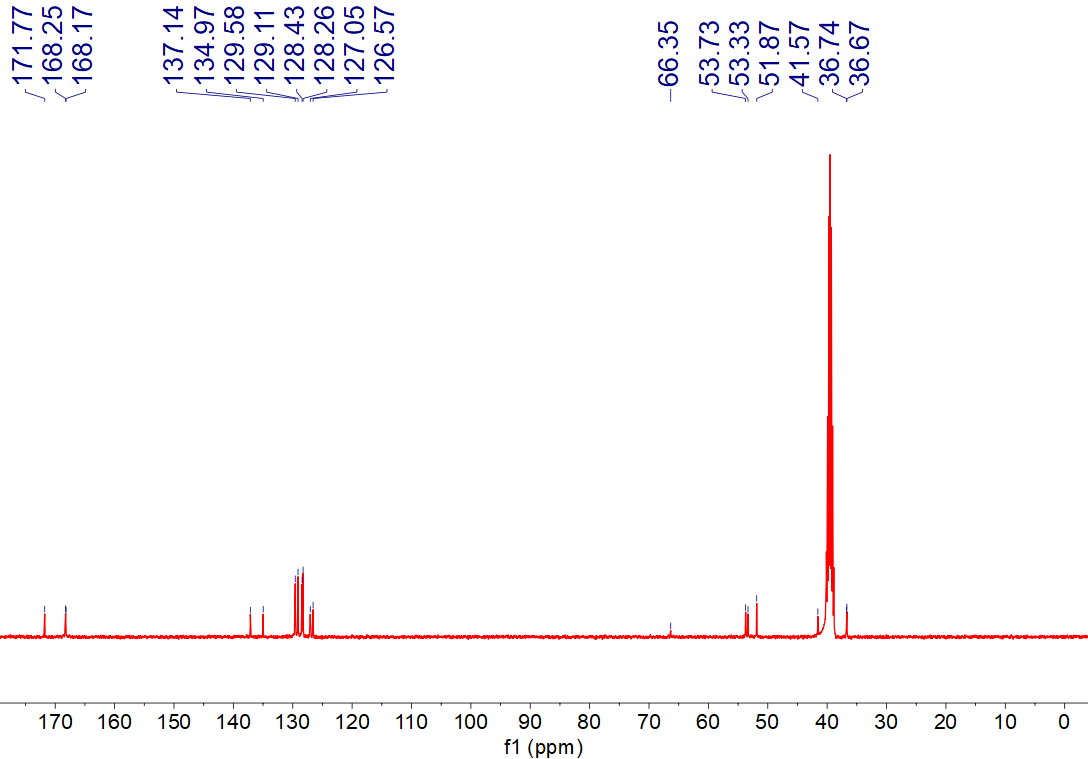


###
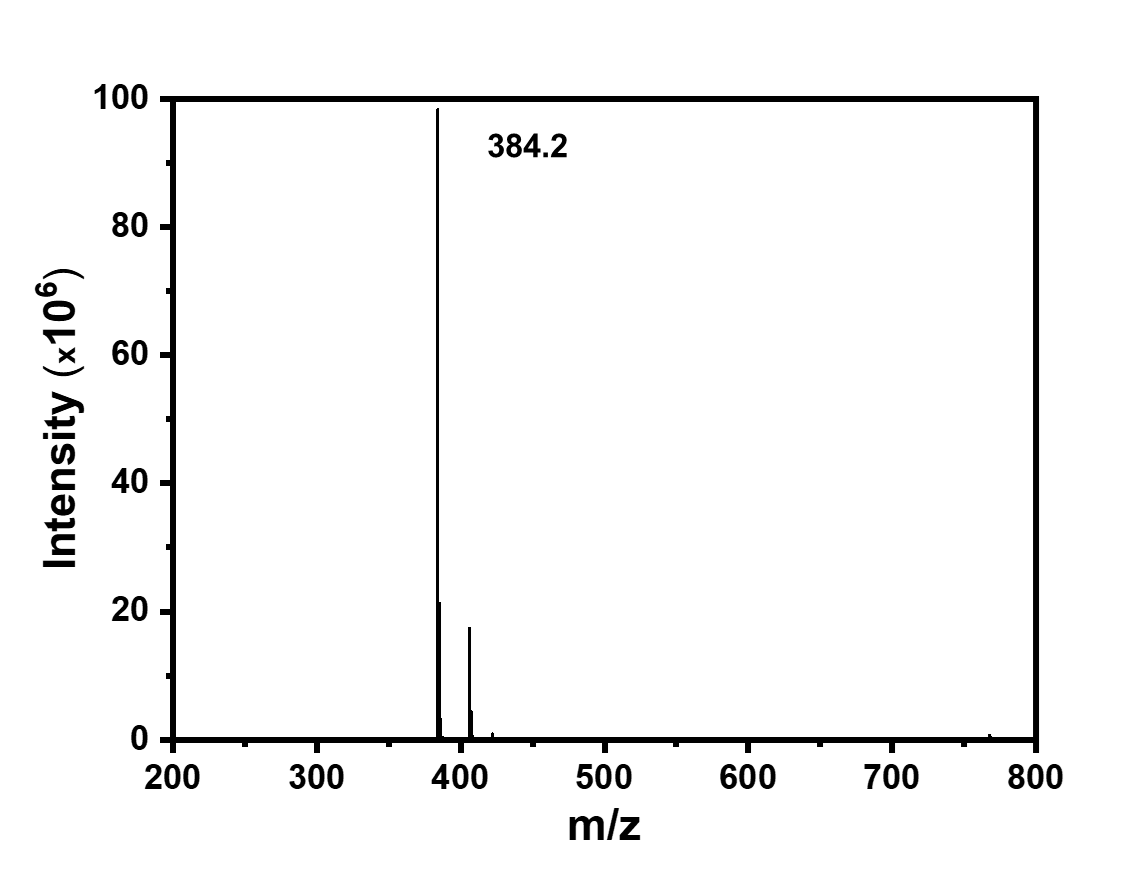


**Figure S10.** ^1^H NMR, ^13^C NMR and LC-MS spectra of FGF (NMRs in DMSO-*d_6_*)

**FAF:** ^1^H NMR (400 MHz, DMSO-*d_6_*): *δ* 8.85 (d, *J* = 7.6 Hz, 1H), 8.54 (d, *J* = 7.5 Hz, 1H), 8.28 (d, *J* = 5.1 Hz, 2H), 7.36 – 7.13 (m, 10H), 4.48 (td, *J* = 8.4, 5.9 Hz, 1H), 4.37 (t, J = 7.2 Hz, 1H), 4.07 (q, J = 6.0 Hz, 1H), 3.59 (s, 3H), 3.18 – 2.85 (m, 4H), 1.22 (d, *J* = 7.0 Hz, 3H). ^13^C NMR (101 MHz, DMSO-*d_6_*): *δ* 171.79, 171.71, 167.44, 164.60, 137.15, 134.97, 129.61, 129.10, 128.37, 128.24, 126.98, 126.55, 72.16, 60.16, 53.66, 53.18, 51.86, 48.13, 43.61, 38.23, 36.70, 36.48, 18.41. HPLC-MS(EI), calculated for C_22_H_27_N_3_O_4_:398.2 (M+H^+^): found 398.3 (M ^+^).

###
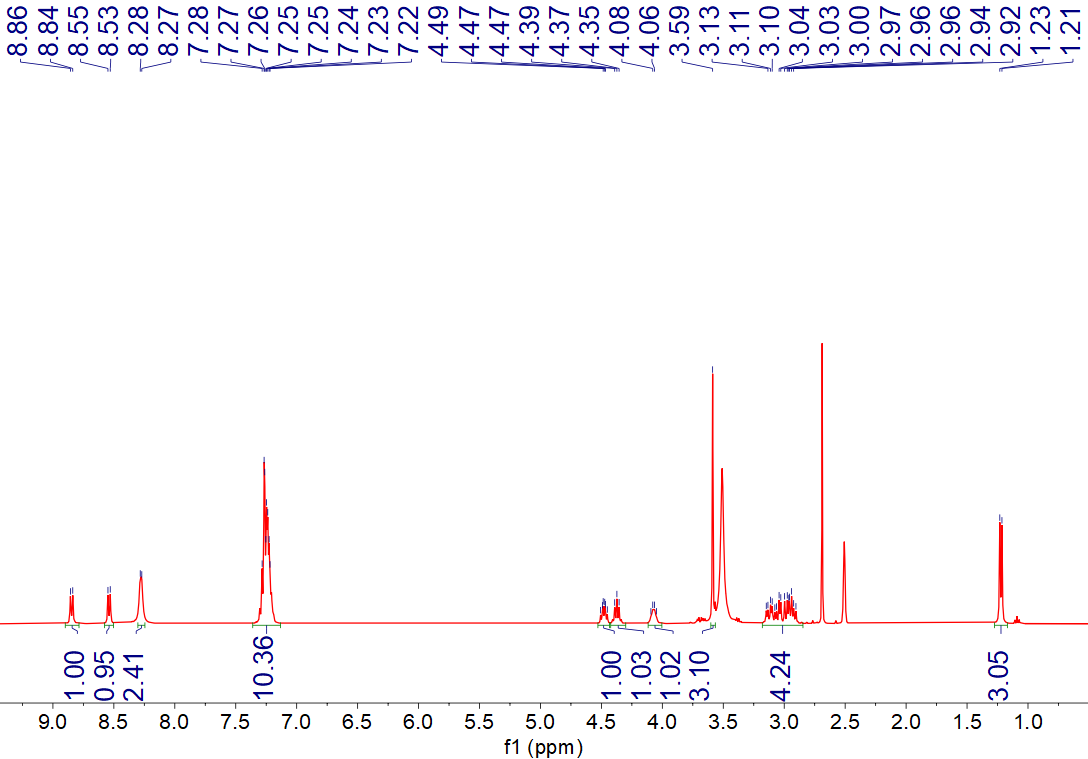


###
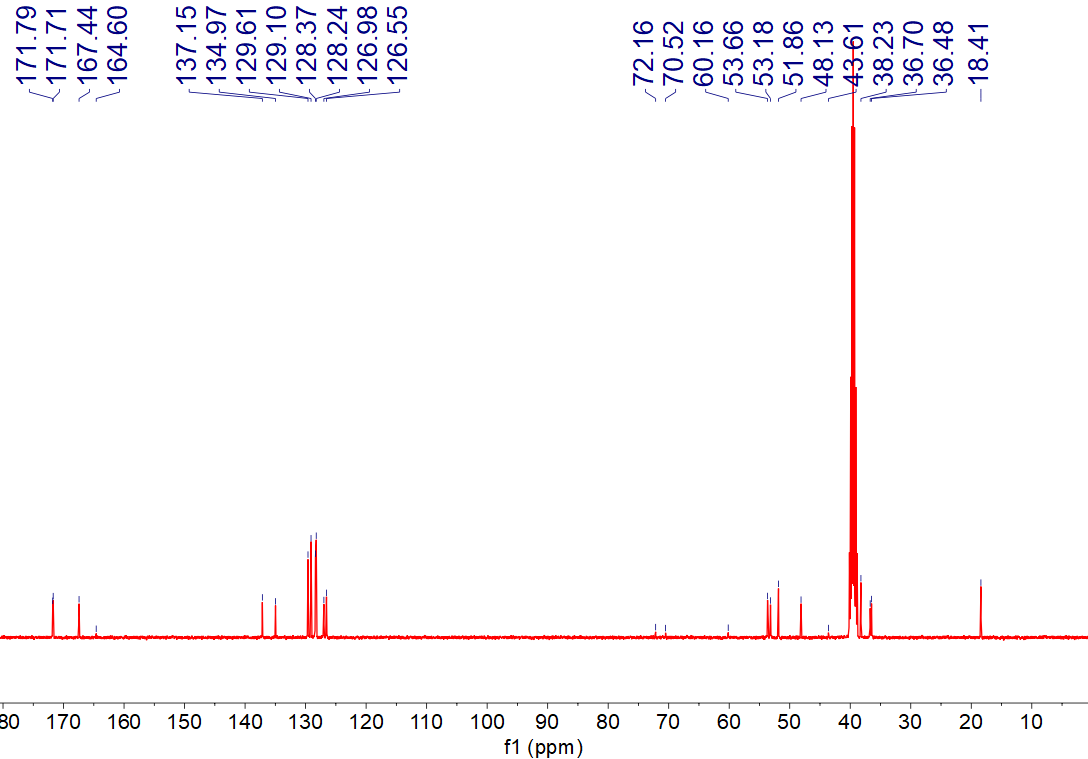


###
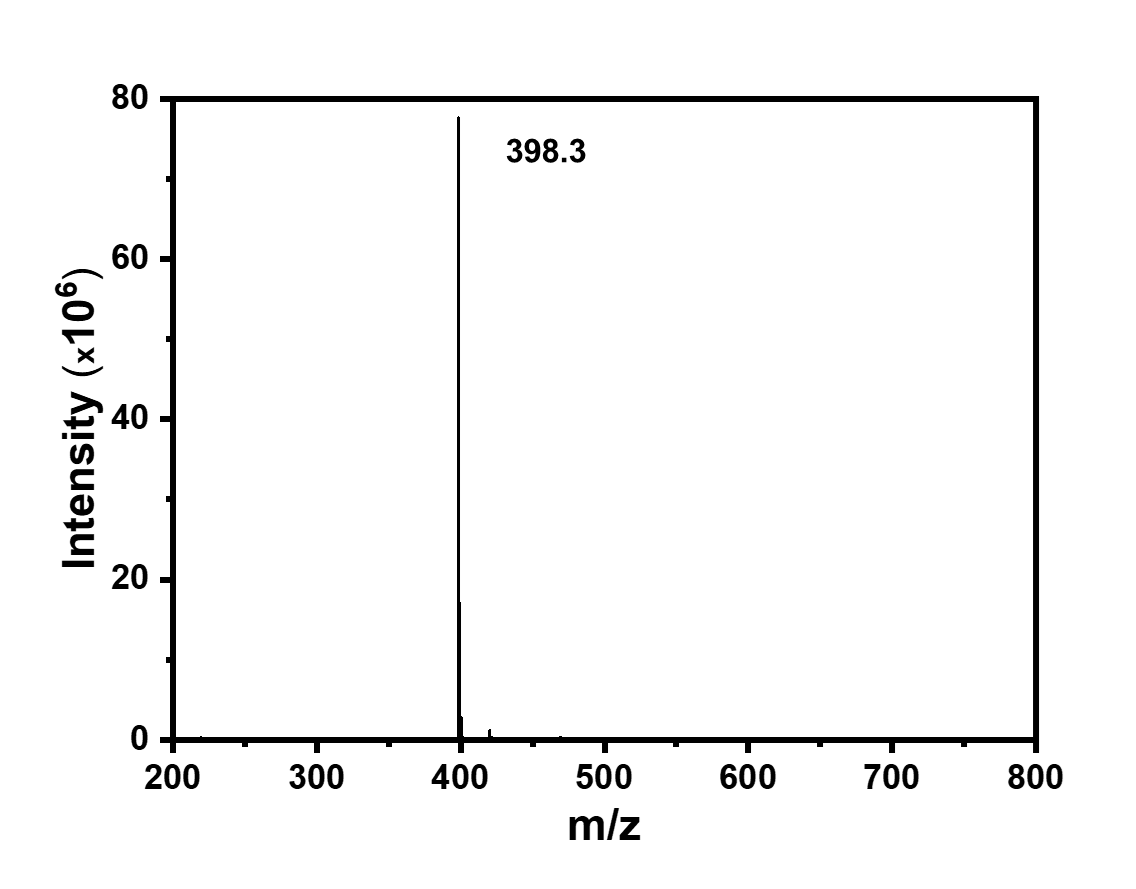


**Figure S11.** ^1^H NMR, ^13^C NMR and LC-MS spectra of FAF (NMRs in DMSO-*d_6_*)

**FVF:** ^1^H NMR (400 MHz, DMSO-*d_6_*): *δ* 8.61 (d, *J* = 7.6 Hz, 2H), 8.19 (s, 2H), 7.49 – 7.06 (m, 10H), 4.49 (d, *J* = 7.4 Hz, 1H), 4.23 (td, *J* = 6.8, 3.7 Hz1 1H), 4.12 (s, 1H), 3.57 (d, *J* = 1.7 Hz, 3H), 3.12 – 2.80 (m, 4H), 1.95 (q, *J* = 6.8 Hz, 1H), 0.86 (d, *J* = 6.6 Hz, 6H).^13^C NMR (101 MHz, DMSO-*d_6_*): *δ* 171.73, 170.51, 167.70, 137.19, 134.89, 129.57, 129.01, 128.33, 128.23, 126.93, 126.52, 72.15, 70.51, 66.34, 60.16, 57.72, 53.65, 53.09, 51.73, 36.81, 36.46, 30.92, 18.98, 18.24. HPLC-MS(EI), calculated for C_24_H_31_N_3_O_4_:426.2 (M+H^+^): found 426.3 (M ^+^).

###
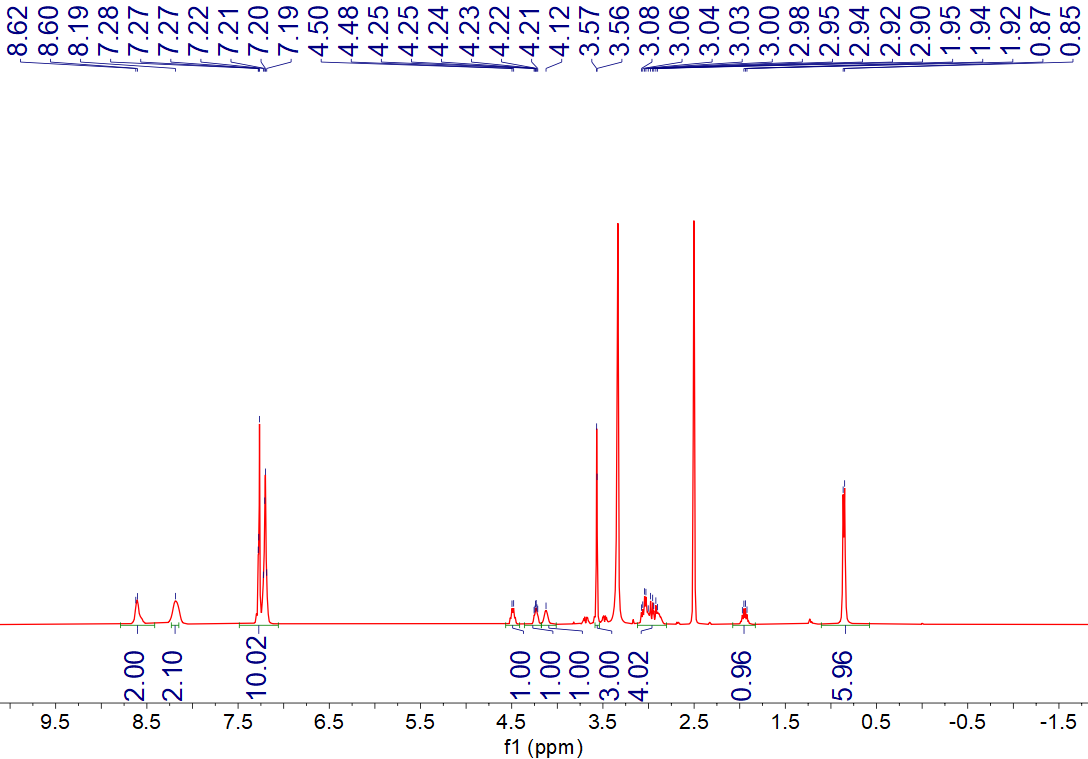


###
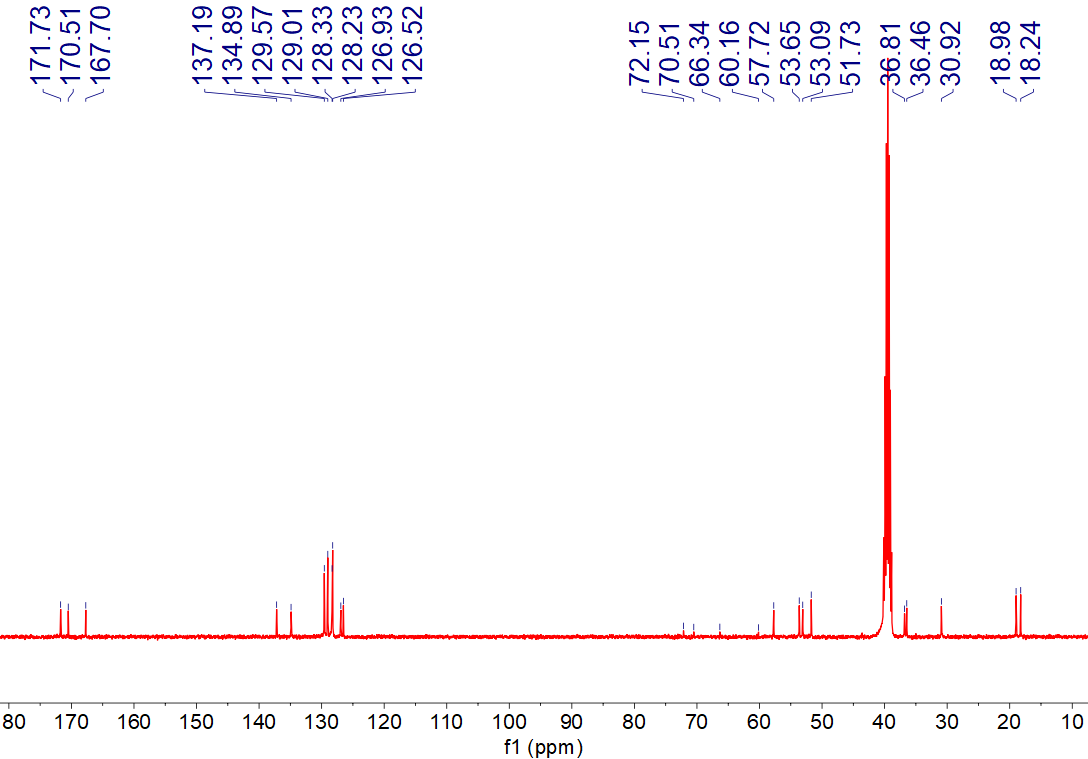


###
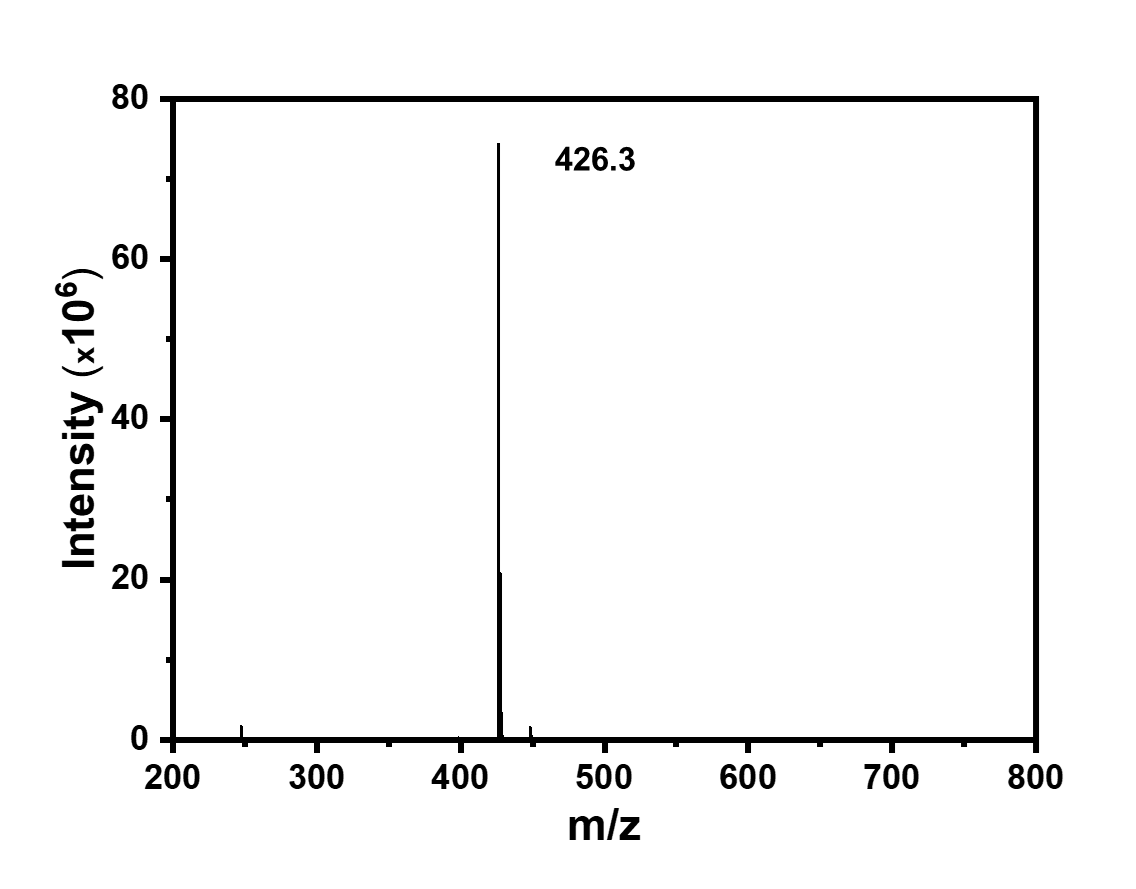


**Figure S12.** ^1^H NMR, ^13^C NMR and LC-MS spectra of FVF (NMRs in DMSO-*d_6_*)

**FLF:** ^1^H NMR (400 MHz, DMSO-*d_6_*): *δ* 8.86 (d, *J* = 8.4 Hz, 1H), 8.62 (d, *J* = 7.5 Hz, 1H), 8.27 – 8.20 (m, 2H), 7.36 – 7.10 (m, 10H), 4.55 – 4.45 (m, 1H), 4.42 – 4.31 (m, 1H), 4.05 (s, 1H), 3.56 (s, 3H), 3.15 – 2.86 (m, 4H), 1.59 (dt, *J* = 13.4, 6.6 Hz, 1H), 1.50 – 1.33 (m, 2H), 0.86 (dd, *J* = 15.7, 6.5 Hz, 6H).^13^C NMR (101 MHz, DMSO-*d_6_*): *δ* 171.70, 171.53, 167.55, 137.20, 134.92, 129.63, 129.04, 128.37, 128.22, 126.97, 126.49, 66.34, 53.57, 53.20, 51.80, 51.11, 41.21, 36.72, 36.41, 23.90, 22.89, 21.85. HPLC-MS(EI), calculated for C_25_H_33_N_3_O_4_: 440.3 (M+H^+^): found 440.3 (M ^+^).

###
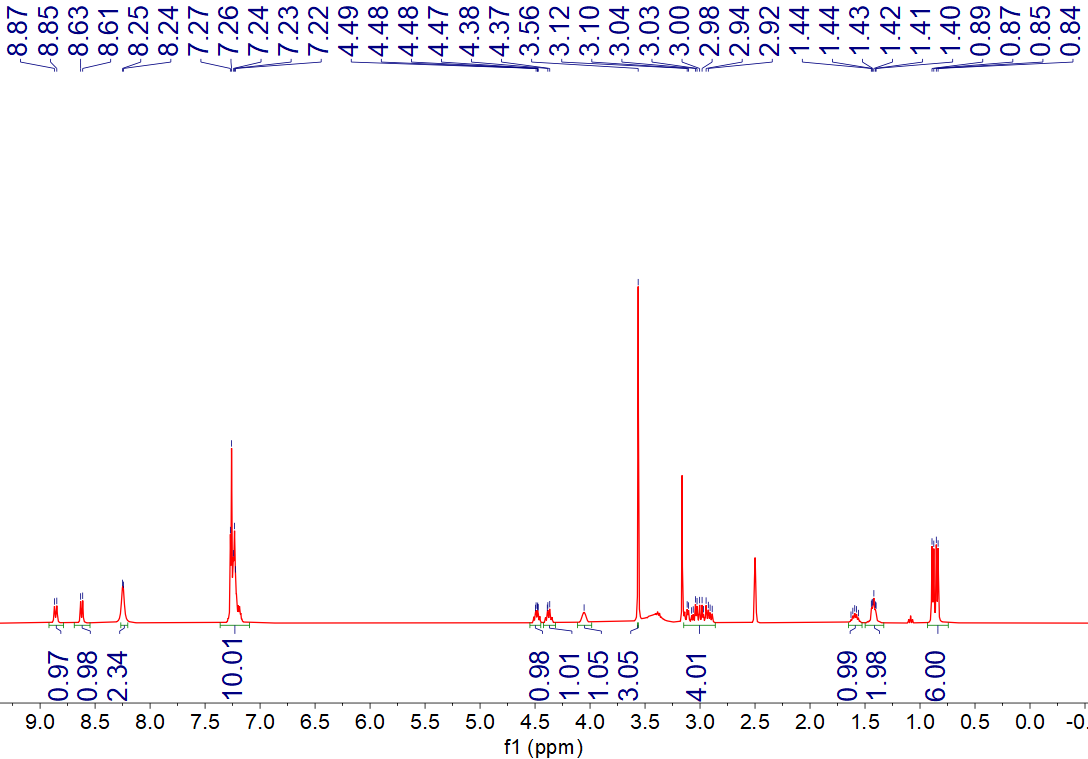


###
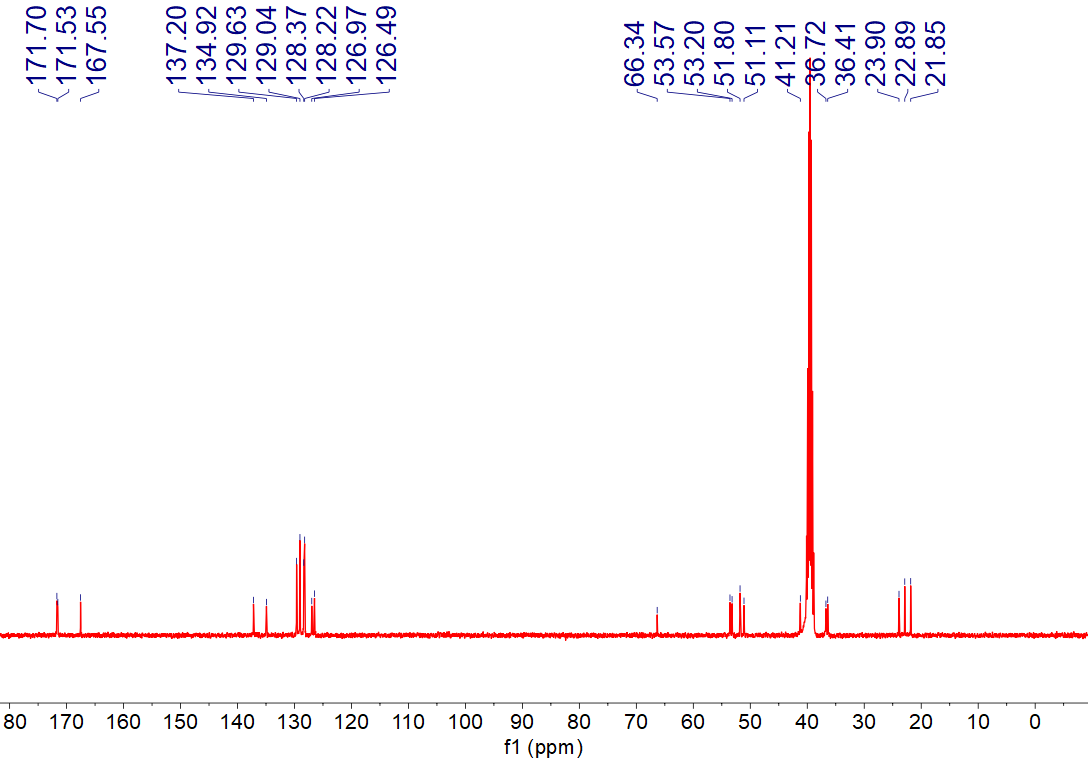


###
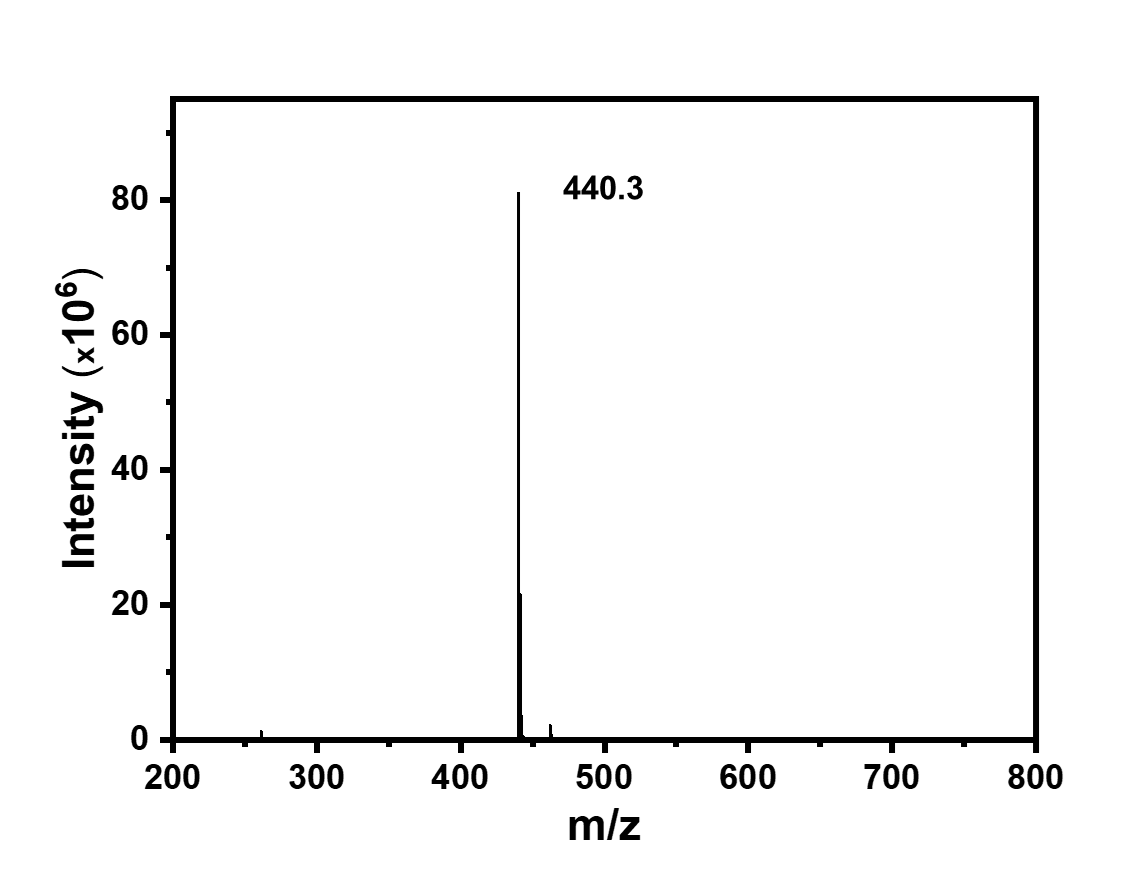


**Figure S13.** ^1^H NMR, ^13^C NMR and LC-MS spectra of FLF (NMRs in DMSO-*d_6_*)

**FIF:** ^1^H NMR (400 MHz, DMSO-*d_6_*): *δ* 8.75 (d, *J* = 9.0 Hz, 1H), 8.68 (d, *J* = 7.3 Hz, 1H), 8.29 (s, 2H), 7.33 – 7.16 (m, 10H), 4.49 (m, *J* = 8.9, 6.6 Hz, 1H), 4.24 (t, *J* = 8.3 Hz, 1H), 4.18 – 4.07 (m, 1H), 3.56 (s, 3H), 3.14 – 2.86 (m, 4H), 1.78 – 1.62 (m, 1H), 1.46 (m, *J* = 13.5, 7.5, 3.3 Hz, 1H), 1.08 (m, *J* = 13.2, 8.5, 7.9, 4.4 Hz, 1H), 0.90 – 0.71 (m, 6H).^13^C NMR (101 MHz, DMSO-*d_6_*): *δ* 171.68, 170.55, 167.63, 137.20, 134.90, 129.58, 129.01, 128.33, 128.22, 126.93, 126.50, 56.89, 53.62, 53.11, 51.72, 37.05, 36.82, 36.45, 24.19, 14.98, 11.04. HPLC-MS(EI), calculated for C_25_H_33_N_3_O_4_:440.3 (M+H^+^); found 440.4 (M ^+^).

###
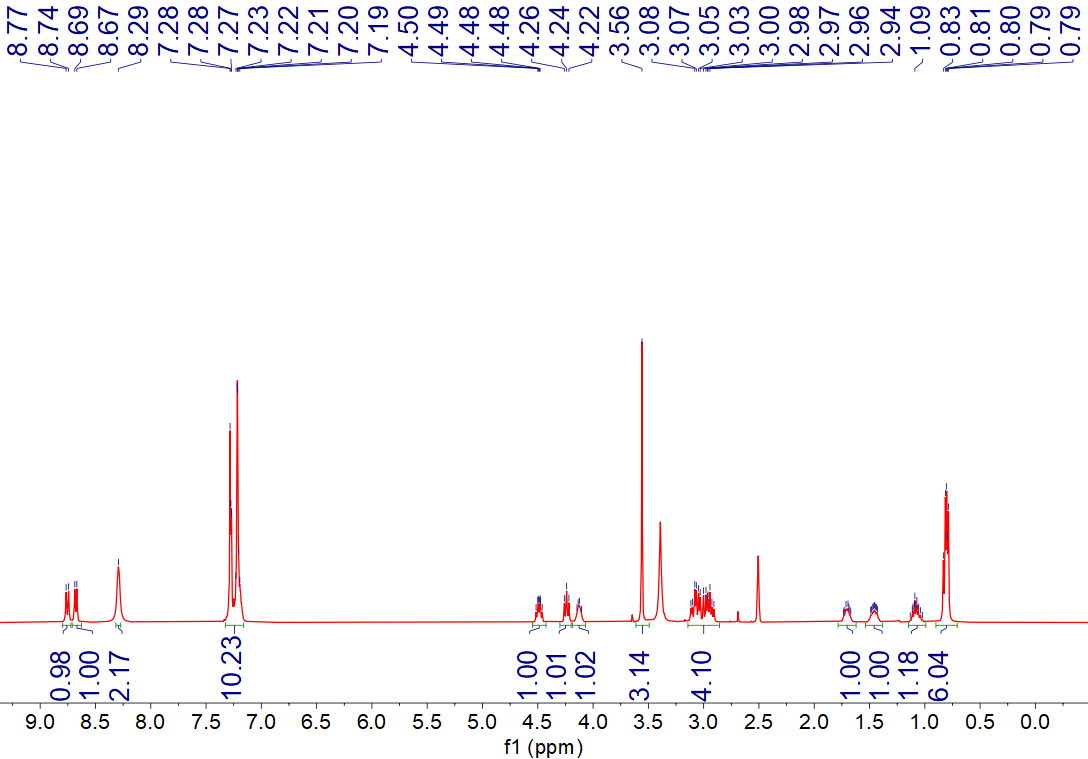


###
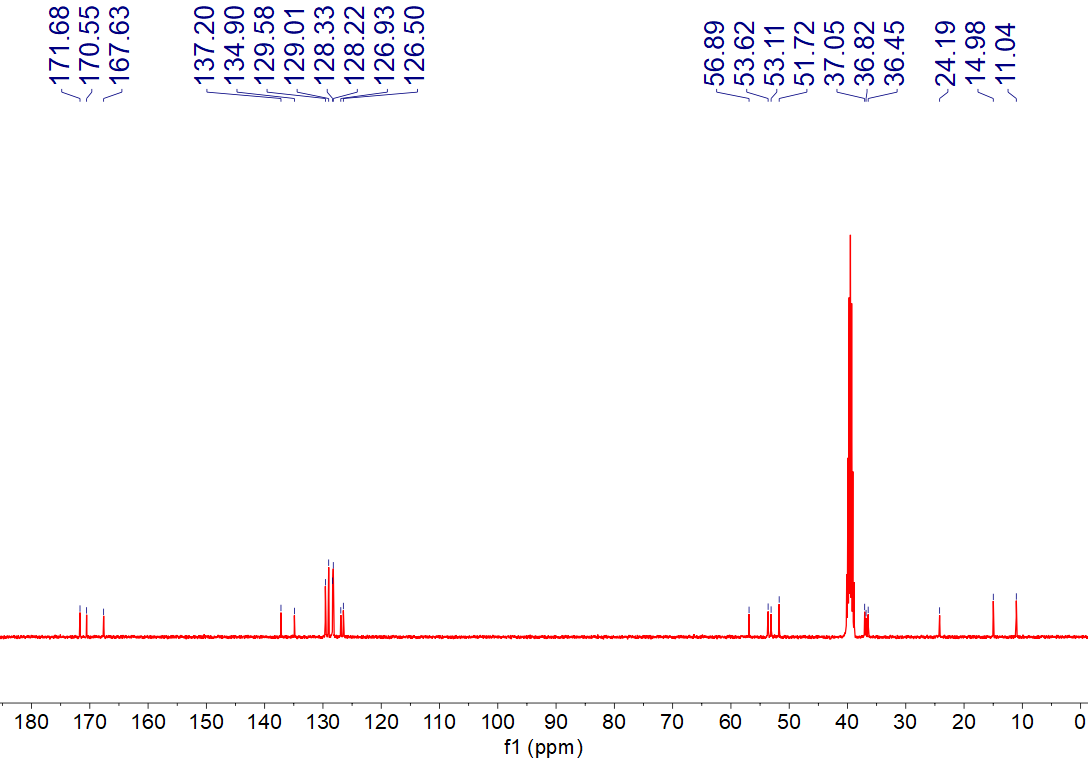


###
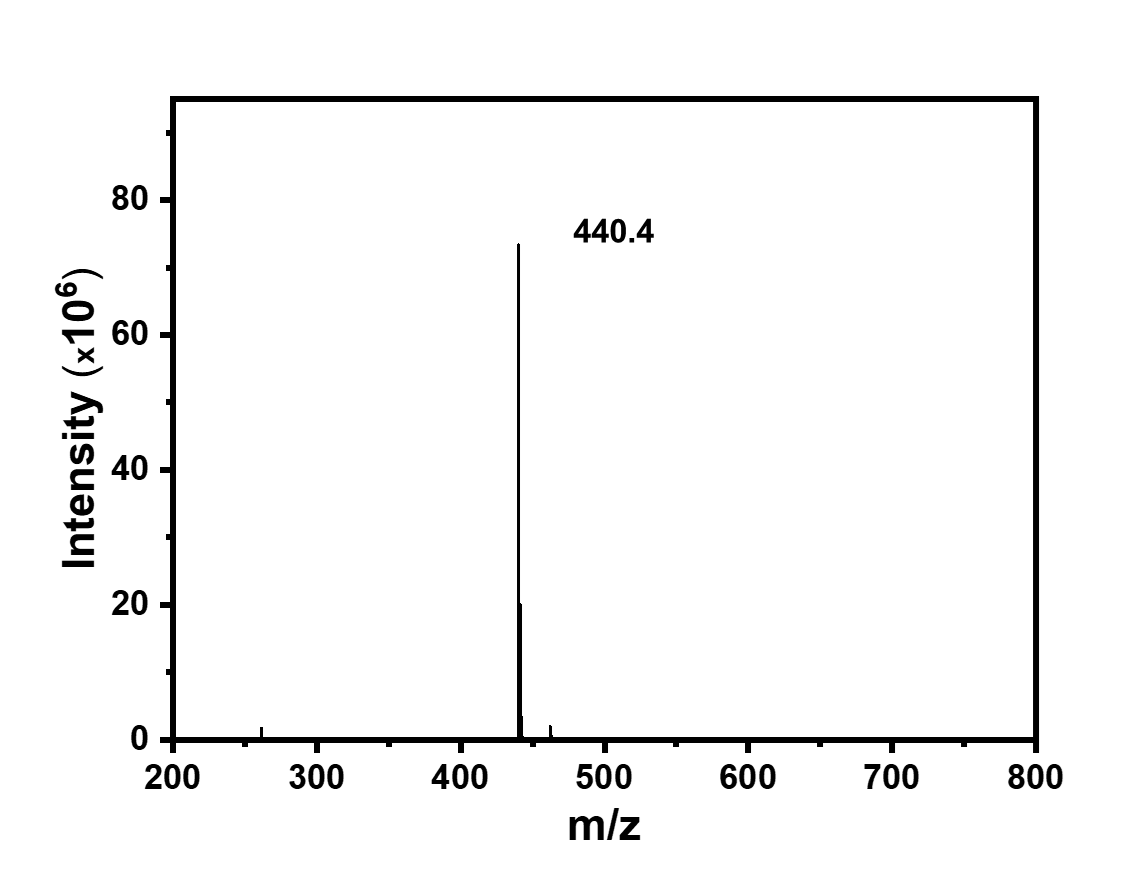


**Figure S14.** ^1^H NMR, ^13^C NMR and LC-MS spectra of FIF (NMRs in DMSO-*d_6_*)

**FYF:** ^1^H NMR (400 MHz, DMSO-*d_6_*): *δ* 9.25 (s, 1H), 8.83 (s, 1H), 8.68 (dd, *J* = 7.7, 3.2 Hz, 1H), 8.09 (s, 2H), 7.25 (d, *J* = 3.8 Hz, 10H), 7.09 – 6.96 (m, 2H), 6.75 – 6.61 (m, 2H), 4.52 (m, *J* = 8.2, 5.7 Hz, 2H), 4.08 – 3.94 (m, 1H), 3.58 (s, 3H), 3.05 – 2.82 (m, 4H), 2.71 (d, *J* = 16.7 Hz, 2H).^13^C NMR (101 MHz, DMSO-*d_6_*): *δ* 172.09, 171.32, 168.18, 156.44, 137.60, 135.32, 130.62, 130.17, 130.00, 129.57, 128.85, 128.74, 127.81, 127.47, 127.01, 115.44, 65.38, 55.05, 54.17, 53.69, 52.33, 37.35, 37.22, 37.07. HPLC-MS(EI), calculated for C_28_H_31_N_3_O_5_: 490.2 (M+H^+^); found 490.3 (M ^+^).

###
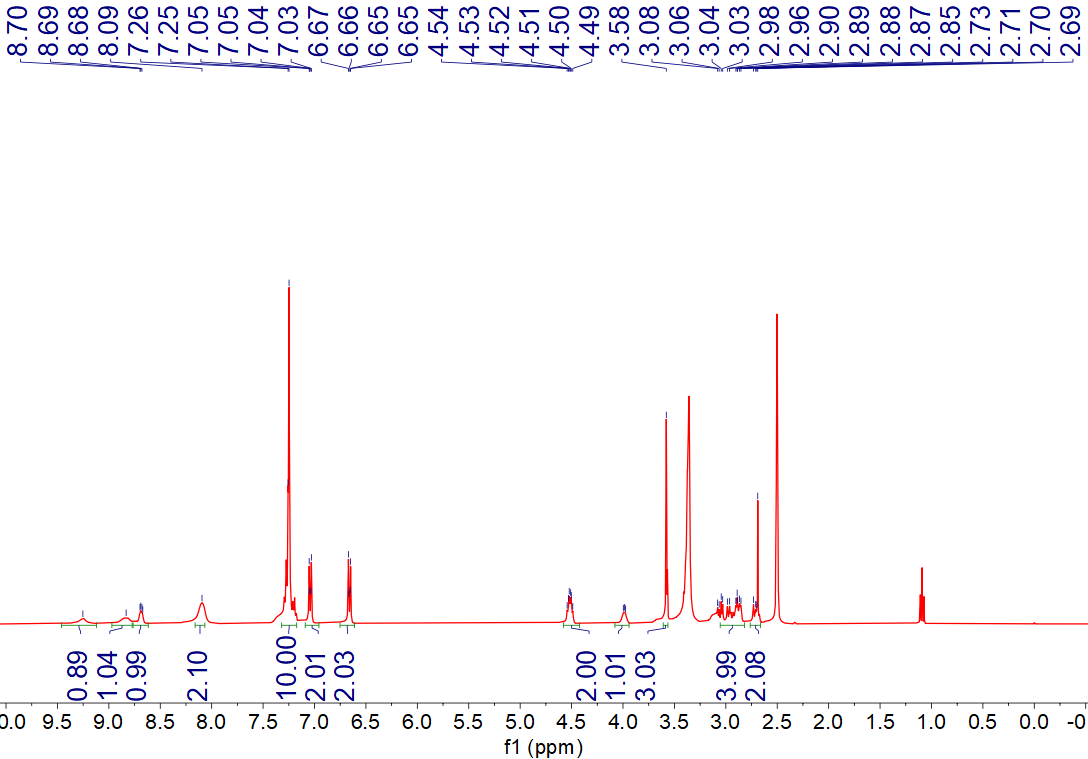


###
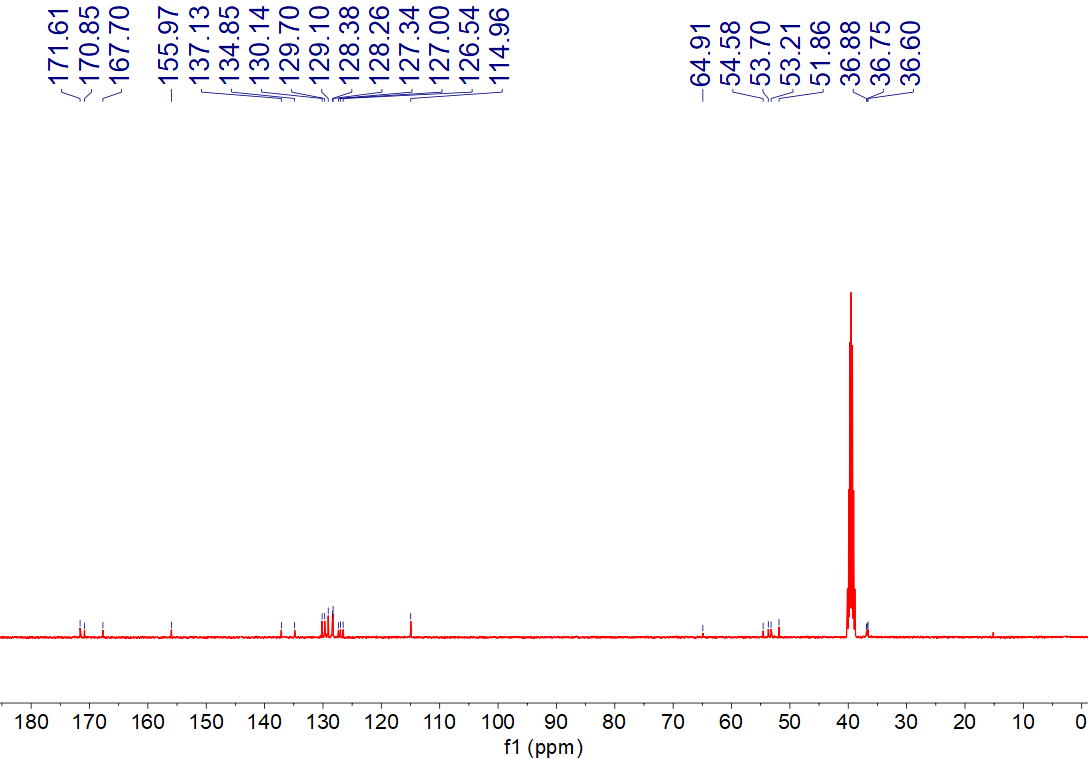


###
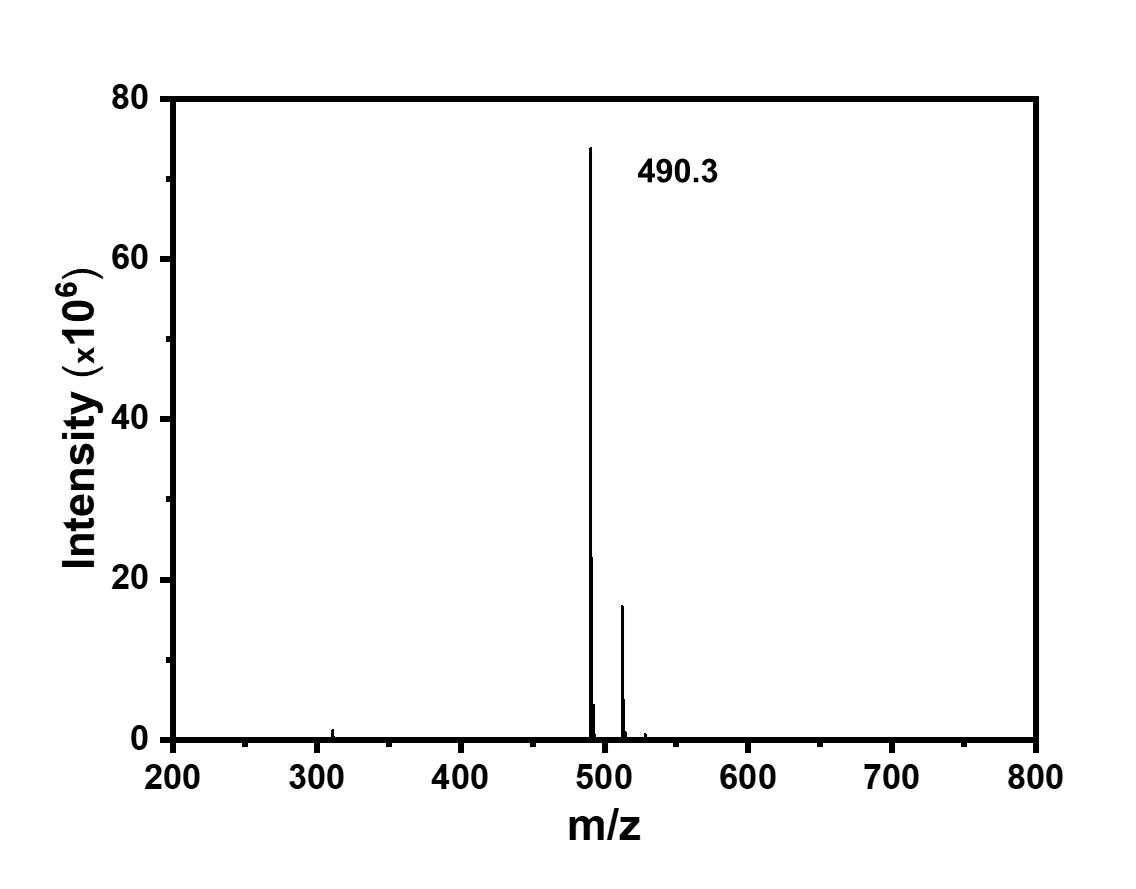


**Figure S15.** ^1^H NMR, ^13^C NMR and LC-MS spectra of FYF (NMRs in DMSO-*d_6_*)

**Synthesis of FFX**

###

**Scheme S3**. Synthesis route of tripeptide FFX. Derivatives of diphenylalanine-based compounds were synthesized via simple multi-step reactions according to literature reports with slight modifications^[1,2]^.

### **FFM**: Similarly, this compound was synthesized in a four-step reaction as previously reported ^[1,2]^. The same synthesis steps will be applied to the synthesis of **FFG, FFA, FFV, FFI, FFL, and FFY.**

**(i)** First, N-(tert-butoxycarbonyl)-L-phenylalanine (Boc-Phe-OH) (291 mg, 1.1 mmol), HBTU (413 mg, 1.1 mmol), and HOBt (147 mg, 1.1 mmol) were dissolved in 1.5 mL DMF in a round-bottom flask and the mixture was stirred with a magnetic stirrer. N, N-diisopropylethylamine (DIPEA) (350 μL, 2.2 mmol), and then *L*-Methionine methyl ester hydrochloride (NH_2_-Met-OMe) (199 mg, 1 mmol) were added and the reaction mixture was stirred for 24 h at room temperature. The reaction mixture was poured into 50 mL of water. The white precipitate was collected by filtration and washed with water. The white solid was dissolved in DCM, washed twice with 1% HCl and saturated sodium bicarbonate to remove the remaining raw materials and salt, and finally washed with saturated brine, dried over MgSO_4_, and then the solvent was removed in vacuum to obtain the solid product 458 mg (Boc-FM).

**(ii)** The intermediate compound (300 mg) was dissolved in 6 mL of 4 M hydrogen chloride solution in dioxane for deprotection. After stirring for 3 hours, the solvent was evaporated on a rotary evaporator to give an oily residue. Diethyl ether was added to the flask, and the contents were stirred gently. A white precipitate formed and was separated by centrifugation, yielding about 271 mg of a white product (FM).

**(iii)** Then, N-(tert-butoxycarbonyl)-L-phenylalanine (Boc-Phe-OH) (146 mg, 0.55 mmol), HBTU (206.5 mg, 0.55 mmol) and HOBt (74 mg, 0.55 mmol) were dissolved in 0.75 mL DMF in a round-bottom flask and the mixture was stirred with a magnetic stirrer. N, N-diisopropylethylamine (DIPEA) (175 μL, 1.1mmol), and then FM-OMe hydrochloride (173 mg, 0.5 mmol) were added and the reaction mixture was stirred for 24 h at room temperature. The reaction mixture was poured into 50 mL of water. The white precipitate was collected by filtration and washed with water. The white solid was dissolved in DCM, washed twice with 1% HCl and saturated sodium bicarbonate to remove the remaining raw materials and salt, and finally washed with saturated brine, dried over MgSO_4_, and then the solvent was removed under vacuum to obtain the solid product 226 mg (Boc-FFM).

**(iv)** The intermediate compound (180 mg) was dissolved in 4 mL of 4 M hydrogen chloride solution in dioxane for deprotection. After stirring for 3 hours, the solvent was evaporated on a rotary evaporator to give an oily residue. Diethyl ether was added to the flask, and the contents were stirred gently. A white precipitate formed and was separated by centrifugation, yielding about 147 mg of a white product (FFM). The product was characterized by NMR.^1^H NMR (400 MHz, DMSO-*d_6_*): *δ* 8.92 (d, *J* = 8.1 Hz, 1H), 8.66 (d, *J* = 7.7 Hz, 1H), 8.10 (d, *J* = 5.3 Hz, 2H), 7.31 – 7.20 (m, 10H), 4.61 (d, *J* = 5.0 Hz, 1H), 4.43 (m, *J* = 9.2, 7.6, 4.6 Hz, 1H), 4.03 – 3.96 (m, 1H), 3.62 (s, 3H), 3.14 (dd, *J* = 14.2, 4.8 Hz, 1H), 3.05 (dd, *J* = 14.0, 5.1 Hz, 1H), 2.95 – 2.82 (m, 4H), 2.04 (s, 3H), 2.00 – 1.83 (m, 2H). HPLC-MS(EI), calculated for C_24_H_31_N_3_O_4_S:458.2 (M+H^+^); found 458.3 (M ^+^).

###
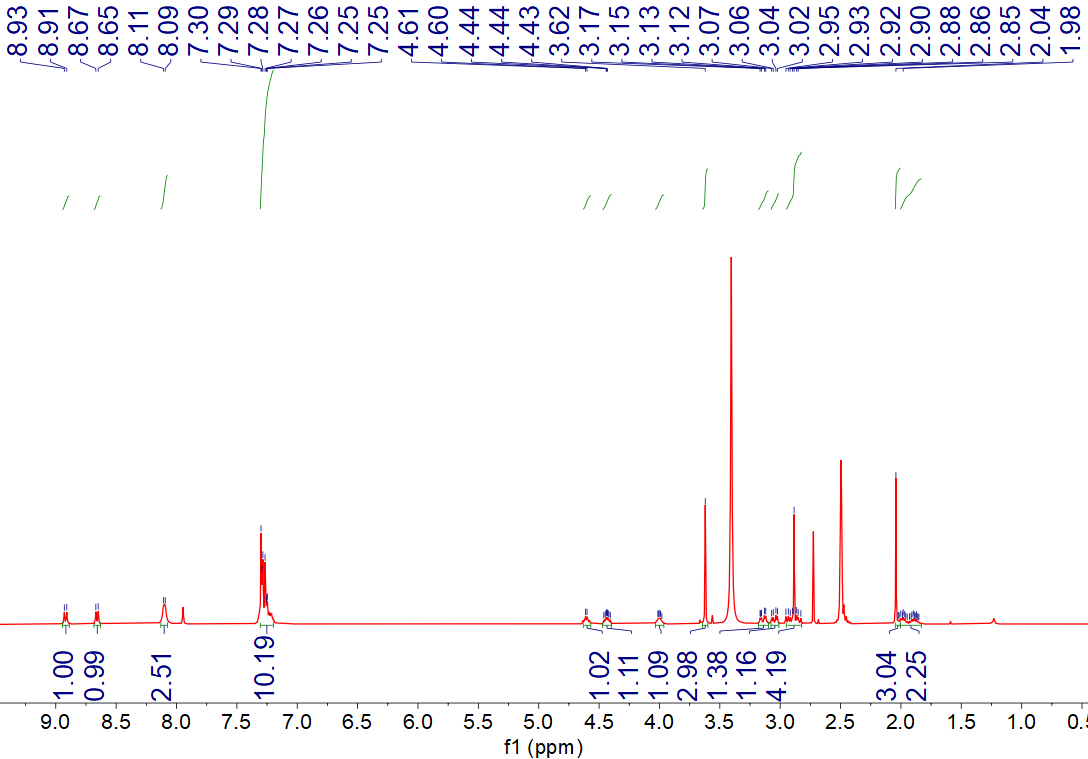


###
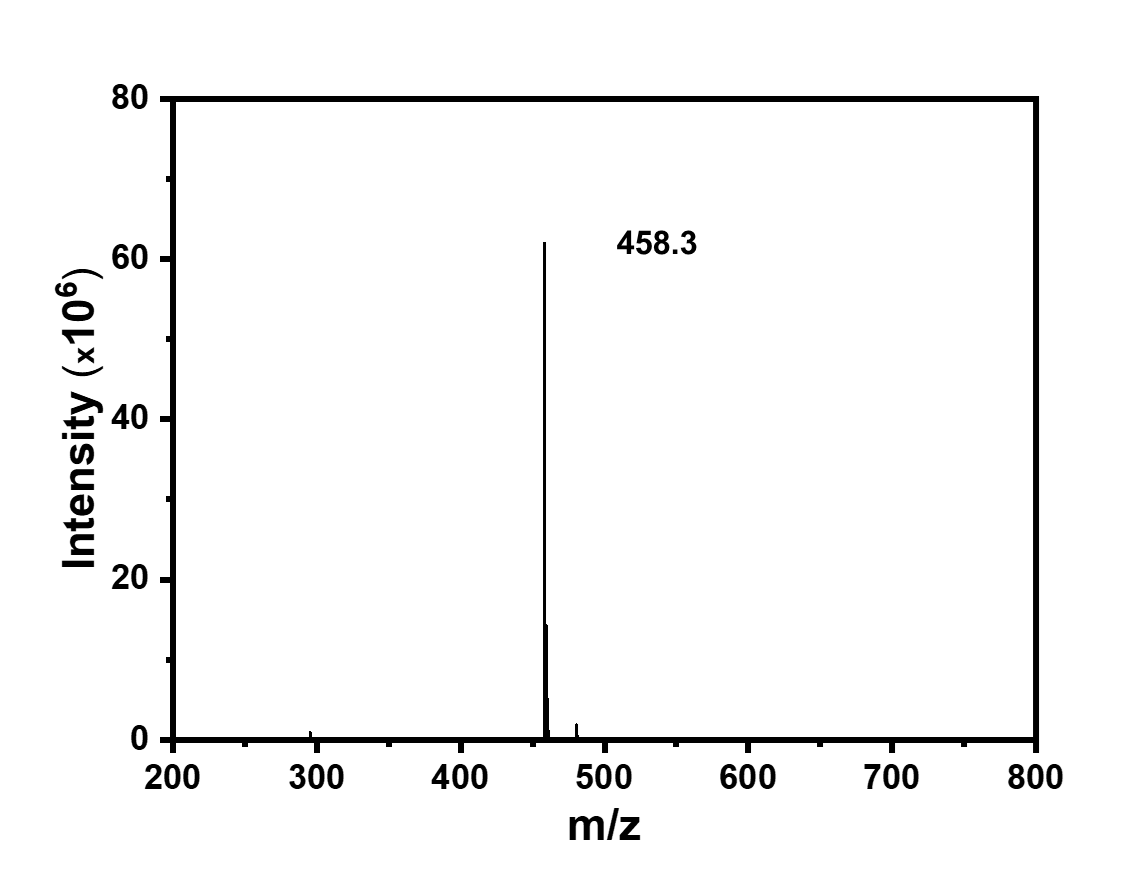


**Figure S16.** ^1^H NMR and LC-MS spectra of FFM (NMRs in DMSO-*d_6_*)

**FFG:** The synthesis steps are the same as previously reported^[2]^.

^1^H NMR (400 MHz, DMSO-*d_6_*): *δ* 9.04 (d, J = 8.3 Hz, 1H), 8.69 (t, *J* = 6.0 Hz, 1H), 8.27 (s, 2H), 7.33 – 7.19 (m, 10H), 4.58 (q, *J* = 3.8 Hz, 1H), 4.02 (s, 1H), 3.91 – 3.83 (m, 2H), 3.63 (s, 3H), 3.17 (dd, *J* = 14.2, 5.1 Hz, 1H), 3.07 (dd, *J* = 14.0, 4.9 Hz, 1H), 2.93 (m, *J* = 33.2, 14.0, 8.1 Hz, 2H). HPLC-MS(EI), calculated for C_21_H_25_N_3_O_4_:384.2 (M+H^+^); found 384.3 (M ^+^).

###
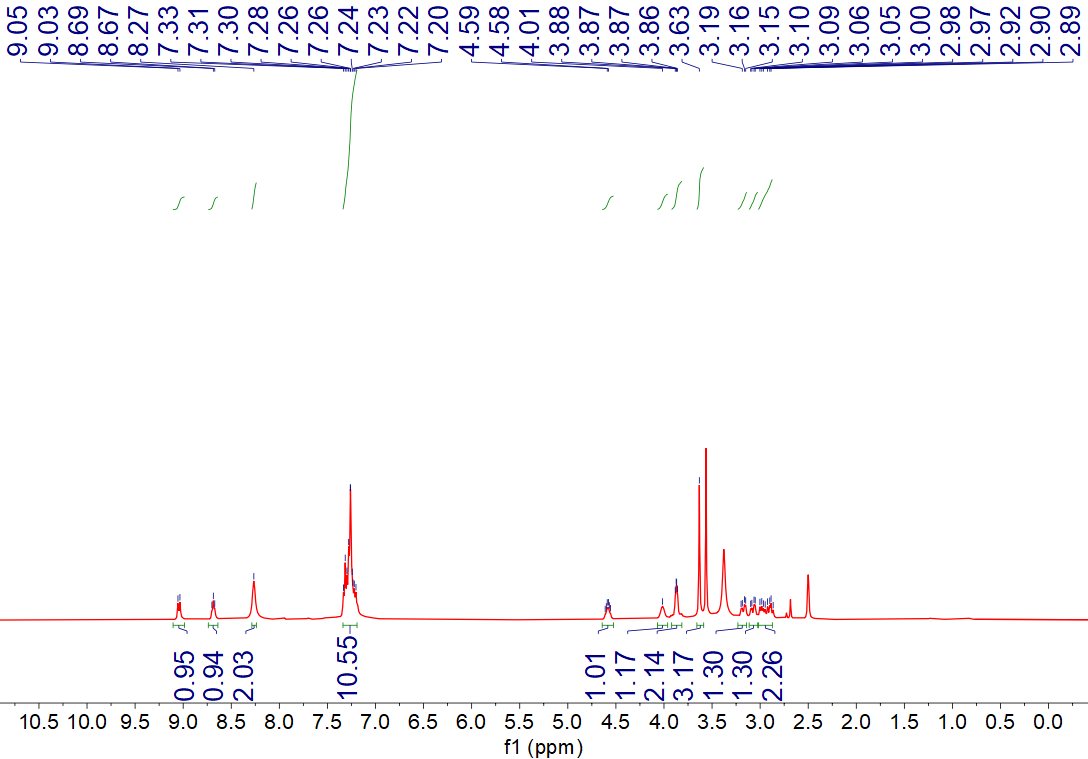


###
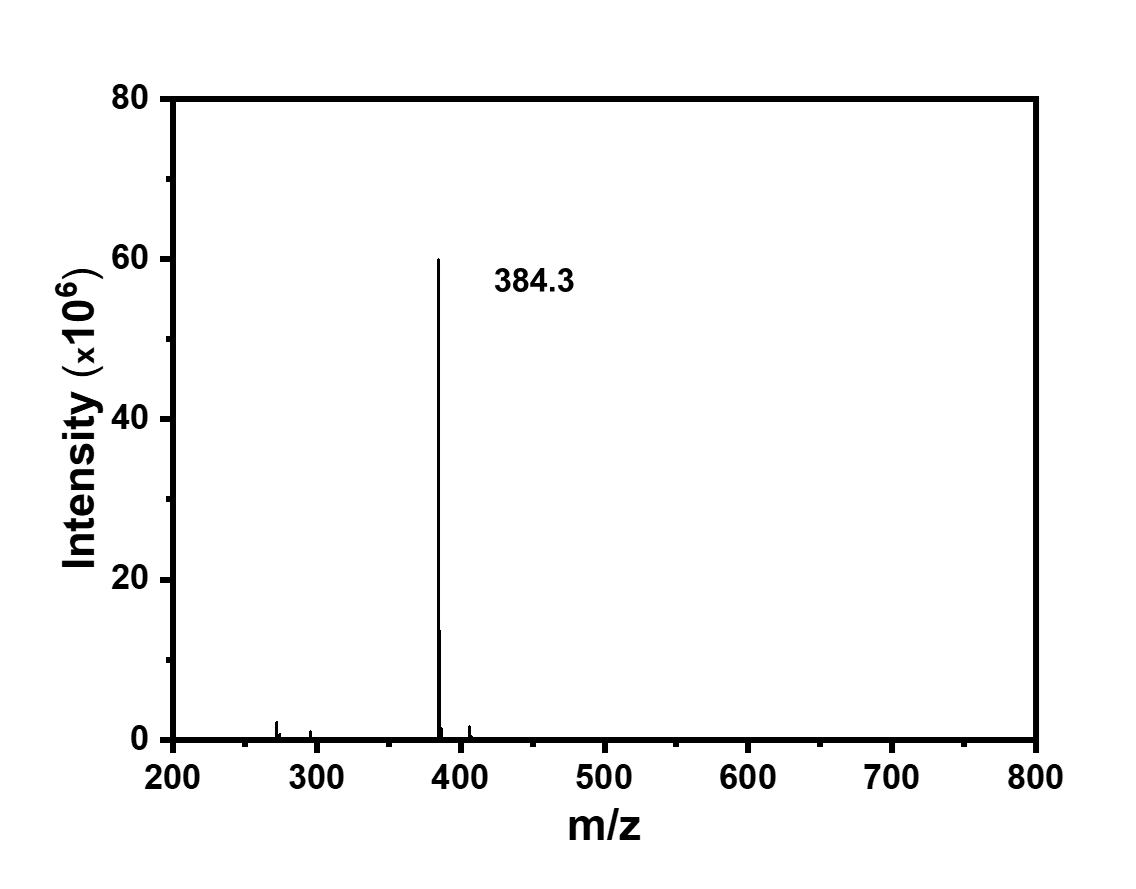


**Figure S17.** ^1^H NMR and LC-MS spectra of FFG (NMRs in DMSO-*d_6_*)

**FFA:** ^1^H NMR (400 MHz, DMSO-*d_6_*): *δ* 9.05 (d, *J* = 8.3 Hz, 1H), 8.71 (d, *J* = 6.9 Hz, 1H), 8.26 (s, 2H), 7.38 – 7.20 (m, 10H), 4.56 (td, *J* = 8.6, 4.7 Hz, 1H), 4.29 (t, *J* = 7.2 Hz, 1H), 4.01 (d, *J* = 6.7 Hz, 1H), 3.60 (s, 3H), 3.20 (dd, *J* = 14.2, 5.1 Hz, 1H), 3.02 (m, *J* = 32.6, 14.1, 6.2 Hz, 2H), 2.87 (dd, *J* = 14.0, 9.1 Hz, 1H), 1.31 (d, *J* = 7.3 Hz, 3H).^13^C NMR (101 MHz, DMSO-*d_6_*): *δ* 171.76, 169.47, 166.82, 136.53, 133.89, 128.72, 128.32, 127.36, 127.12, 125.99, 125.36, 53.20, 52.21, 50.88, 46.66, 36.44, 35.65, 15.89. HPLC-MS(EI), calculated for C_22_H_27_N_3_O_4_:398.2 (M+H^+^): found 398.3 (M ^+^).

###
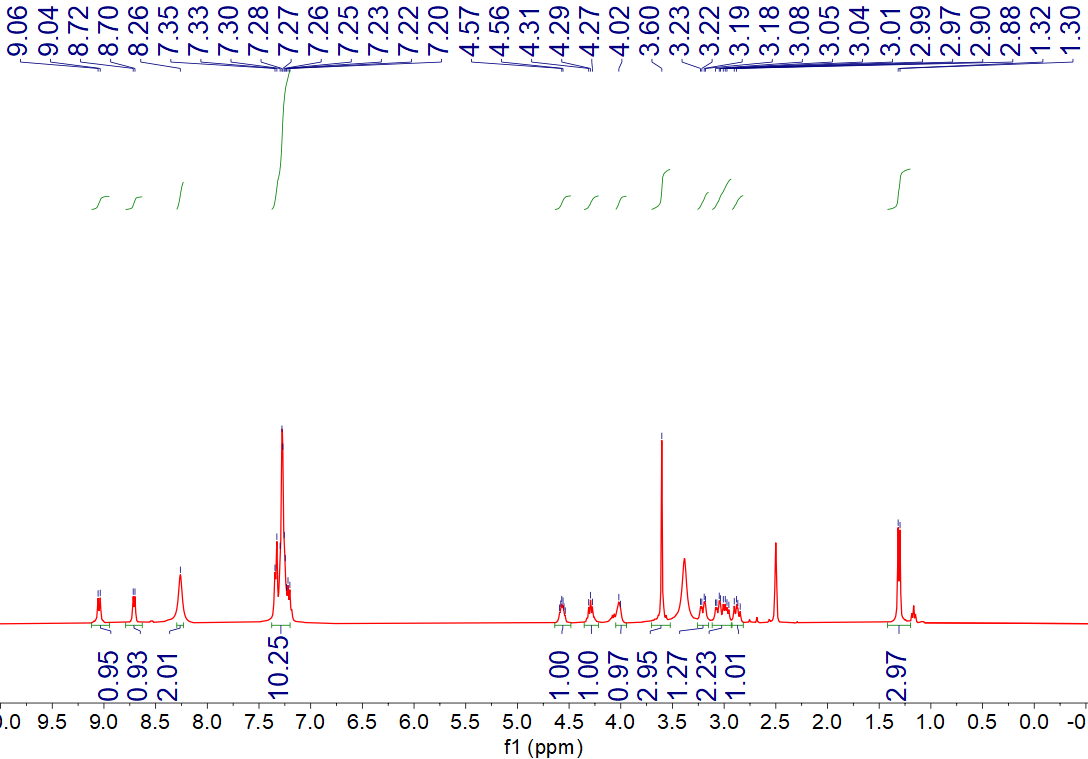


###
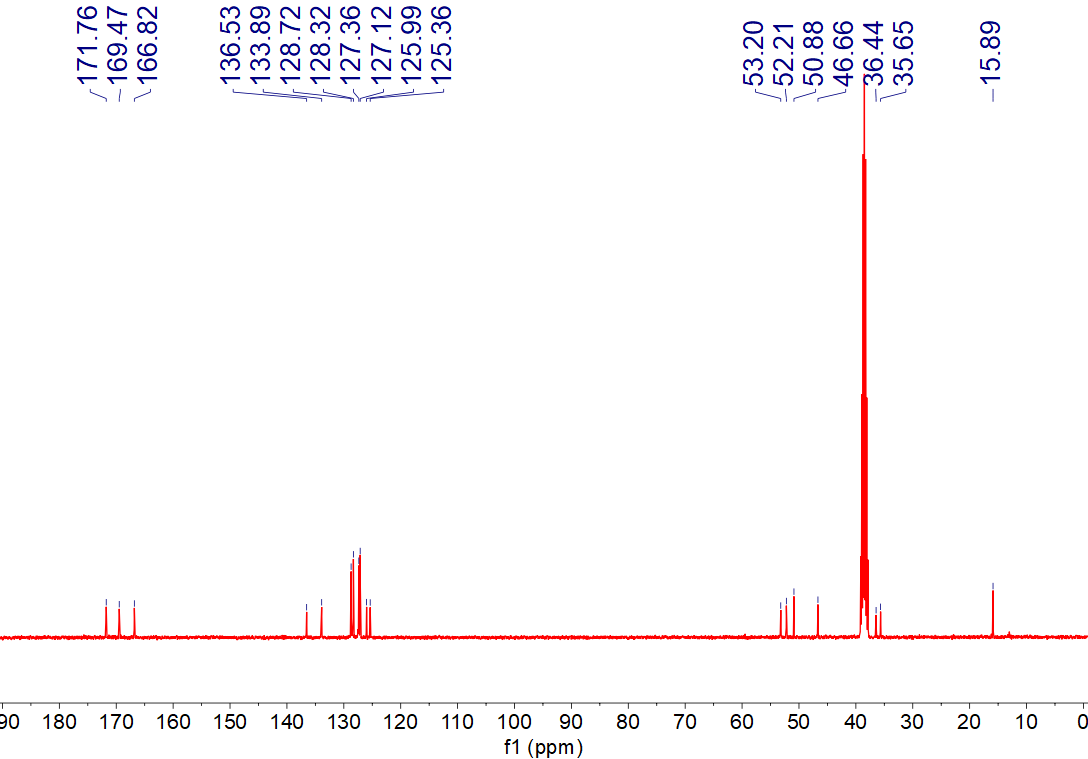


###
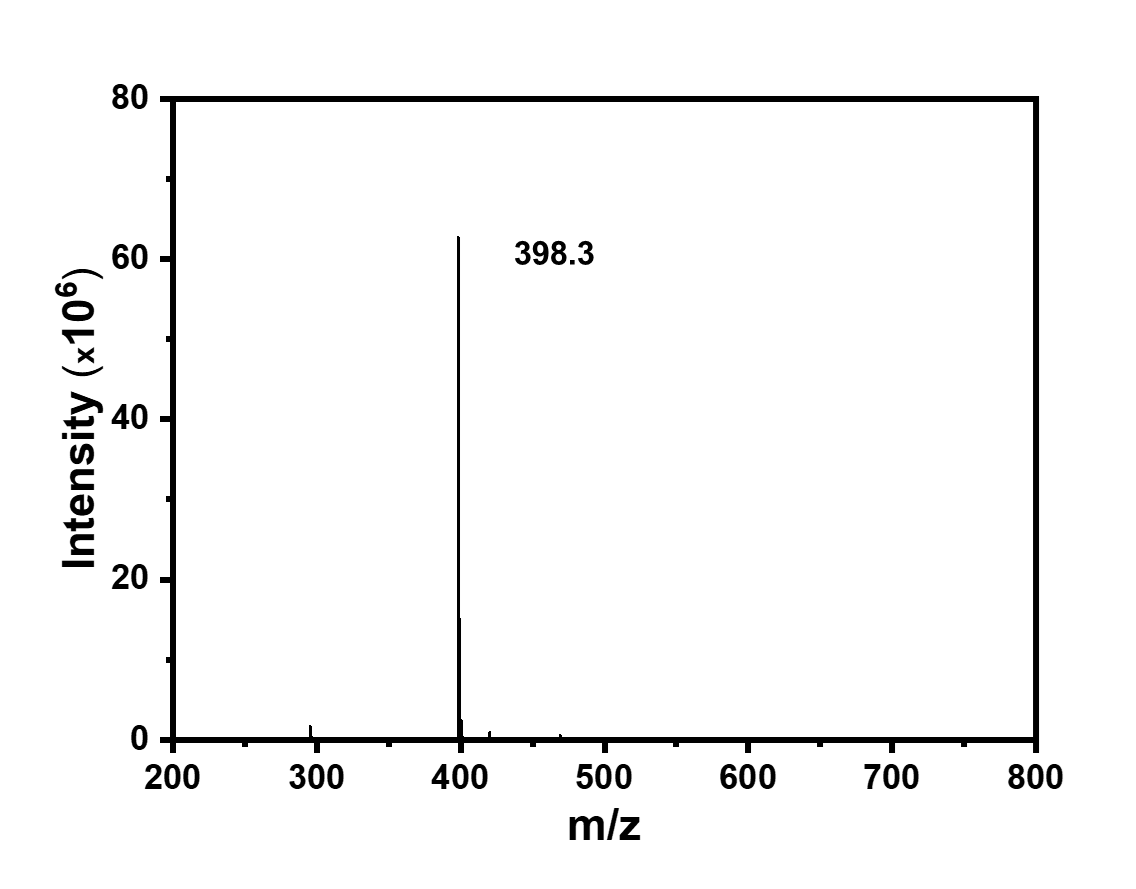


**Figure S18.** ^1^H NMR, ^13^C NMR and LC-MS spectra of FFA (NMRs in DMSO-*d_6_*)

**FFV:** ^1^H NMR (400 MHz, DMSO-*d_6_*): *δ* 9.11 (d, *J* =8.1 Hz, 1H), 8.49 (d, *J* =8.2 Hz, 1H), 8.23 (s, 2H), 7.27 (m, *J* =17.2, 14.4, 10.3 Hz, 10H), 4.68 (q, *J* =3.9 Hz, 1H), 4.22 (t, *J* =7.3 Hz, 1H), 4.01 (d, *J* =6.3 Hz, 1H), 3.61 (s, 3H), 3.23 (dd, *J* =14.2, 4.9 Hz, 1H), 3.06 – 2.87 (m, 3H), 2.07 (h, *J* =6.8 Hz, 1H), 0.90 (dd, *J* =10.4, 6.7 Hz, 6H). ^13^C NMR (101 MHz, DMSO-*d_6_*): *δ* 172.16, 171.52, 168.27, 137.92, 135.28, 130.27, 130.00, 129.79, 128.97, 128.80, 128.56, 127.46, 126.83, 57.97, 54.65, 53.57, 52.17, 38.02, 37.07, 30.37, 19.44, 18.75. HPLC-MS(EI), calculated for C_24_H_31_N_3_O_4_:426.2 (M+H^+^): found 426.4 (M ^+^).

###
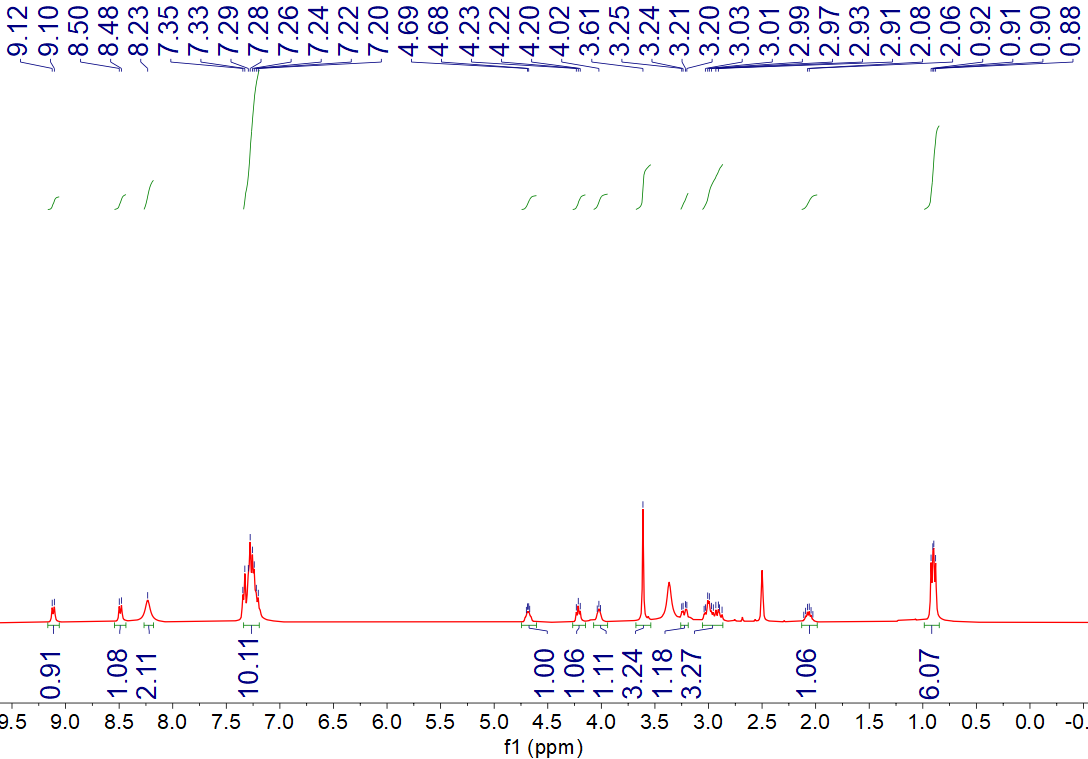


###
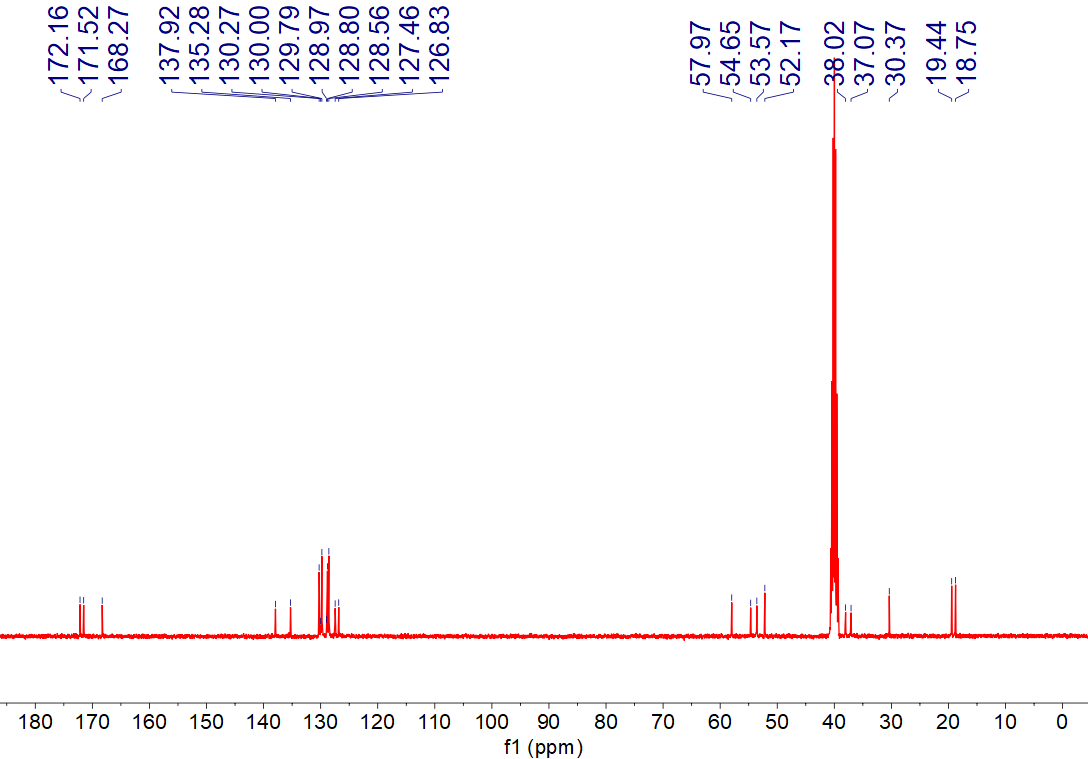


###
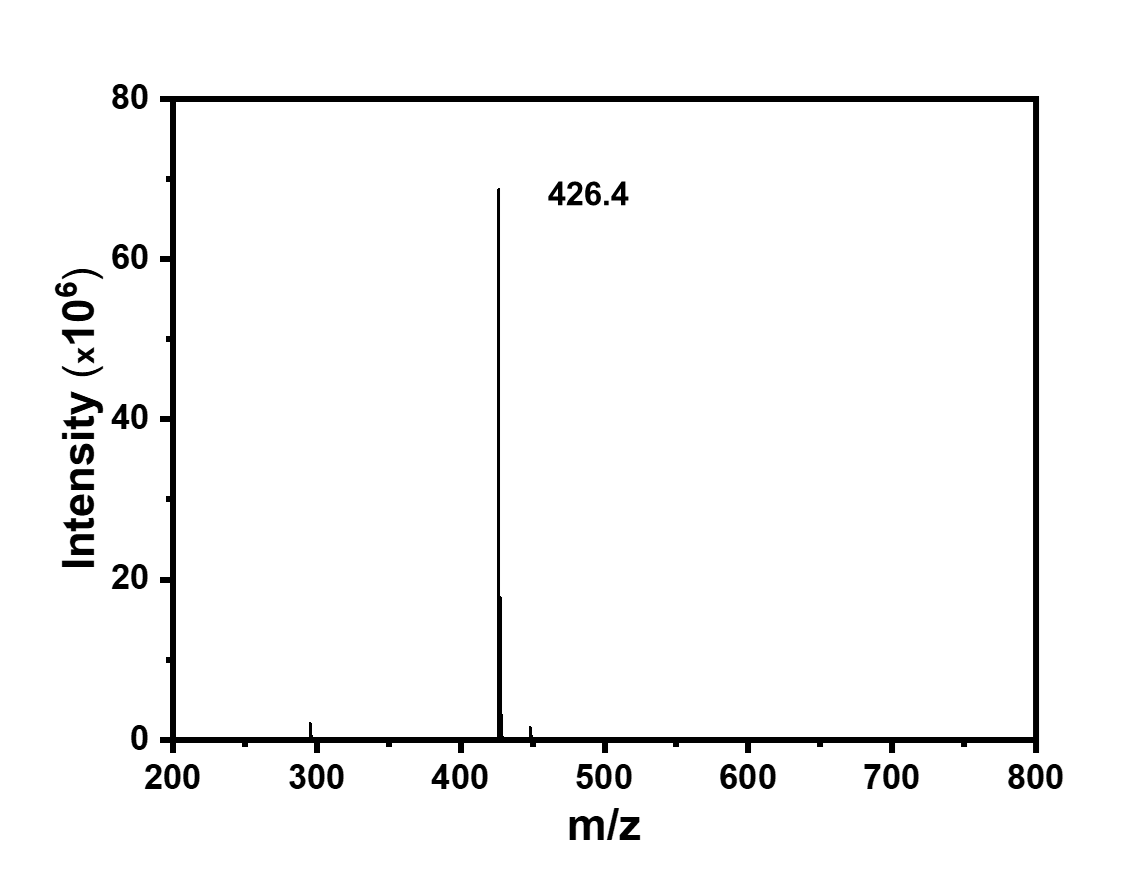


**Figure S19.** ^1^H NMR, ^13^C NMR and LC-MS spectra of FFV (NMRs in DMSO-*d_6_*)

**FFL**: ^1^H NMR (400 MHz, DMSO-*d_6_*): *δ* 8.82 (d, *J* = 8.2 Hz, 1H), 8.40 (d, *J* = 7.7 Hz, 1H), 7.99 (s, 2H), 7.03 (m, *J* = 17.1, 15.3, 9.7 Hz, 10H), 4.36 (q, *J* = 7.4, 6.9 Hz, 1H), 4.08 (q, *J* = 7.6, 6.0 Hz, 1H), 3.79 (s, 1H), 3.36 (s, 3H), 2.98 – 2.92 (m, 1H), 2.77 (m, *J* = 30.8, 14.2, 6.1 Hz, 2H), 2.64 (dd, *J* = 14.0, 9.1 Hz, 1H), 1.41 – 1.24 (m, 2H), 0.85 (t, *J* = 6.7 Hz, 1H), 0.64 (dd, *J* = 20.1, 6.2 Hz, 6H). ^13^C NMR (101 MHz, DMSO-*d_6_*):*δ* 172.84, 172.66, 170.77, 170.59, 167.83, 167.61, 137.46, 137.26, 134.79, 134.66, 129.74, 129.59, 129.33, 129.29, 128.33, 128.13, 128.09, 126.99, 126.51, 126.38, 54.20, 53.97, 53.11, 51.87, 50.30, 50.20, 38.53, 37.55, 36.68, 24.21, 24.06, 22.78, 21.25, 21.19. HPLC-MS(EI), calculated for C_25_H_33_N_3_O_4_: 440.3 (M+H^+^): found 440.4 (M ^+^).

###
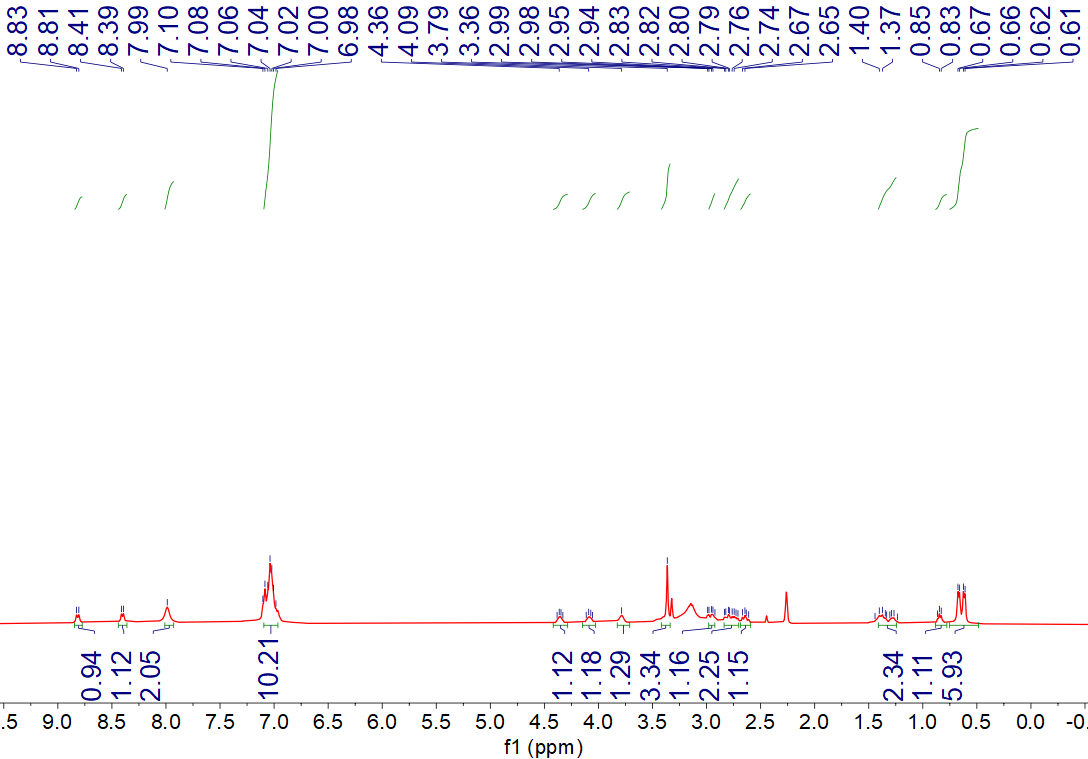


###
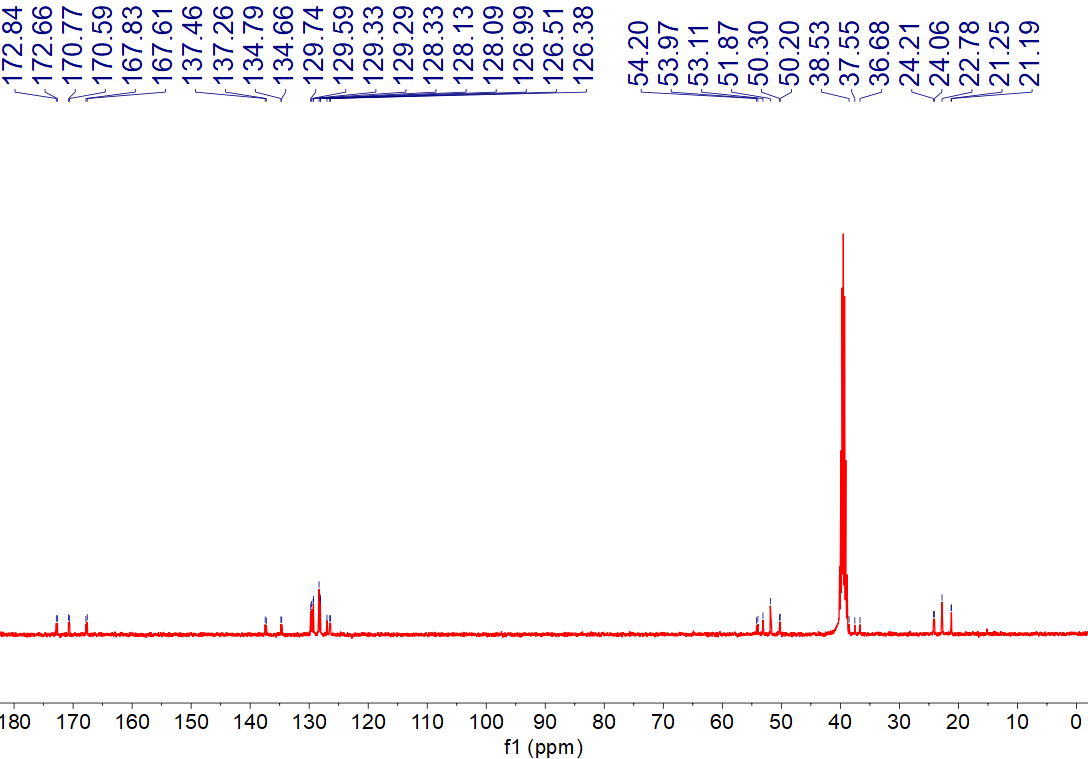


###
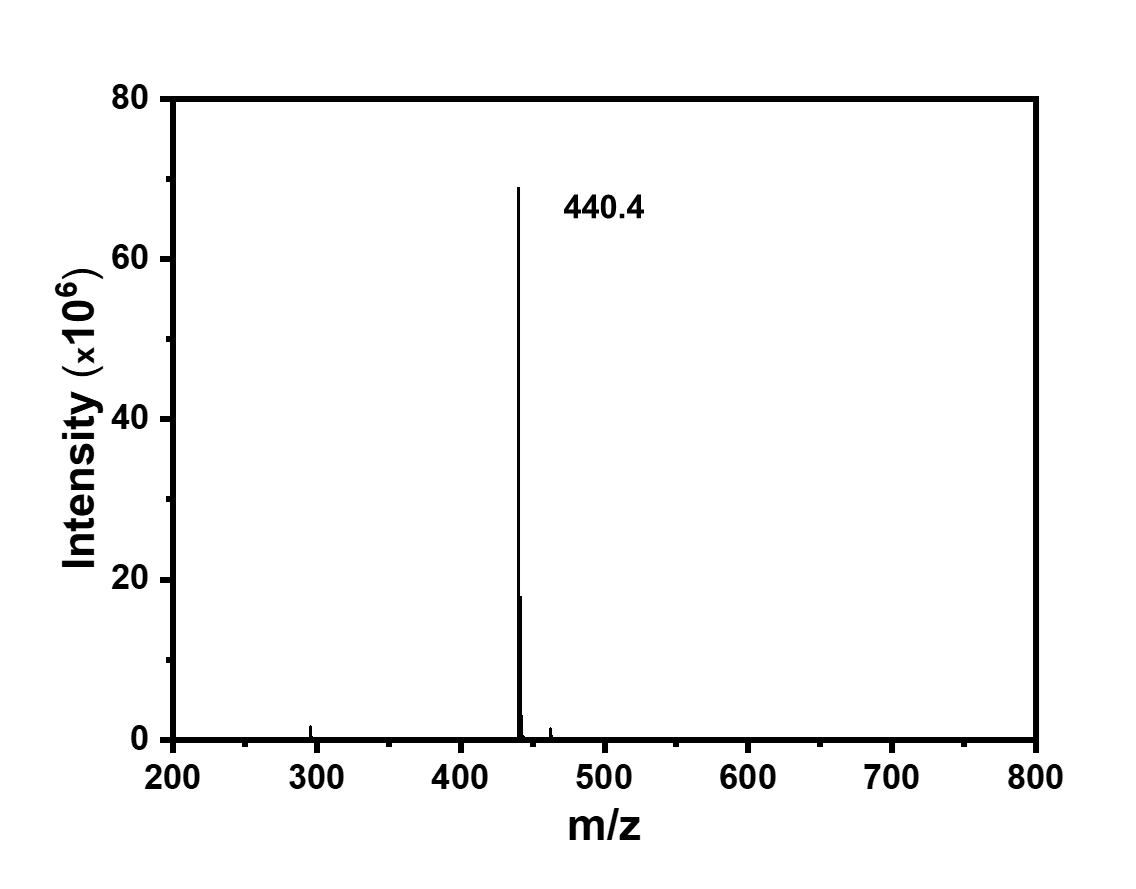


**Figure S20.** ^1^H NMR, ^13^C NMR and LC-MS spectra of FFL (NMRs in DMSO-*d_6_*)

**FFI:** ^1^H NMR (400 MHz, DMSO-*d_6_*):*δ* 9.02 (d, *J* = 8.1 Hz), 8.51 (d, *J* = 8.1 Hz), 8.19 (s, 2H), 7.33 – 7.19 (m, 10H), 4.69 (m, *J* = 8.6, 4.9 Hz, 1H), 4.26 (dd, *J* = 8.0, 6.6 Hz, 1H), 4.02 (dd, *J* = 7.4, 5.0 Hz, 1H), 3.61(s, 3H), 3.20 (dd, *J* = 14.1, 5.0 Hz, 1H), 3.05 – 2.84 (m, 3H), 1.81 (t, *J* = 6.6 Hz, 1H), 1.42 (m, *J* = 13.7, 7.4, 4.4 Hz, 1H), 1.22 (m, *J* = 13.5, 8.7, 7.0 Hz, 1H), 0.92 – 0.78 (m, 6H). ^13^C NMR (101 MHz, DMSO-*d_6_*): *δ* 172.18, 171.42, 168.30, 137.90, 135.29, 130.26, 129.79, 128.82, 128.58, 127.48, 126.85, 66.83, 56.95, 54.58, 53.55, 52.13, 38.72, 38.06, 37.11, 36.72, 25.24, 15.90, 11.61. HPLC-MS(EI), calculated for C_25_H_33_N_3_O_4_:440.3 (M+H^+^); found 440.5 (M ^+^).

###
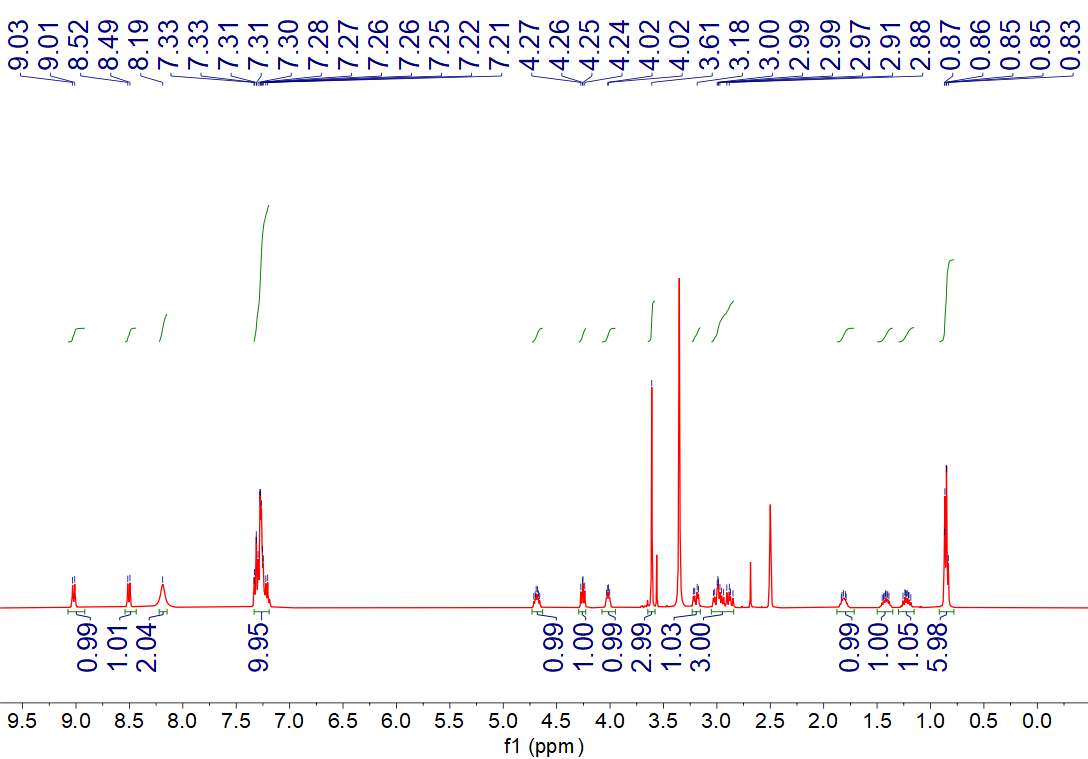


###
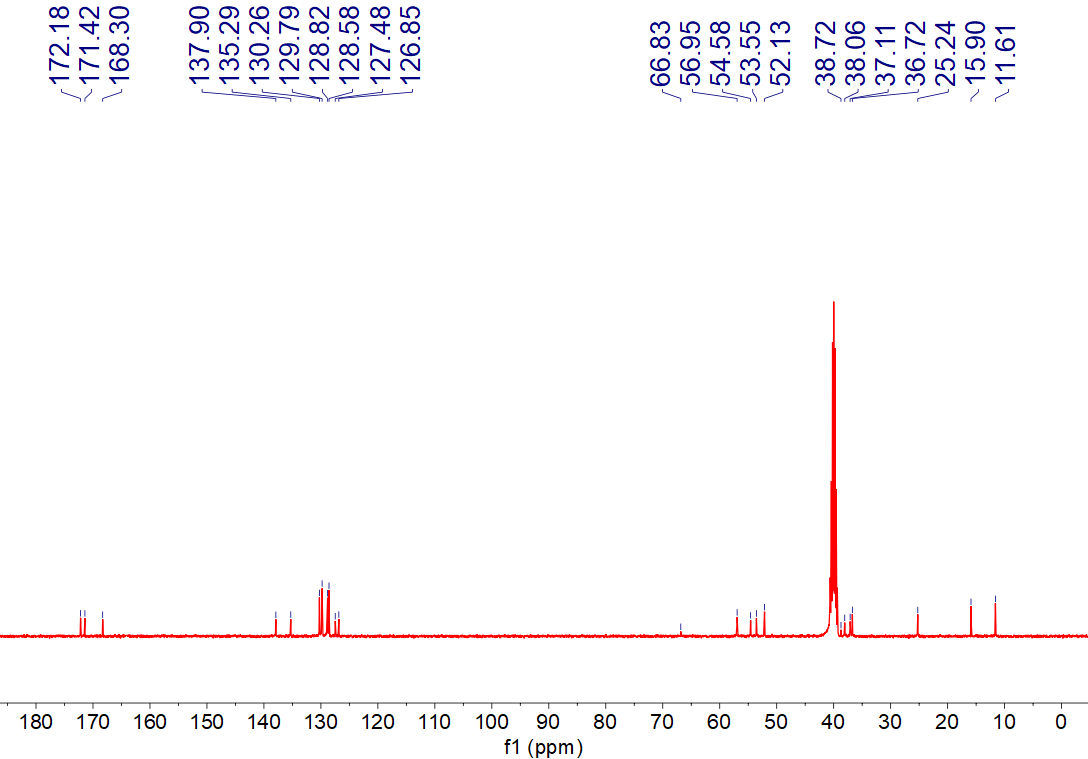


###
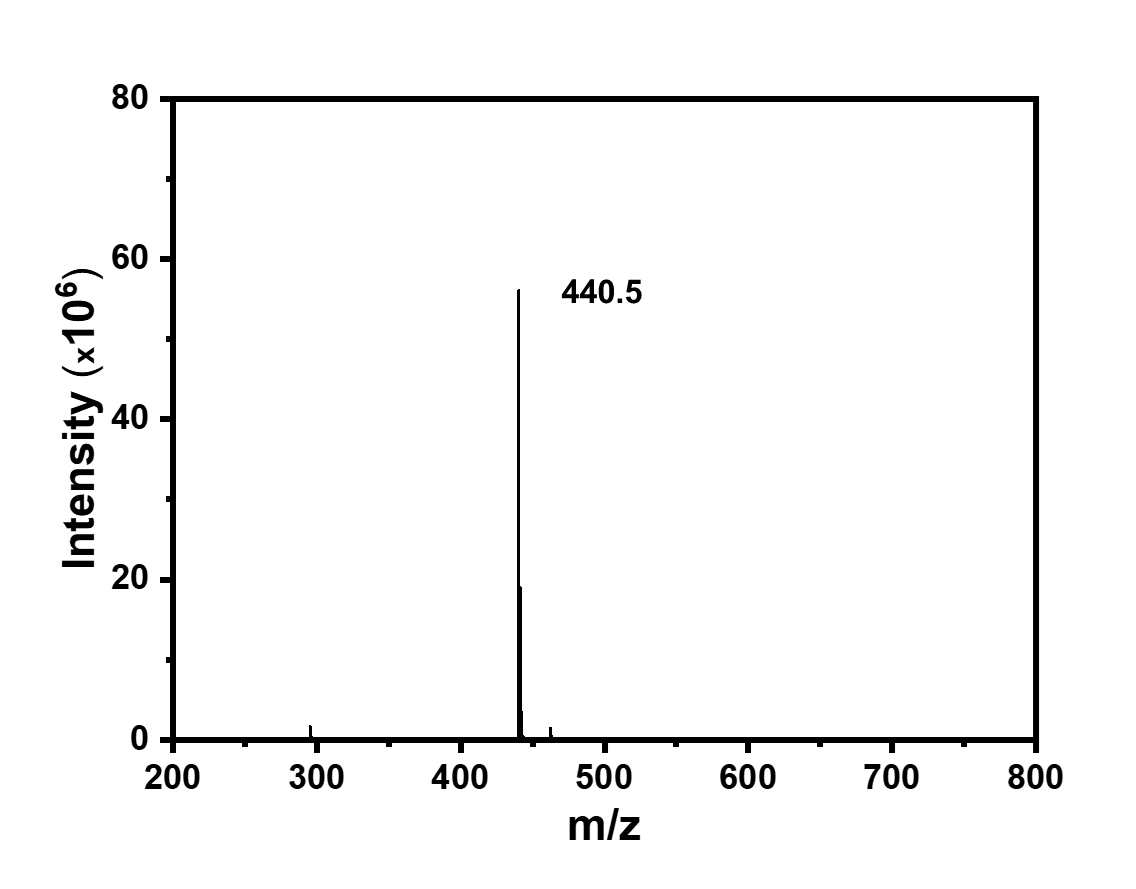


**Figure S21.** ^1^H NMR, ^13^C NMR and LC-MS spectra of FFI (NMRs in DMSO-*d_6_*)

**FFY:** ^1^H NMR (400 MHz, DMSO-*d_6_*): *δ* 9.01 (d, *J* = 8.3 Hz, 1H), 8.70 (d, *J* = 7.5 Hz, 1H), 8.20 – 8.16 (m, 2H), 7.31 – 7.18 (m, 10H), 7.04 (d, *J* = 8.0 Hz, 2H), 6.69 (d, *J* = 7.9 Hz, 2H), 4.60 (q, *J* = 3.8 Hz, 1H), 4.43 (d, *J* = 7.2 Hz, 1H), 3.99 (t, *J* = 6.3 Hz, 1H), 3.56 (s, 3H), 3.19 (dd, *J* = 13.9, 4.8 Hz, 1H), 3.04 – 2.79 (m, 5H).^13^C NMR (101 MHz, DMSO-*d_6_*): *δ* 172.22, 171.17, 168.25, 156.61, 137.88, 135.30, 130.46, 130.18, 129.77, 128.85, 128.58, 127.49, 127.43, 126.84, 115.58, 66.82, 65.39, 54.66, 54.59, 53.69, 52.27, 38.72, 38.09, 37.18, 36.42. HPLC-MS(EI), calculated for C_28_H_31_N_3_O_5_: 490.2 (M+H^+^); found 490.3 (M ^+^).

###
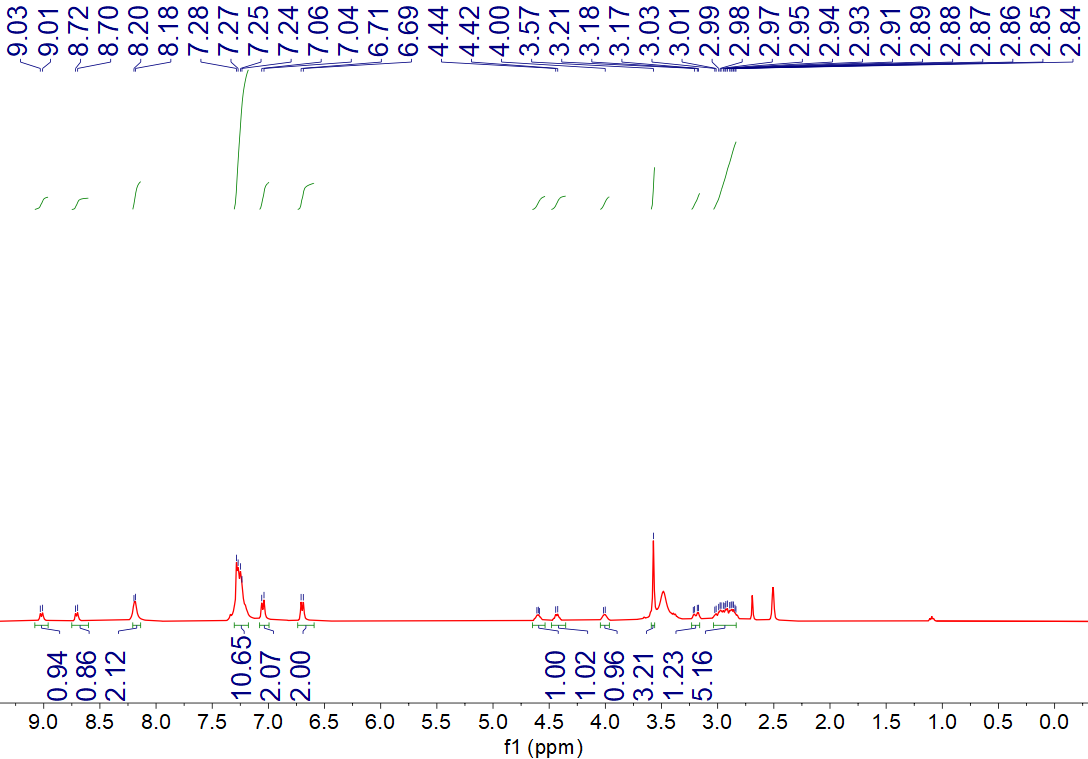


###
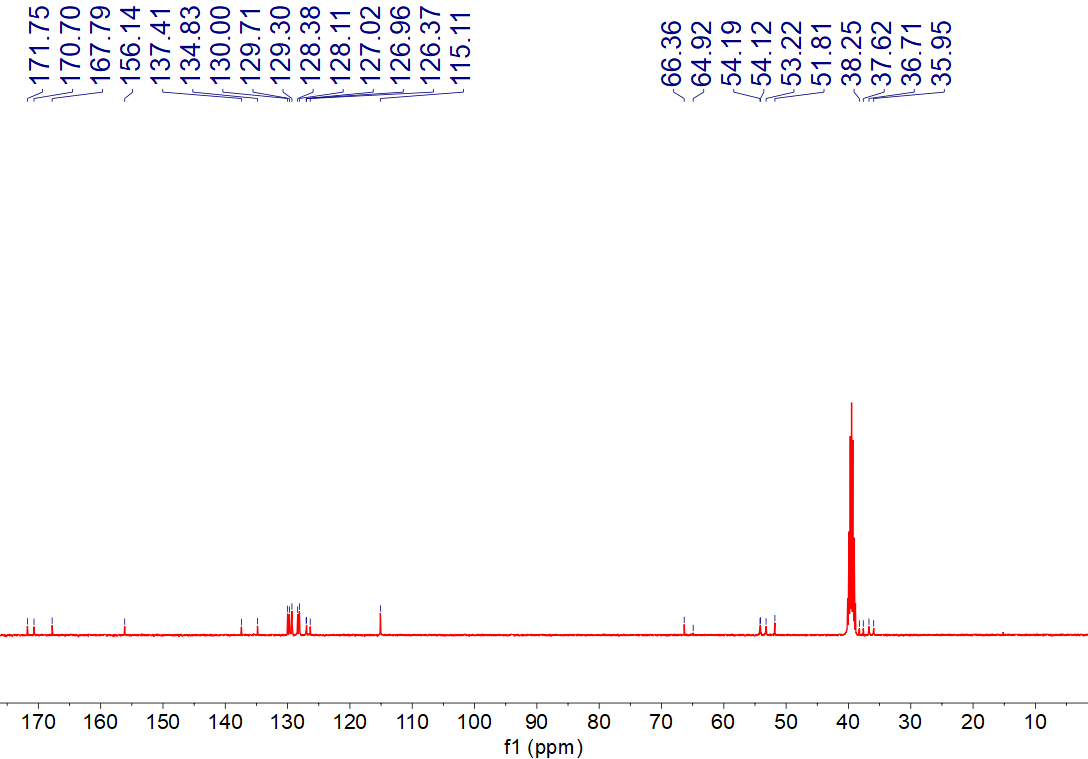


###
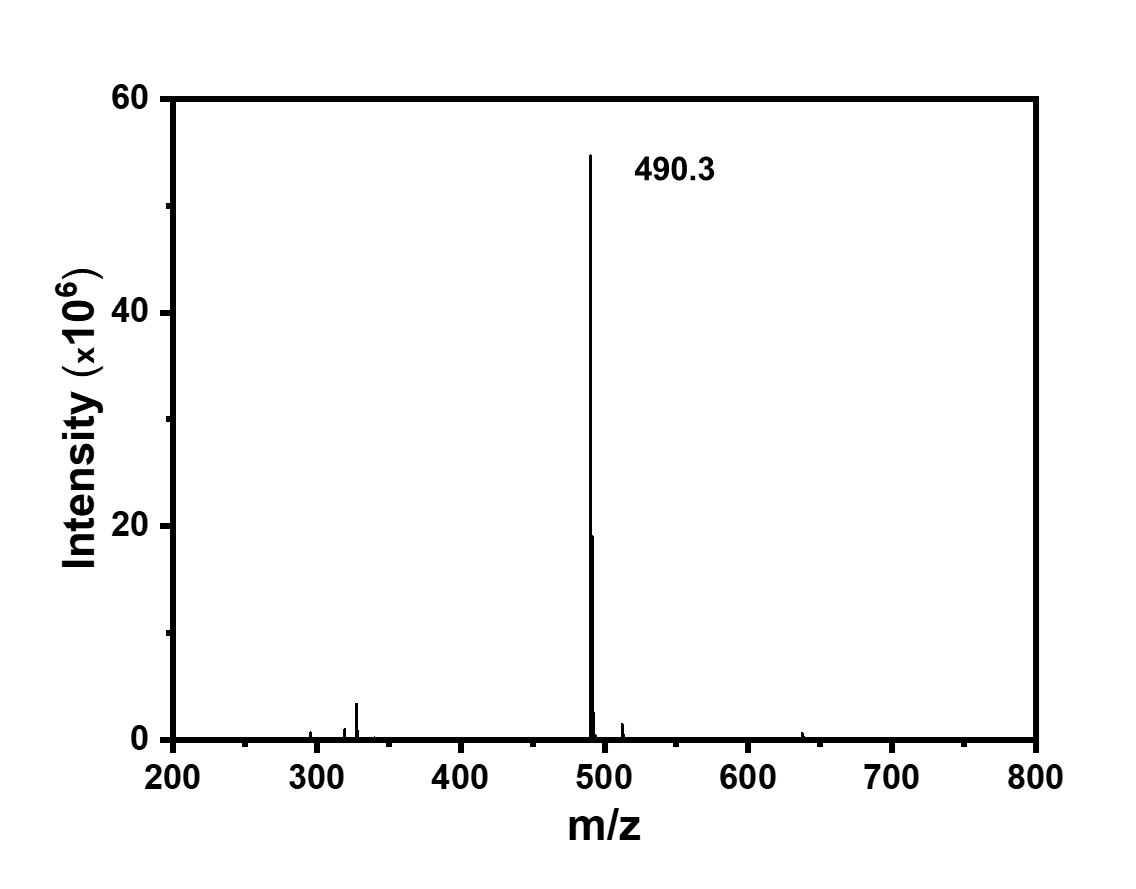


**Figure S22**.^1^H NMR, ^13^C NMR and LC-MS spectra of FFY (NMRs in DMSO-*d_6_*)

###

**Scheme S4**. Synthesis procedure of DTB.

The synthesis steps of DTB are similar to those reported previously^[2]^. A 150 mL Schlenk tube with stirring rod was filled with 25 mL 2 M Na_2_CO_3_, 25 mL toluene, and 9 mL DMF. The solution was then degassed with an argon stream for 15 min, and 4,7-bis(4,4,5,5-tetramethyl-1,3,2-dioxaborolan-2-yl)-2,1,3-benzothiadiazole (1.0 eq, 1.0 g, 2.58 mmol) and 2-bromothiophene (2.5 eq., 1.05 g, 6.44 mmol) and tetrakis(triphenylphosphane) palladium (0.02 eq., 59.55 mg, 51.53 µmol) were added in the presence of an argon flow. The reaction mixture was heated to 90 °C for 36 h with an attached reflux condenser while stirring vigorously. After cooling to room temperature, 30 mL of Milli-Q water was added, followed by extraction with dichloromethane (4 x 25 mL), washing with brine, and drying over Na_2_SO_4_. After evaporation of all volatiles with a rotary evaporator, the crude mixture was purified by SiO2 column chromatography (gradient from 10% DCM: 90% petroleum ether to 70% DCM: 30% petroleum ether). The product was obtained as a red powder.

^1^H NMR (400 MHz, DMSO-*d_6_*): *δ* 8.23 – 8.03 (m, 1H), 7.76 (d, *J* = 5.0 Hz, 1H), 7.26 (d, *J* = 4.6 Hz, 1H).

###

**Scheme S5**. Synthesis procedure of 4-azidobenzyl (4-nitrophenyl) carbonate.

In general, the synthesis method was based on previous studies^[3]^. 4-Azidobenzyl alcohol (19.8 mg, 0.13 mmol) was placed in a vial, and subsequently, 1 mL of dichloromethane and 70 μL triethylamine were added. After stirring for 10 min, 4-Nitrophenyl chloroformate (53.6 mg, 0.266 mmol) was added, and the reaction was carried out at room temperature for 1 h. At the end of the reaction, a fast column chromatographic separation (PE: DCM 1:1) gave a white solid product. The structure was characterized by nuclear magnetic resonance spectroscopy.

^1^H NMR (400 MHz, CDCl_3_): *δ* 8.28 (d, *J* = 8.6 Hz, 2H), 7.44 (d, *J* = 8.1 Hz, 2H), 7.37 (d, *J* = 8.9 Hz, 2H), 7.07 (d, *J* = 8.1 Hz, 2H), 5.26 (s, 2H).

###

**Scheme S6**. Synthesis procedure of N_3_-coumarin.

The synthesis method was based on previous studies^[3]^. To a solution of 4-azidobenzyl-4-nitrophenyl carbonate (31.4 mg, 0.1 mmol) in anhydrous DMF (2 mL) was added coumarin (38.4 mg, 0.22 mmol) and triethylamine (120 μL, 0.4 mmol). The reaction mixture was stirred in the dark at 25 °C under an atmosphere of nitrogen for 24 h, after which time the DMF was diluted with water and extracted with ethyl acetate. The combined organic fractions were washed with water, brine, dried, and concentrated in vacuo (temperature maintained below 30 °C). The crude residue was subjected to flash silica gel column chromatography (PE: EA= 4:1) to provide the title compound as a white solid (23 mg, 66%). Small amounts of 4-nitrophenol co-eluted with the product, but these were removed through rinsing with ice-cold methanol (product is partially soluble in methanol) or performing a miniature liquid-liquid extraction (ethyl acetate: water). Following the liquid-liquid extraction, the product was isolated as a white solid (15 mg, 43%).

^1^H NMR (400 MHz, CDCl_3_): *δ* 7.61 (d, *J* = 8.7 Hz, 1H), 7.44 (d, *J* = 8.0 Hz, 2H), 7.22 (d, *J* = 2.4 Hz, 1H), 7.15 (dd, *J* = 8.8, 2.2 Hz, 1H), 7.06 (d, *J* = 8.1 Hz, 2H), 6.28 (s, 1H), 5.26 (s, 2H), 2.43 (s, 3H).

^13^C NMR (101 MHz, CDCl_3_): *δ* 160.51, 154.30, 153.35, 152.90, 151.90, 141.07, 131.12, 130.59, 125.64, 119.49, 118.19, 117.45, 114.86, 110.07, 70.34.

###
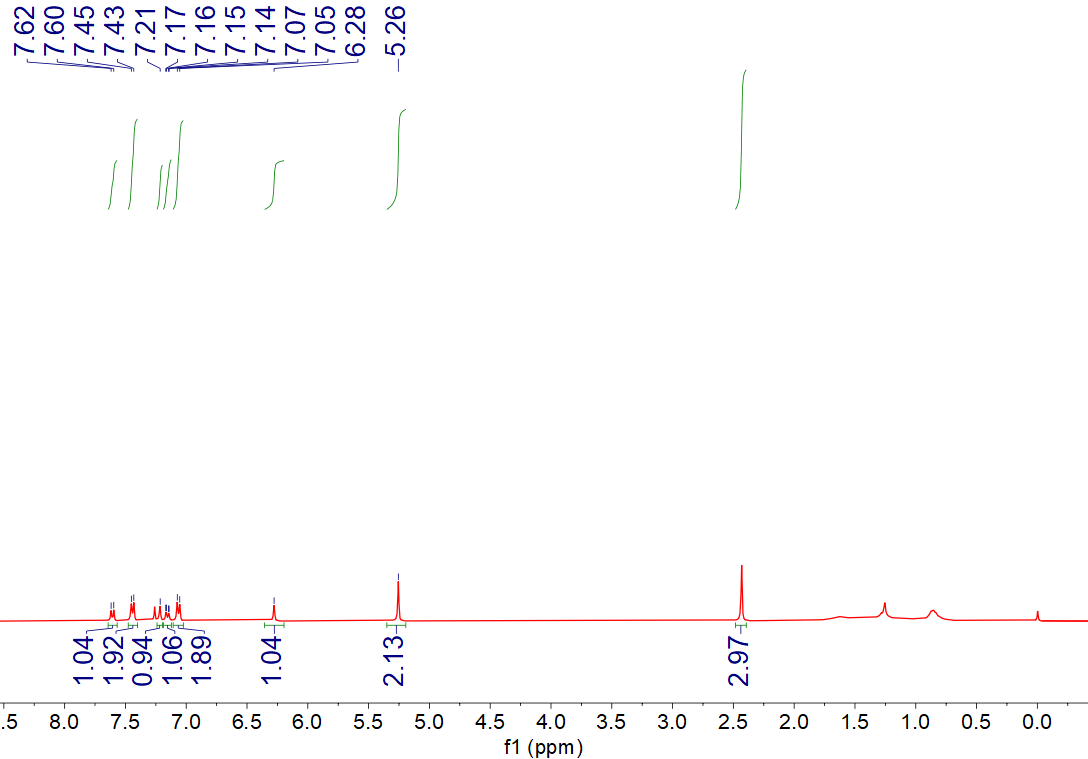


###
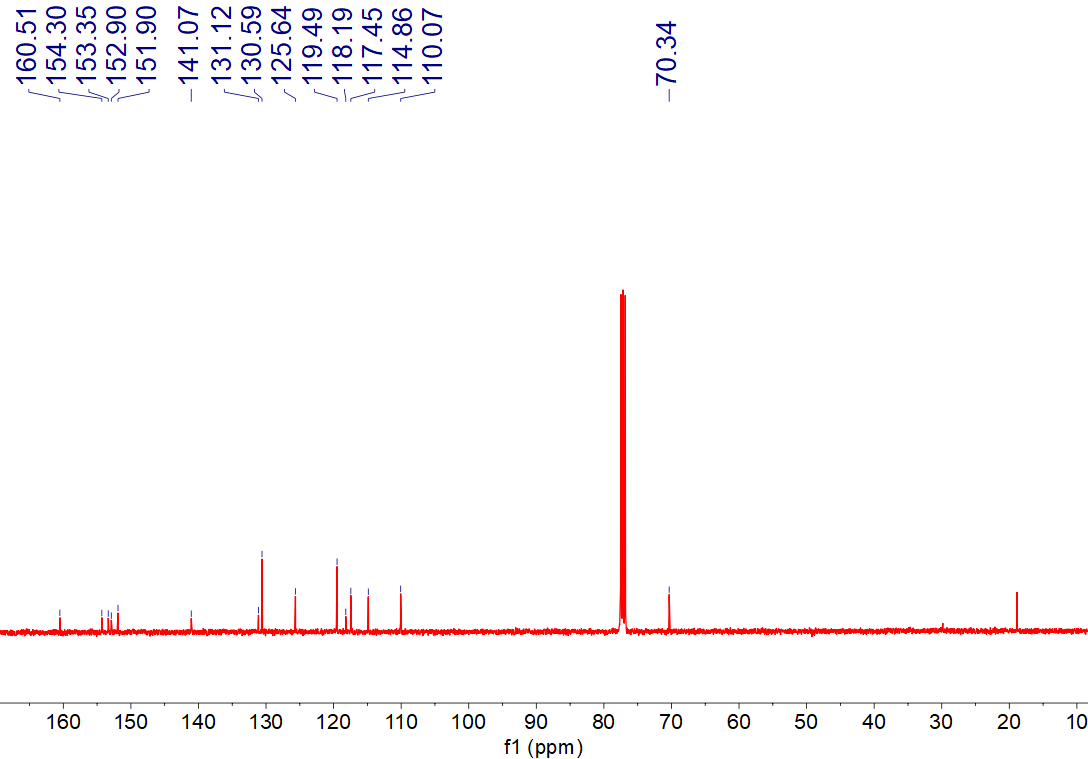


**Figure S23.** ^1^H NMR, ^13^C NMR of N_3_-coumarin (NMRs in CDCl_3_)

###

**Scheme S7.** Synthesis procedure of N_3_-g-coumarin.

Using the methods of previous research^[4]^, 4-Methylumbelliferyl beta-D-galactoside (16.9 mg, 0.05 mmol) and (4-Azidophenyl) methyl 4-nitrophenyl carbonate (31.4, 0.1 mmol) were combined, along with DMAP (12.2 mg, 0.1 mmol) were added to a round-bottomed flask and dissolved using ultra-dry DMF (1 ml) under nitrogen atmosphere, and the reaction was carried out at 60 °C for 12 h. At the end of the reaction, the reaction was cooled down to room temperature, precipitated with water, and then centrifuged to collect the pozzolanic colored solid, which was subsequently recrystallized to give a white solid.

^1^H NMR (400 MHz, CDCl_3_): *δ* 7.57 – 7.29 (m, 6H), 7.07 – 6.69 (m, 6H), 6.24 – 6.10 (m, 1H), 5.22 – 5.09 (m, 4H), 4.56 – 4.43 (m, 1H), 4.12 (q, J = 7.1 Hz, 4H), 3.91 – 3.53 (m, 3H), 2.38 (s, 3H).

###
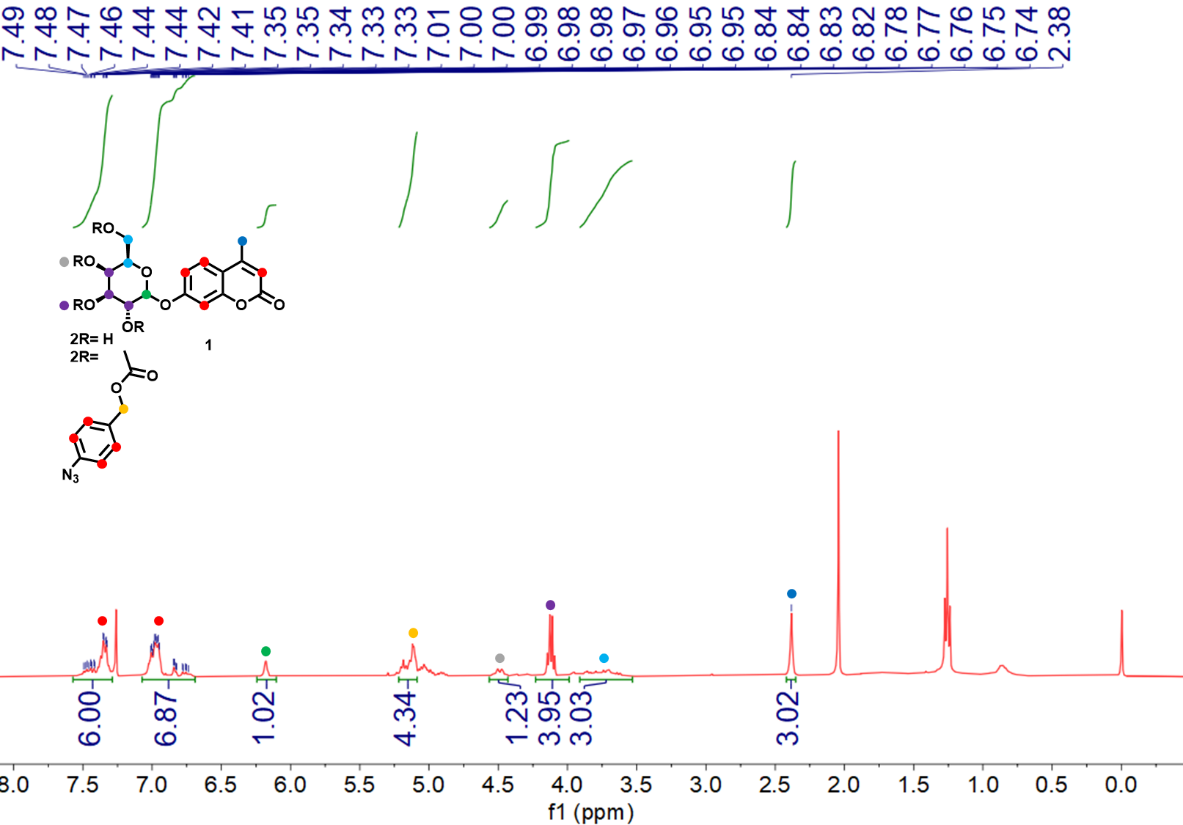


**Figure S24.** ^1^H NMR of N_3_-g-coumarin (NMRs in CDCl_3_)

**FITC labeling of BSA and β-Gal:** In brief, 10 mg BSA or β-Gal were dissolved in 2 mL of sodium carbonate buffer (100 mM, pH 8.8). Then 250 μL of FITC solution (1.0 mg mL^-1^, in DMSO) was added slowly in 5 μL aliquots. The mixture was stirred at R.T. for 3 h, incubated at 4 ^o^C for 8 h in the dark, and followed by purification with dialysis (Medicell dialysis tubing, MWCO 12-14 kDa) against Milli-Q water and then freeze dried.

**Synthesis of BSA@MnO_2_ nanoparticles:** The synthesis of BSA-stabilized MnO_2_ nanoparticles was performed according to the reported literature with slight modifications. In brief, a 4 mL KMnO_4_ solution (5 mM) was added to a 25 mL round-bottom flask, which was purged with N_2_ and bubbled for 30 minutes at RT. Then an 8 mL solution of Na_2_S_2_O_3_ (1.875 mM) was added at a rate of 1 mL min^-1^ with a syringe pump. The mixture solution was stirred for 10 minutes. Then, 3 mL BSA solution (7 mg mL^-1^) was added dropwise. Then the solution was stirred for another hour, and the solution was dialyzed against MilliQ (Spectrapor, MWCO: 1000 KDa) to remove excess BSA. Samples were stored in the 4 ^o^C fridge before use.

## 4. Peptide droplet formulations and guest cargoes encapsulation

**4.1 Preparation of peptide coacervates:** We select FMF as a model molecule, and all the other peptides were the same products to form coacervate. First, we used 5 mM Hepes buffer (pH ~ 6) to dissolve peptides into a 20 mg mL^-1^ solution after heating. Then the 20 mg mL^-1^ peptide solution was diluted to 5 mg mL^-1^ using 5 mM Hepes buffer, and a few drops of 0.1 M NaOH were added to reach pH ~ 8 to induce coacervation. 10 μL of white emulsion was dropped onto a slide for microscopic observation (Glass setup was under below).


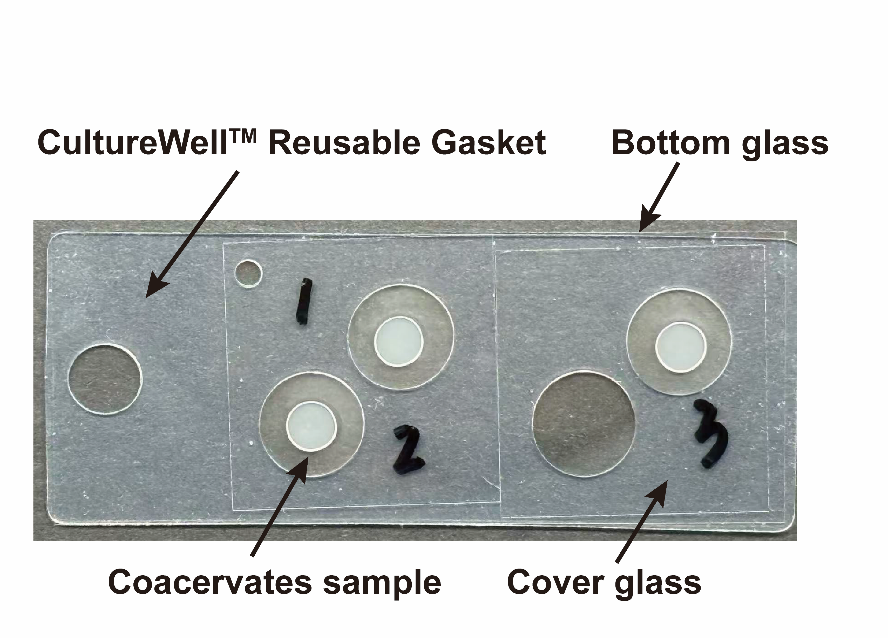


Glass setup for droplet observation with microscopy imaging and confocal imaging. In detail, a Grace Bio-Labs CultureWell™ chambered coverglass (GBL103340, Sigma) was placed on the top of a glass (24*60 mm). Droplet samples (10~20 μL) were placed in the chamber and a coverglass (22*22 mm) was placed on the top to prevent droplet evaporation. Coacervates were sedimented onto the surface of glass slide under the influence of gravity, where they can be observed.

**4.2 Turbidity measurement:** All turbidity-based measurements were performed on a Tecan multimode plate reader. Turbidity was utilized as the indicator of phase separation of samples, in which the droplet formation was further confirmed by optical microscopy. Absorption at 600 nm is used as the wavelength for all turbidity measurements, and all measurements were performed at room temperature. After the sample addition and mixing for 5 s, the turbidity value was recorded.

**4.3 Water content (Q_w_):** The method testing water content (Q_w_) of coacervate was according to literature reports from Spruijt group^[5]^. A 5 mL solution of coacervates (5 mg mL^-1^ FMF peptides, 10.9 mM, 5 mM Hepes buffer, pH~8) was separately put in five centrifuge tube which were weighed beforehand (Q_o_). Then coacervates were subjected to centrifugation at 3000 rpm for 40 min, followed by an hour of equilibration. After centrifugation, the dilute phase was transparent and the coacervate phase was displayed as a semitransparent phase, consistent with previous reports. Then the diluted phase was discarded and filter paper was then utilized to wipe the moisture from the tube walls. The tubes were weighted again (Q_1_) before placing into a vacuum oven at 120 °C for 20 h. The dry tubes were weighed again (Q_2_).

$$Qw=100\%\cdot(Q1-Q2)/(Q1-QO)$$

**Table S1**. Water content (Q_w_) of FMF coacervates.

| peptide | Water content (% w/w) |
| --- | --- |
| FMF | **80.4 ± 6.4** |

**4.4 Enzyme-controlled generation and disappearance of coacervates:** All turbidity-based measurements were performed on a Tecan multimode plate reader.

**(i) Enzyme-controlled generation of coacervate:** A solution of FMF peptide (5 mg mL^-1^, 10.9 mM) at pH ~6 was added to the 96-well plate. For enzyme-controlled coacervation, 1 μL of urease solution (final concentration 0.02 g L^-1^) in 50 μL of sample was added, then different volumes of urea (stock concentration:0.5 M), including 0.2 μL (final concentration ~ 2 mM urea), 0.4 μL (final concentration ~ 4 mM urea), 0.6 μL (final concentration ~ 6 mM urea), 0.8 μL (final concentration ~ 8 mM urea) and 1 μL (final concentration ~ 10 mM urea) were added to the samples and mixed. The turbidity changes were monitored with the plate reader. The peptide solution without urea was used as a blank sample. The experiments were repeated three times.

**(ii) Reversible regulation of coacervate generation and disappearance using urea/urease and hydrochloric acid:** Similar to the previous step, 50 μL of the FMF (5 mg mL^-1^, 10.9 mM) at pH~6 and 1 μL of 1 g L^-1^ urease solution were first added to a 96-well plate. Then 1 μL of 1M urea solution was added and mixed for a few seconds. The turbidity changes were then monitored for 10 minutes. After 10 minutes, the well plate was taken out, and then 1 μL of 0.5 M HCl solution and 1 μL of urea solution were added to the sample. After mixing for a few seconds, the turbidity changes were measured for another 10 minutes. The experiments were repeated three times.

**(iii) Enzyme-controlled disappearance of coacervate:** FMF coacervates (5 mg mL^-1^, 10.9 mM, pH ~8) was prepared and added to the 96-well plate. Then 1 μL of 10 g L^-1^ GOx solution (final concentration 0.2 g L^-1^) was added to the above 50 μL of sample. Different volumes of glucose solution (stock concentration: 1 M) was added in the above samples including 0.25 μL (final concentration: 5 mM glucose), 0.75 μL (final concentration: 15 mM glucose), 1 μL (final concentration: 20 mM glucose), 1.5 μL (final concentration: 30 mM glucose) and mixed for a few seconds. The changes in turbidity were then monitored. The coacervate solution without glucose was selected as a blank sample. The experiments were repeated three times.

**4.5 Encapsulation of protein:** All experiments were performed at room temperature unless indicated otherwise. All of the protein and enzyme solutions were stored at -20 ^o^C before use. In detail, 10 μL of the prepared coacervate solution (5 mg mL^-1^, 10.9 mM) was mixed with 0.5 μL FITC-BSA (4 mg mL^-1^), and then the encapsulation was confirmed with confocal microscopy using the Leica stellaris 5. The droplet sample preparation method for confocal images has been described in Section 4.1.

**4.6 Partitioning of guest molecules:** All experiments were performed at room temperature unless indicated otherwise. In brief, the coacervate droplets were first prepared by adding 1 M NaOH solution into the peptide solution in 5 mM Hepes buffer. Then 100 μL of the prepared coacervate solution (5 mg mL^-1^, 10.9 mM) was mixed with the 0.5 μL dye solutions (1 mg mL^-1^ in DMSO or Milli-Q, final concentration 0.005 mg mL^-1^) by pipetting. Then the mixture was dropped on a glass surface with a cover glass using a home-made setup. The droplet sample preparation method for confocal images has been described in Section 4.1. Then droplets were imaged with confocal microscopy using the Leica stellaris 5.

**4.7 Measurement of dye adsorption efficiency：**All experiments were performed at room temperature unless indicated otherwise. In brief, 1.5 μL dye solutions (2 mg mL^-1^ in DMSO or Milli-Q, final concentration 0.01 mg mL^-1^) was loaded into a 300 μL coacervate solution (5 mg mL^-1^ peptides, 10.9 mM, 5 mM Hepes buffer, finally dye concentration about 0.01 mg mL^-1^). The coacervate solution was then subjected to a low-speed centrifugation to yield two phases. Then DMSO and Hepes buffer were then added to the two phases to obtain solutions with a DMSO-to-Hepes buffer ratio close to 1:1. Subsequently, the fluorescence spectra of the diluted phase and concentrated phase were measured and utilized to determine the dye adsorption efficiency.

**4.8 Dynamic regulation of coacervate adsorption of guest dyes using urea/urease and hydrochloric acid:** All experiments were performed at room temperature. A 100 μL FMF solution (5 mg mL^-1^, 10.9 mM) at pH~6 was prepared. Then 2 μL of 1 g L^-1^ urease and 0.5 μL of 1 mg mL^-1^ Nile red solution were added. 2 μL of 1M urea was added to the above solution and mixed. Then 10 μL of the solution was placed on the cover glass for observation. After 10 min, 2 μL of 0.5 M HCl solution was added to the stock solution, mixed for a few seconds, and 10 μL of the solution was placed on the cover glass for observation. Then, another 2 μL of 1 M urea solution was added, mixed for a few seconds, and 10 μL of the mixture was placed on the cover glass for observation. The droplet sample preparation method for confocal images has been described in Section 4.1. All samples were observed using the Leica stellaris 5.

**4.9 Fluorescence recovery after photobleaching (FRAP):** All experiments were performed at room temperature. Briefly, the solution of coacervate droplets was first prepared by adding 0.1 M NaOH solution to the peptide solution in 5 mM Hepes buffer. Then, 20 μL of the prepared coacervate dispersion (5 mg mL^-1^, 10.9 mM, 100 mM NaCl) was mixed with 0.1 μL Nile red solution (0.2 mg mL^-1^ in DMSO, final concentration 0.001 mg mL^-1^) by pipetting. The mixture was then dropped onto a glass surface with a coverslip using a homemade setup to avoid evaporation even after days of incubation. For FRAP measurements, the fluorescence intensities of the peptide coacervates were quenched to 30%-40% of the initial level, and then the recovery of the fluorescence intensity of the droplets was tracked using the Leica stellaris5. The droplet sample preparation method for confocal images has been described in Section 4.1.

## 5. Biomimicry application with peptide droplets

All reactions were analyzed quantitatively and qualitatively at ambient conditions using a Tecan multimode plate reader and CLSM.

**5.1 Azide reduction in peptide coacervates:**

###

(i) Micro-plate reader measurements: To a 100 μL peptide solution (5 mg mL^-1^, 10.9 mM, pH ~7, 100 mM NaCl), 0.3 μL Ph_3_P solution (76 mM in DMSO, final concentration 0.228 mM) was added via pipetting. After equilibrium for 2 min, the mixture solution was added with 0.5 μL 4-azidobenzyl (4-Methylumbelliferone) carbonate (57 mM in DMSO, final concentration 0.285 mM). The fluorescent intensity change (λ*_em_*= 450 nm) was monitored with a microplate reader.

(ii) Confocal imaging measurements: To a 20 μL peptide solution (5 mg mL^-1^, 10.9 mM, pH ~7, 100 mM NaCl), 0.1 μL Ph_3_P solution (38 mM in DMSO, final concentration 0.19 mM) was added via pipetting. After equilibrium for 2 min, the mixed solution was added with 0.2 μL 4-azidobenzyl (4-Methylumbelliferone) carbonate solution (57 mM in DMSO, final concentration 0.57 mM). The droplet sample preparation method for confocal images has been described in Section 4.1. After that, the emission intensity at λ_em_= 450 nm was then recorded via confocal imaging using the Leica stellaris 5.

**5.2 Cascade reaction in peptide coacervates:**

###

(i) Microplate reader measurements: To a 50 μL peptide solution (5 mg mL^-1^, 10.9 mM, pH ~7, 100 mM NaCl), 0.3 μL Ph_3_P solution (38 mM in DMSO, final concentration 0.228 mM) and 0.3 μL *β*-Gal (500 U mL^-1^ in Tris buffer, final concentration 3 U mL^-1^) were added via pipetting. After equilibrium for 2 min, the mixed solution was added with 0.5 μL azido masked galactose-coumarin derivative **1** solution (29 mM in DMSO, final concentration 0.29 mM). Change of fluorescent intensity (λ*_em_*= 450 nm) was monitored through a micro-plate reader.

(ii) Confocal imaging measurements: To a 20 μL peptide solution (5 mg mL^-1^, 10.9 mM, pH ~7, 100 mM NaCl), 0.1 μL Ph_3_P solution (38 mM in DMSO, final concentration 0.19 mM) and 0.1 μL *β*-Gal solution (500 U mL^-1^ in Tris buffer, final concentration 2.5 U mL^-1^) were added via pipetting. After equilibrium for 2 min, the mixed solution was added with 0.2 μL azido masked galactose-coumarin derivative **1** solution (29mM mL^-1^ in DMSO, final concentration 0.29 mM). The droplet sample preparation method for confocal images has been described in Section 4.1. After that, the emission intensity at λ_em_= 450 nm was then recorded via confocal imaging using the Leica stellaris 5.

## 6. As sub-organelles inside complex coacervate-based synthetic cells

**6.1 Membrane-bound complex coacervates formation:** The formation of complex coacervates was adapted from the literature report with slight modifications, which utilized the complex coacervation between Q-Am and C-Am, then the membrane-less complex coacervates were stabilized with BSA@MnO_2_ nanoparticles. In brief, Q-Am and CM-Am were dissolved in PBS buffer with a concentration of 2.5 mg mL^-1^. Coacervation was induced by mixing the solutions of Q-AM and C-AM in a ratio of 1:1. Then, 8 μL of BSA@MnO_2_ nanoparticles solution were added to the solution by pipetting. After that, the stability of the BSA@MnO_2_ stabilized complex coacervate was checked via optical microscopy.

**6.2 Integration of peptide-coacervates as sub-organelle:** 8 μL of FMF peptide-coacervates (5 mg mL^-1^, 10.9 mM) was first prepared by adding a few amounts of NaOH solution to the FMF peptide solution. Then 10 μL of C-AM (2.5 mg mL^-1^ in PBS, pH~7) was added and mixed via pipetting for a few seconds. After that, 10 μL of Q-AM (2.5 mg mL^-1^ in PBS, pH~7) was added and mixed via pipetting for a few seconds. Then a solution of 8 μL of BSA@MnO_2_ was added, followed by pipetting for a few seconds. The resultant complex was then subject to optical microscopy to observe the multi-compartmentalized structure.

**6.3 Enzyme-controlled generation and disappearance of coacervates as active sub-organelles:** All experiments were performed at room temperature.

First, membrane-bound artificial cells containing enzymes and peptide molecules inside were prepared. The procedure is as follows: 4 μL of peptide solution (20 mg mL^-1^, 43.8 mM) was first prepared, then 10 μL C-AM (2.5 mg mL^-1^ in PBS, pH ~7) was added and pipetted for a few seconds. After that, 10 μL of Q-AM (2.5 mg mL^-1^ in PBS, pH~7) was added and mixed via pipetting for a few seconds to obtain membrane-less complex coacervates. Then, 1 μL of 1 g L^-1^ urease (final concentration, 0.03 g L^-1^) and 1 μL of 10 g L^-1^ GOx (final concentration, 0.3 g L^-1^) were added to regulate the pH of the solution. 0.3 μL FITC-BSA (4 mg mL^-1^, final concentration 0.01 mg mL^-1^) and 0.1 μL Nile Red (0.2 mg mL^-1^, final concentration 0.0006 mg mL^-1^) were added to show the distribution of artificial cells and whether subcellular organelles were produced or not. At last, 8 μL BSA@MnO_2_ was added to the stabilized complex coacervate.

For the generation of coacervates as sub-organelles, 0.5 μL of 0.5M urea (final concentration: 8 mM) was added to the above solution to raise the pH. And the generation of sub-organelles inside artificial cells was observed with a confocal microscope.

For the disassembly of internal coacervates, 1 μL of 1 M glucose (Final concentration 30 mM) was added to the artificial cell solution with sub-organelles inside. And the disassembly of the internal organelles was tracked with a confocal microscope.

**6.4 Cascade reaction in the multi-compartmentalized system:** 8 μL peptide solution (5 mg mL^-1^, 10.9 Mm, pH ~8) was treated with 0.1 μL Ph_3_P (38 mM, final concentration 0.114 mM). Then, 10 μL of C-AM solution (2.5 mg mL^-1^ in PBS) was added and mixed by pipetting for a few seconds. 10 μL of Q-AM solution (2.5 mg mL^-1^ in PBS) was then added and mixed to obtain complex coacervate. 0.3 μL of *β*-Gal solution (final concentration 4 U mL^-1^) was added as enzyme catalysis. 0.3 μL FITC-BSA (4 mg mL^-1^, final concentration 0.03 mg mL^-1^) was added to show the distribution of artificial cells. After that, 8 μL BSA@MnO_2_ was added to stabilize the complex coacervate. At last, the system was treated by 0.2 μL azido masked galactose-coumarin derivative **1** (29 mM in DMSO, final concentration 0.145 mM). The droplet samples were prepared using the method described in Section 4.1, followed by observation the progression of cascade reaction in multicompartment artificial cells under confocal microscopy. The emission wavelength at λ_em_= 450 nm (for observing organelles) and 525 nm (for observing artificial cells) was recorded via confocal imaging using the Leica stellaris 5.

## 7. The assembly behaviors of tripeptides

| **Peptide abbreviation** | **~pH 8** | **Peptide abbreviation** | **~pH 8** | **Peptide abbreviation** | **~pH 8** |
| --- | --- | --- | --- | --- | --- |
| **GFF** | **aggregate** | **FGF** | **coacervate** | **FFG** | **coacervate** |
| **AFF** | **aggregate** | **FAF** | **coacervate** | **FFA** | **coacervate** |
| **VFF** | **aggregate** | **FVF** | **fiber-like** | **FFV** | **coacervate** |
| **IFF** | **aggregate** | **FIF** | **fiber-like** | **FFI** | **coacervate** |
| **LFF** | **aggregate** | **FLF** | **coacervate** | **FFL** | **coacervate** |
| **MFF** | **aggregate** | **FMF** | **coacervate** | **FFM** | **fiber-like** |
| **FFF** | **fiber-like** | **FFF** | **fiber-like** | **FFF** | **fiber-like** |
| **YFF** | **spherical aggregate** | **FYF** | **aggregate** | **FFY** | **aggregate** |

**Table S2**. The assembly behaviors of tripeptides. All peptides were dissolved in 5 mM Hepes buffer.

###
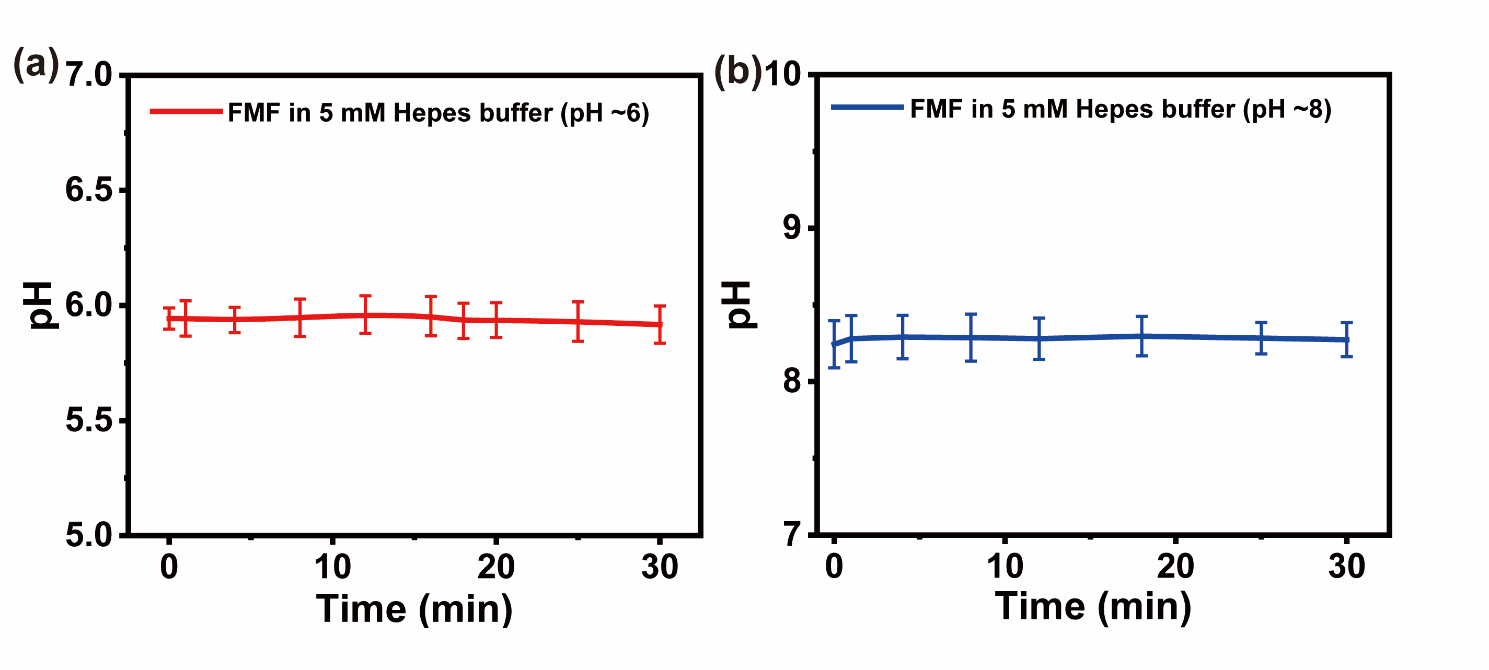


**Figure S25**. pH stability of the 5 mM Hepes solution with FMF (5 mg mL^-1^) over 30 minutes incubation at pH ~6 (a) and ~8 (b).

###
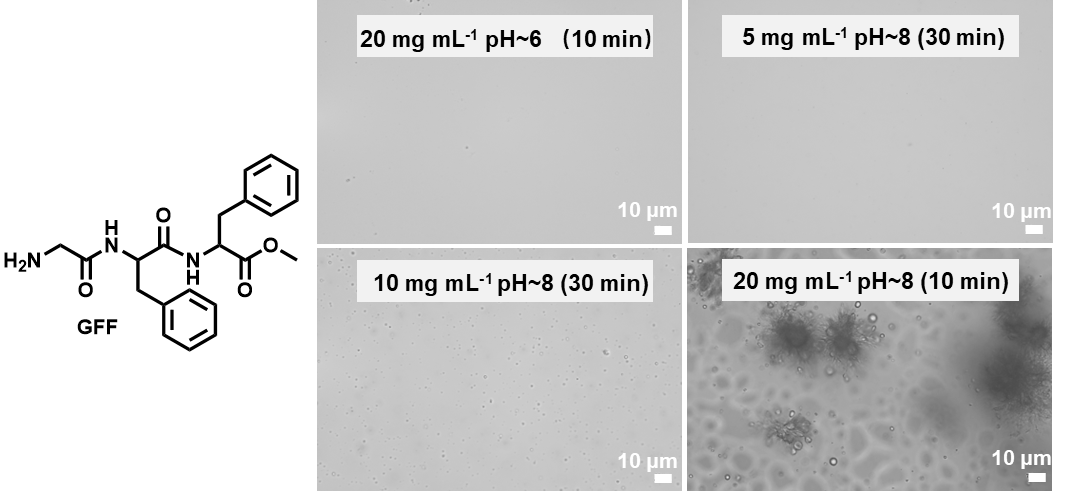


**Figure S26**. Left: Chemical structure of the tripeptide GFF; Right: GFF dissolved at pH ~ 6 (in 5 mM Hepes buffer) and exhibits concentration-dependent assembly behavior at pH ~8, forming aggregates at high concentrations (20 mg mL^-1^). Scale bar = 10 μm in the microscopy image. Similar results were obtained with 3 samples measured independently.

###
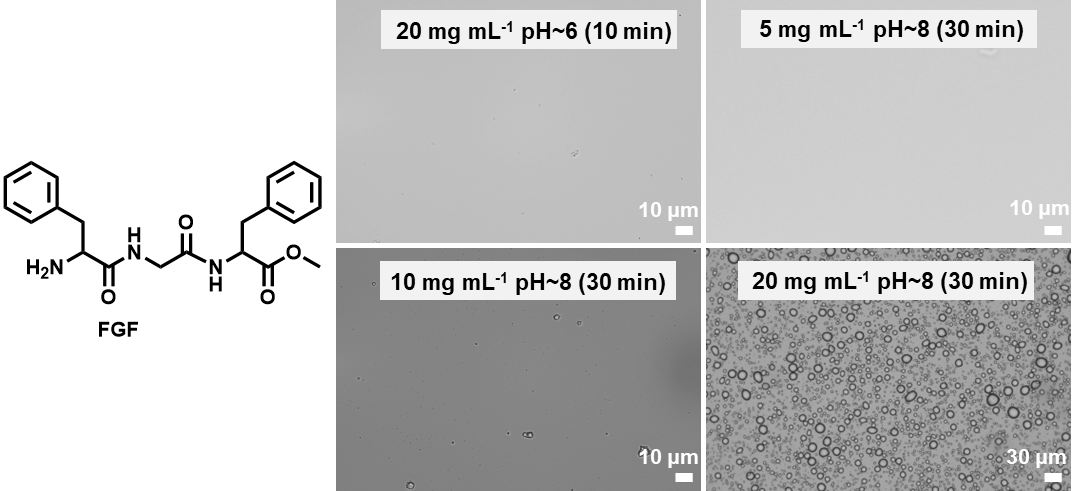


**Figure S27**. Left: Chemical structure of the tripeptide FGF; Right: FGF dissolved at pH ~6 (in 5 mM Hepes buffer) and exhibits concentration-dependent assembly behavior at pH ~8, forming coacervates at high concentrations (20 mg mL^-1^) in 30 minutes. Scale bar = 10 μm in the microscopy image. Similar results were obtained with 3 samples measured independently.


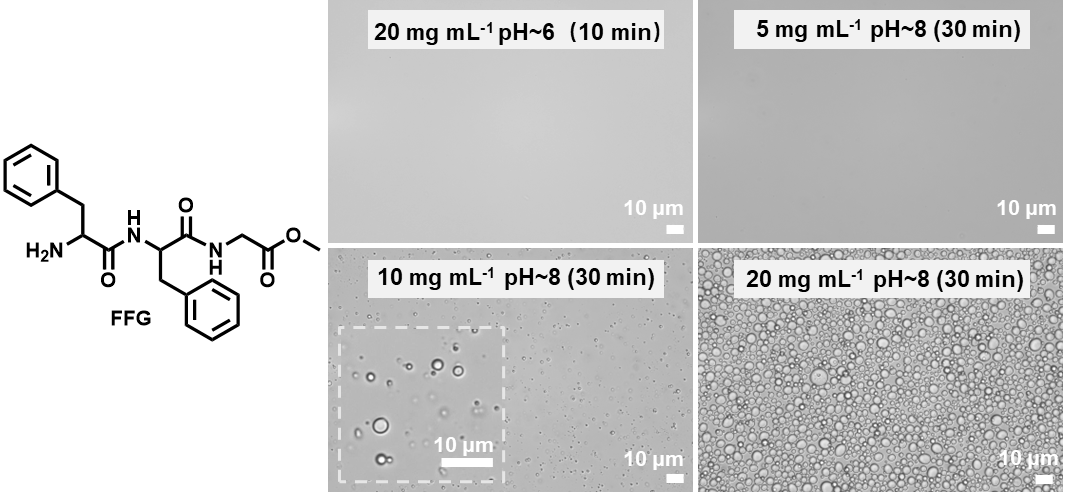


**Figure S28**. Left: Chemical structure of the tripeptide FFG; Right: FFG dissolved at pH ~6 (in 5 mM Hepes buffer) and exhibits concentration-dependent assembly behavior at pH ~8, forming coacervates at high concentrations (20 mg mL^-1^) in 30 minutes. Scale bar = 10 μm in the microscopy image. Similar results were obtained with 3 samples measured independently.

###
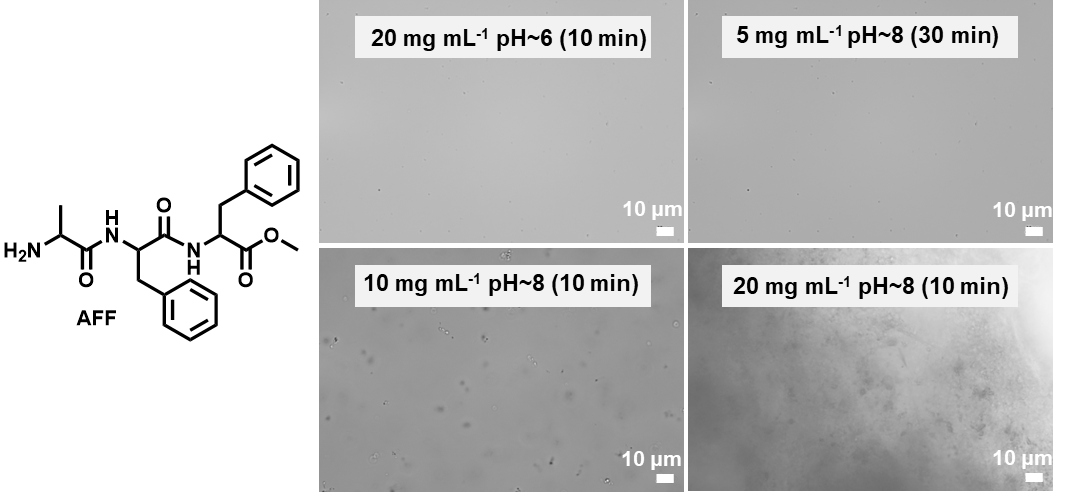


**Figure S29.** Left: Chemical structure of the tripeptide AFF; Right: AFF dissolved at pH ~6 (in 5 mM Hepes buffer) and exhibits concentration-dependent assembly behavior at pH ~8, forming aggregates at high concentrations (20 mg mL^-1^) in 10 minutes. Scale bar = 10 μm in the microscopy image. Similar results were obtained with 3 samples measured independently.

###
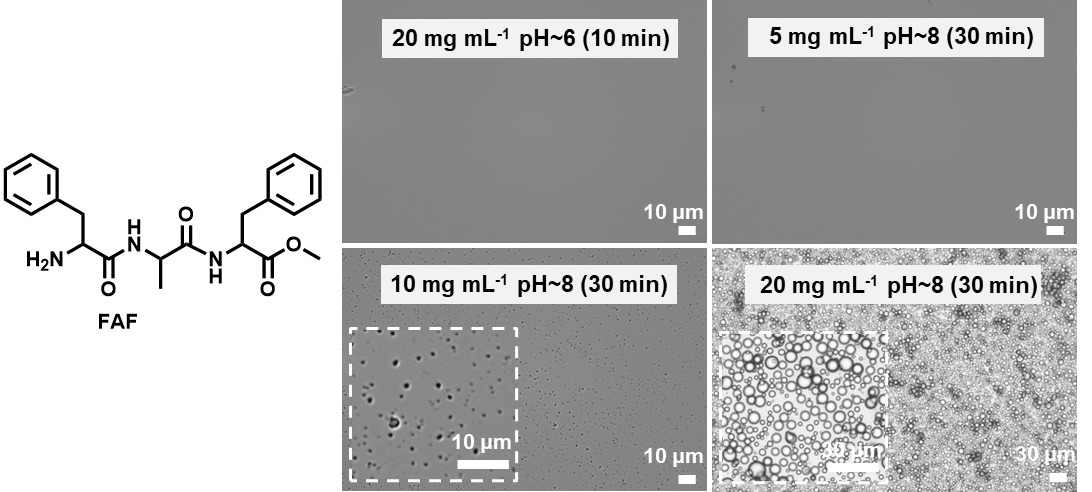


**Figure S30**. Left: Chemical structure of the tripeptide FAF; Right: FAF dissolved at pH ~6 (in 5 mM Hepes buffer) and exhibits concentration-dependent assembly behavior at pH ~8, forming coacervates at high concentrations (20 mg mL^-1^) in 30 minutes. Scale bar = 10, 30 μm in the microscopy image. Similar results were obtained with 3 samples measured independently.

###
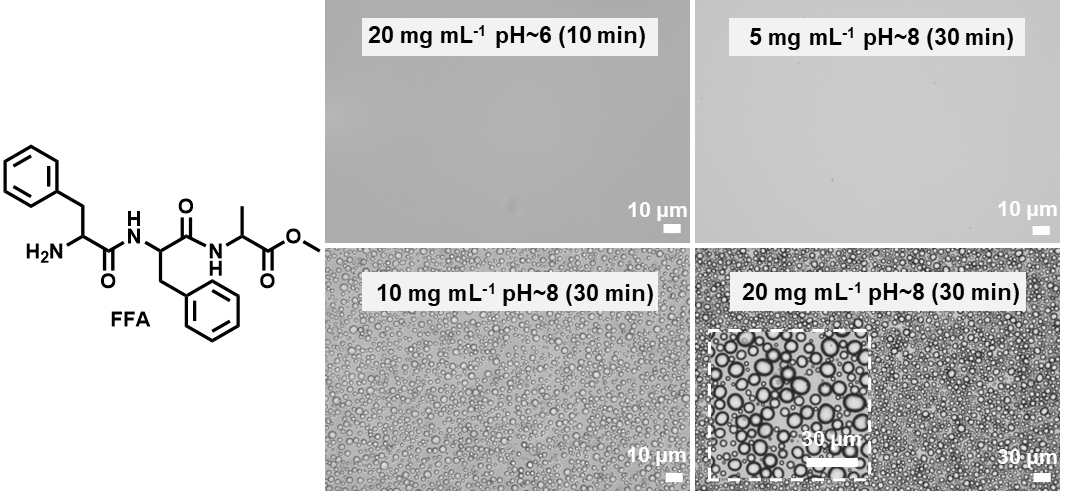


**Figure S31**. Left: Chemical structure of the tripeptide FFA; Right: FFA dissolved at pH ~6 (in 5 mM Hepes buffer) and exhibits concentration-dependent assembly behavior at pH ~8, forming coacervates at high concentrations (20 mg mL^-1^) in 30 minutes. Scale bar = 10 or 30 μm in the microscopy image. Similar results were obtained with 3 samples measured independently.

###
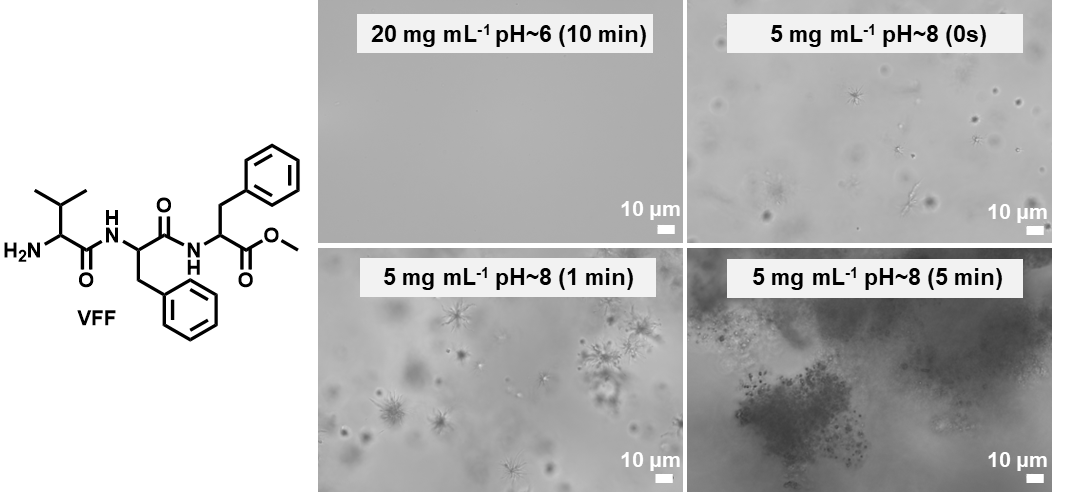


**Figure S32**. Left: Chemical structure of the tripeptide VFF; Right: VFF dissolved at pH ~ 6 (in 5 mM Hepes buffer) and showing the rapid transition of VFF from small aggregates to large aggregates under conditions of 5 mg mL^-1^ at pH ~8 in 5 minutes. Scale bar = 10 μm in the microscopy image. Similar results were obtained with 3 samples measured independently.

###
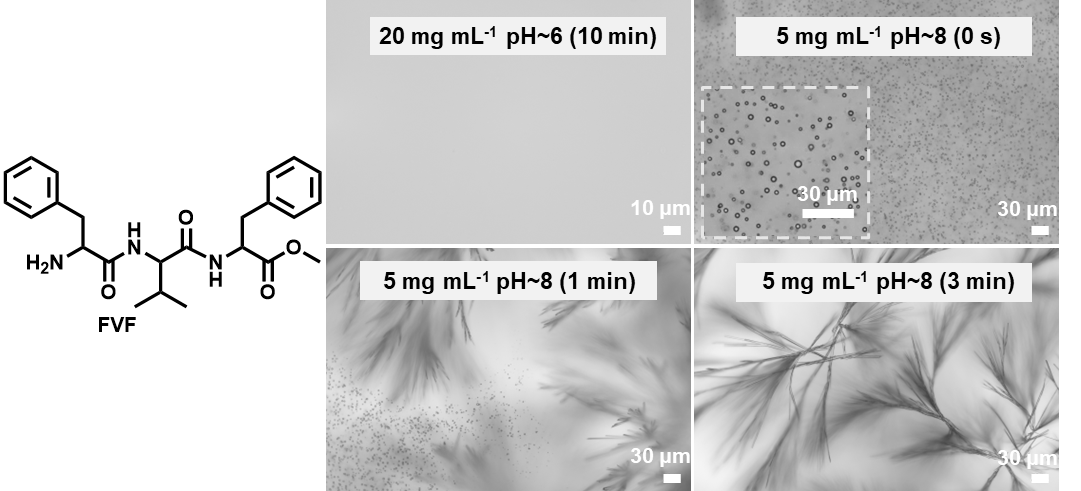


**Figure S33**. Left: Chemical structure of the tripeptide FVF; Right: FVF dissolved at pH ~6 (in 5 mM Hepes buffer) and shows the rapid transition of FVF from coacervates to long fiber-like morphology under conditions of 5 mg mL^-1^ at pH ~8 in 3 minutes. Scale bar = 10, 30 μm in the microscopy image. Similar results were obtained with 3 samples measured independently.


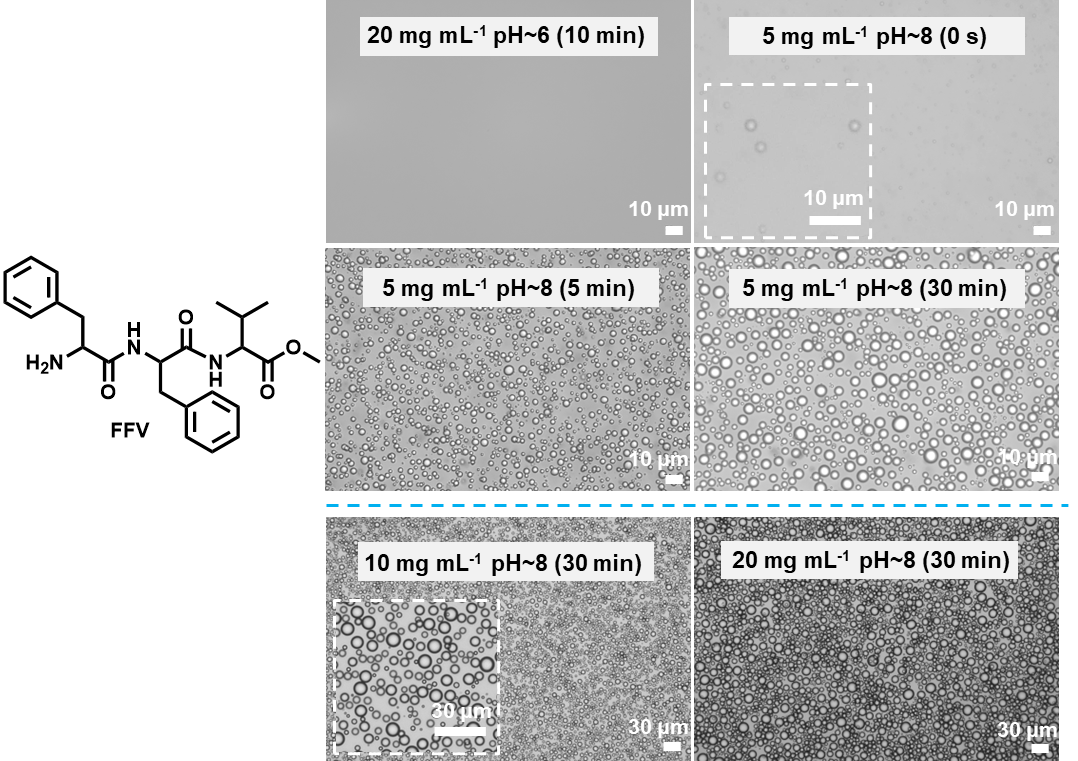


**Figure S34**. Left: Chemical structure of the tripeptide FFV. Right TOP: FFV dissolved at pH ~6 (in 5 mM Hepes buffer) and forms stable Coacervates at 5 mg mL^-1^ and pH ~8 in 30 minutes. Right DOWN: Microscopic images of FFV showed fine coacervates at different peptide concentrations and pH ~8. Scale bar = 10, 30 μm in the microscopy image. Similar results were obtained with 3 samples measured independently.

###
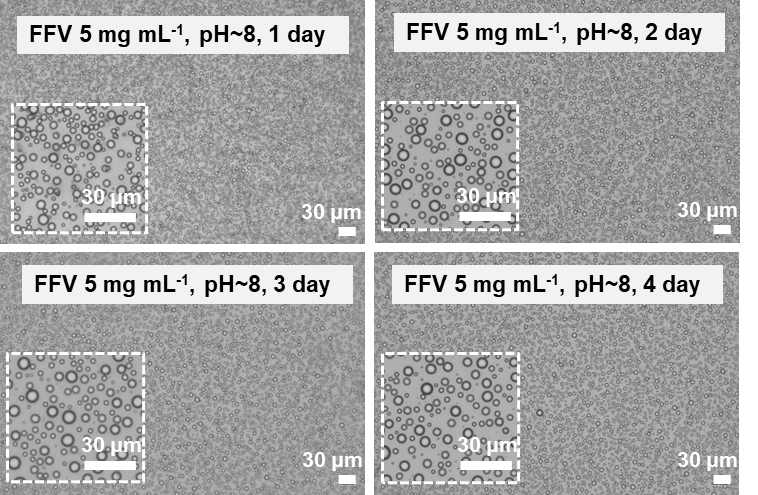


**Figure S35**. Stability of FFV coacervates (5 mg mL^-1^, 5 mM Hepes buffer pH~8) after incubation for 4 days. Scale bar = 30 μm in all microscopy images.

###
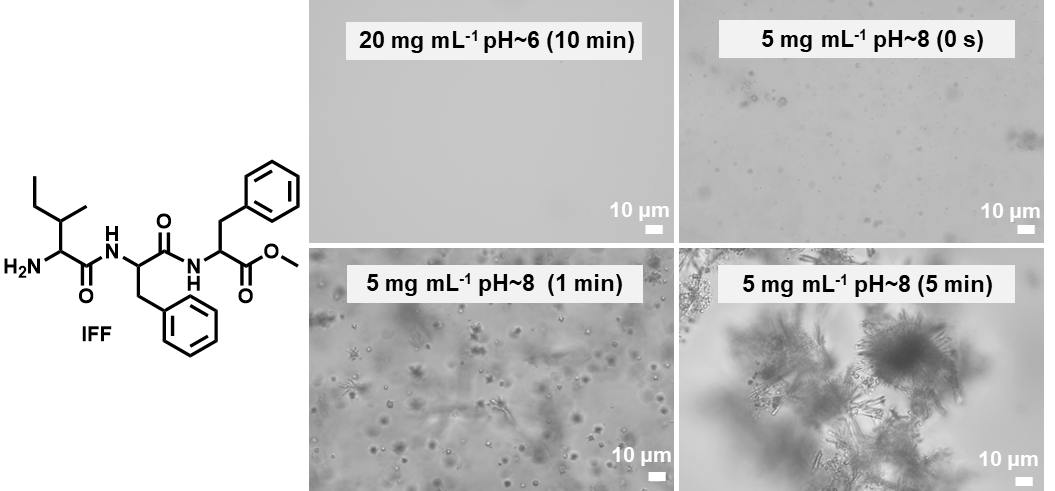


**Figure S36**. Left: Chemical structure of the tripeptide IFF; Right: IFF dissolved at pH ~6 (in 5 mM Hepes buffer) and shows the rapid transition of IFF from small aggregates to large aggregates under conditions of 5 mg mL^-1^ at pH ~8 in 5 minutes. Scale bar = 10 μm in the microscopy image. Similar results were obtained with 3 samples measured independently.


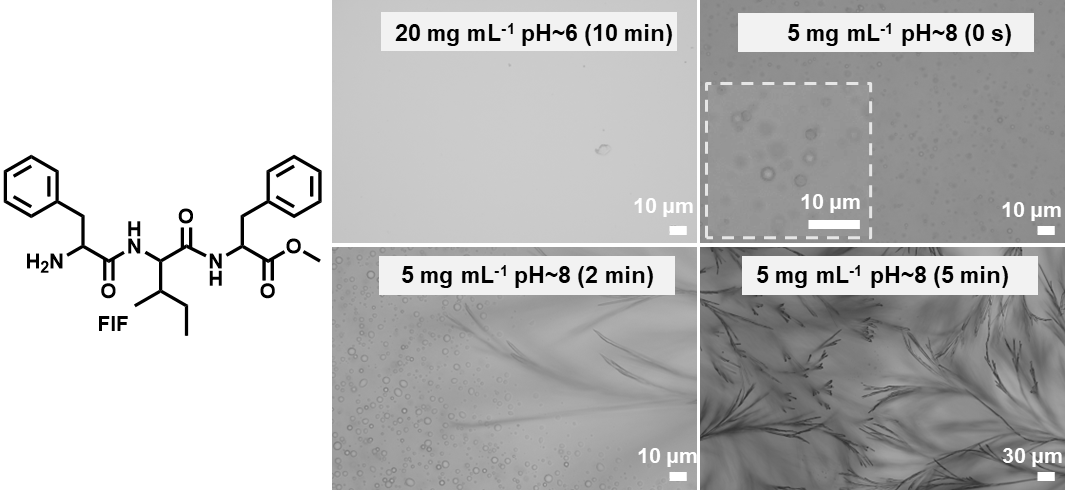


**Figure S37**. Left: Chemical structure of the tripeptide FIF; Right: FIF dissolved at pH ~6 (in 5 mM Hepes buffer) and shows the rapid transition of FIF from coacervates to long fiber-like morphology under conditions of 5 mg mL^-1^ at pH ~8 in 5 minutes. Scale bar = 10, 30 μm in the microscopy image. Similar results were obtained with 3 samples measured independently.

###
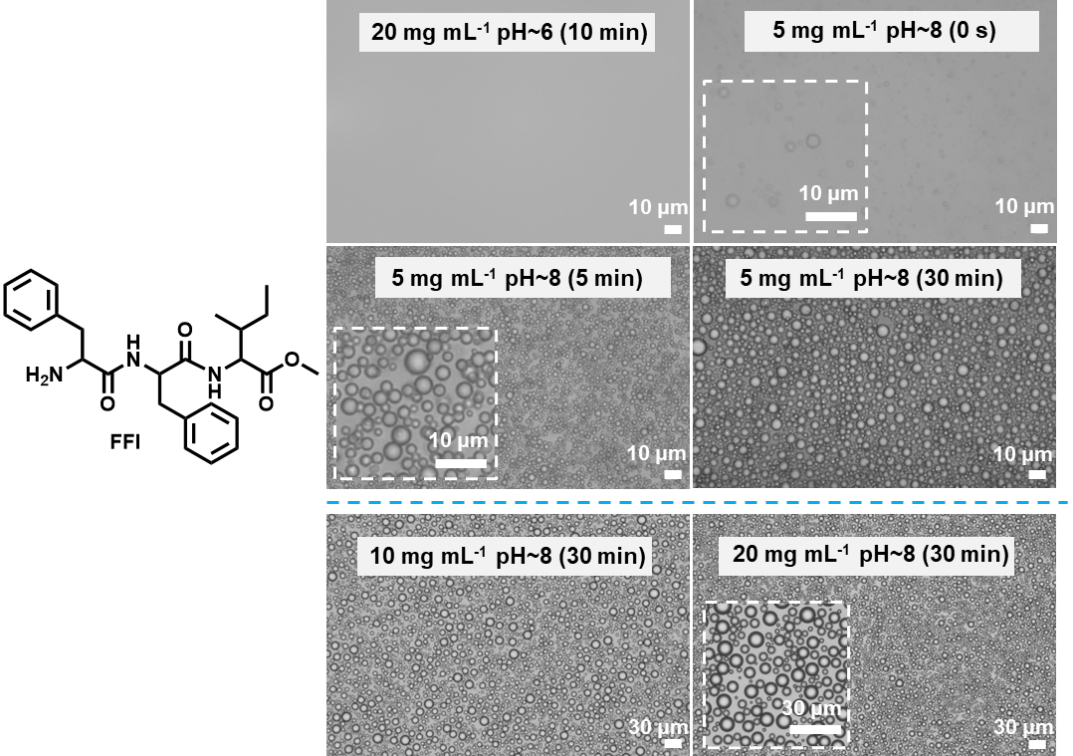


**Figure S38**. Left: Chemical structure of the tripeptide FFI. Right TOP: FFI dissolved at pH ~6 (in 5 mM Hepes buffer) and forms stable coacervates at 5 mg mL^-1^ and pH ~8 in 30 minutes. Right DOWN: Microscopic images of FFI showed fine coacervates at different peptide concentrations and pH ~8. Scale bar = 10 or 30 μm in the microscopy image. Similar results were obtained with 3 samples measured independently.

###
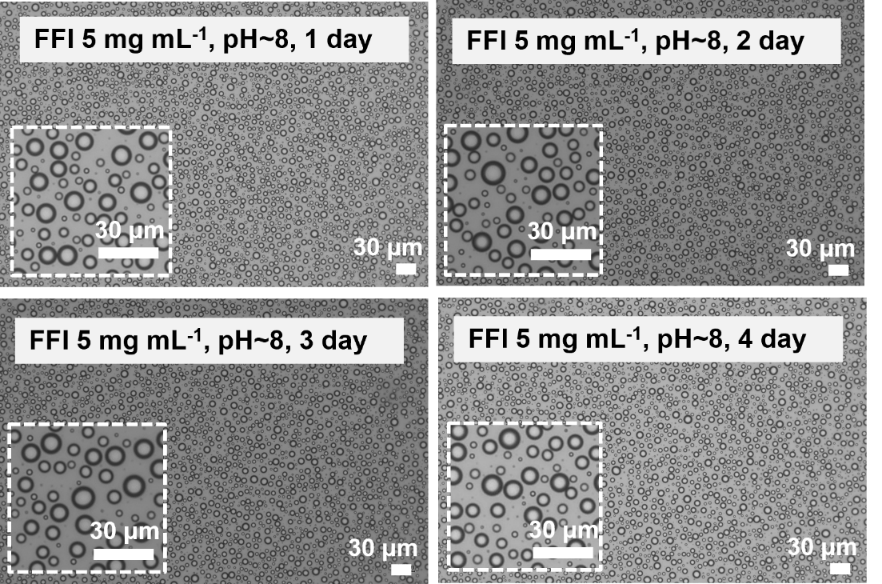


**Figure S39**. Stability of FFI coacervates (5 mg mL^-1^, 5 mM Hepes buffer pH~8) during the incubation for 4 days. Scale bar = 30 μm in all microscopy images.

###
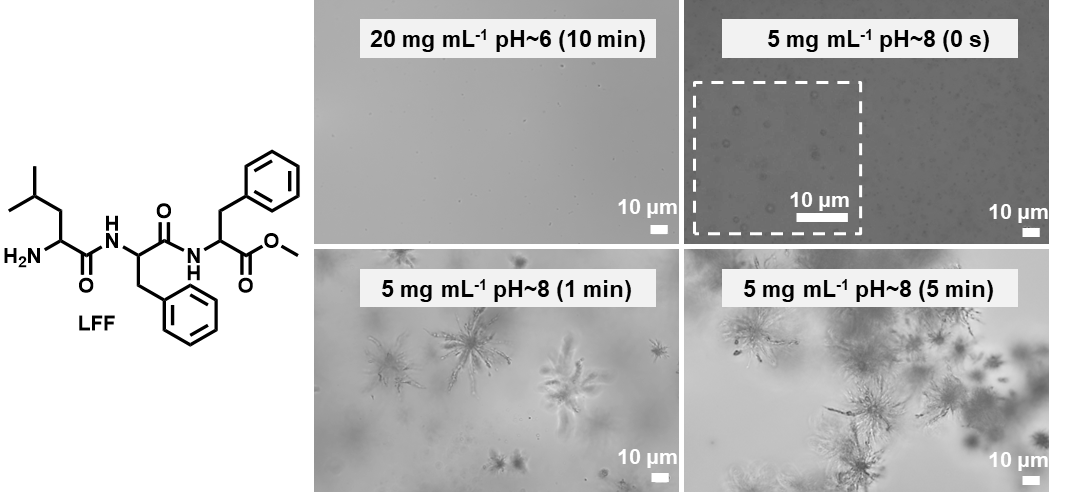


**Figure S40**. Left: Chemical structure of the tripeptide LFF; Right: LFF dissolved at pH ~6 (in 5 mM Hepes buffer) and shows the rapid transition of LFF from small coacervates to large aggregates under conditions of 5 mg mL^-1^ at pH ~8 in 5 minutes. Scale bar = 10 μm in the microscopy image. Similar results were obtained with 3 samples measured independently.

###
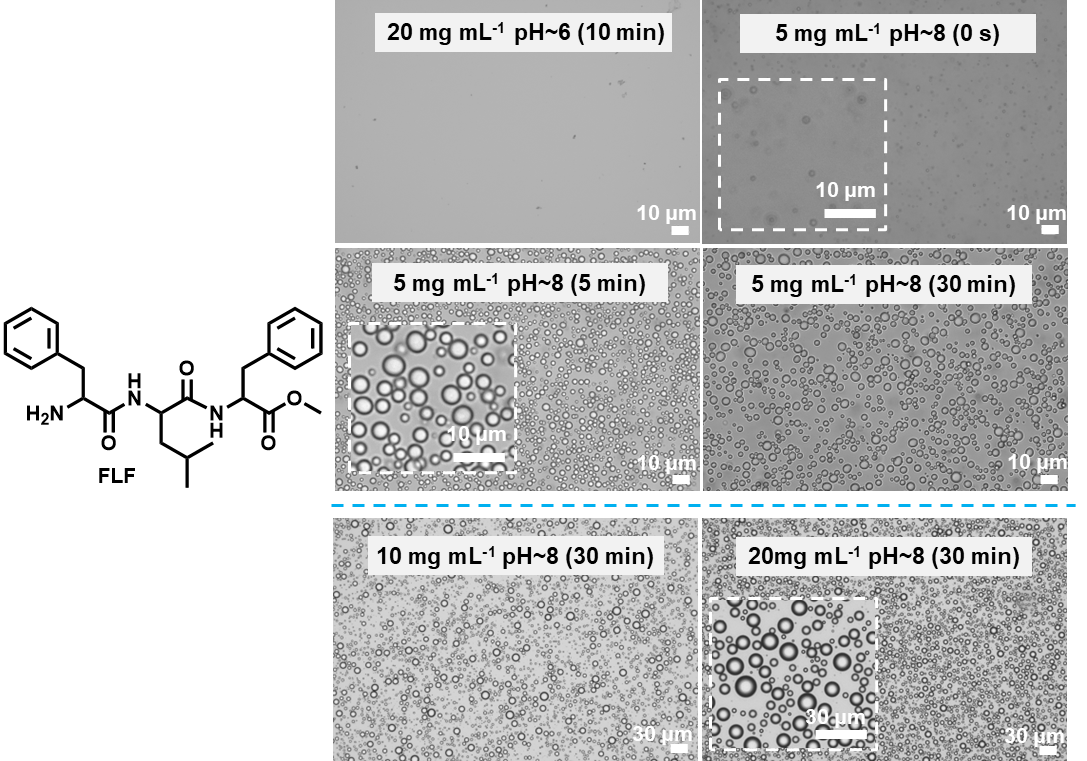


**Figure S41**. Left: Chemical structure of the tripeptide FLF. Right TOP: FLF dissolved at pH ~6 (in 5 mM Hepes buffer) and form stable coacervates at 5 mg mL^-1^ and pH ~8 in 30 minutes. Right DOWN: Microscopic images of FLF showed fine coacervates at different peptide concentrations and pH ~8. Scale bar = 10 or 30 μm in the microscopy image. Similar results were obtained with 3 samples measured independently.

###
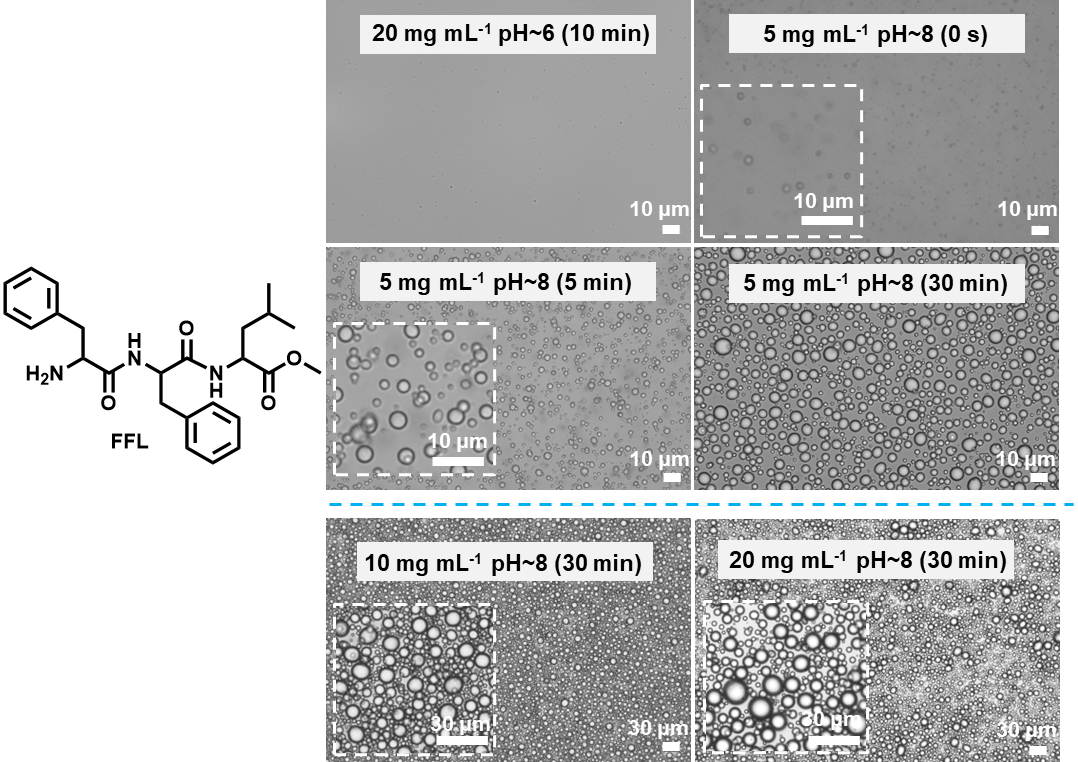


**Figure S42**. Left: Chemical structure of the tripeptide FFL. Right TOP: FFL dissolved at pH ~6 (in 5 mM Hepes buffer) and form stable coacervates at 5 mg mL^-1^ and pH ~8 in 30 minutes. Right DOWN: Microscopic images of FFL showed fine coacervates at different peptide concentrations and pH ~8. Scale bar = 10 or 30 μm in the microscopy image. Similar results were obtained with 3 samples measured independently.

###
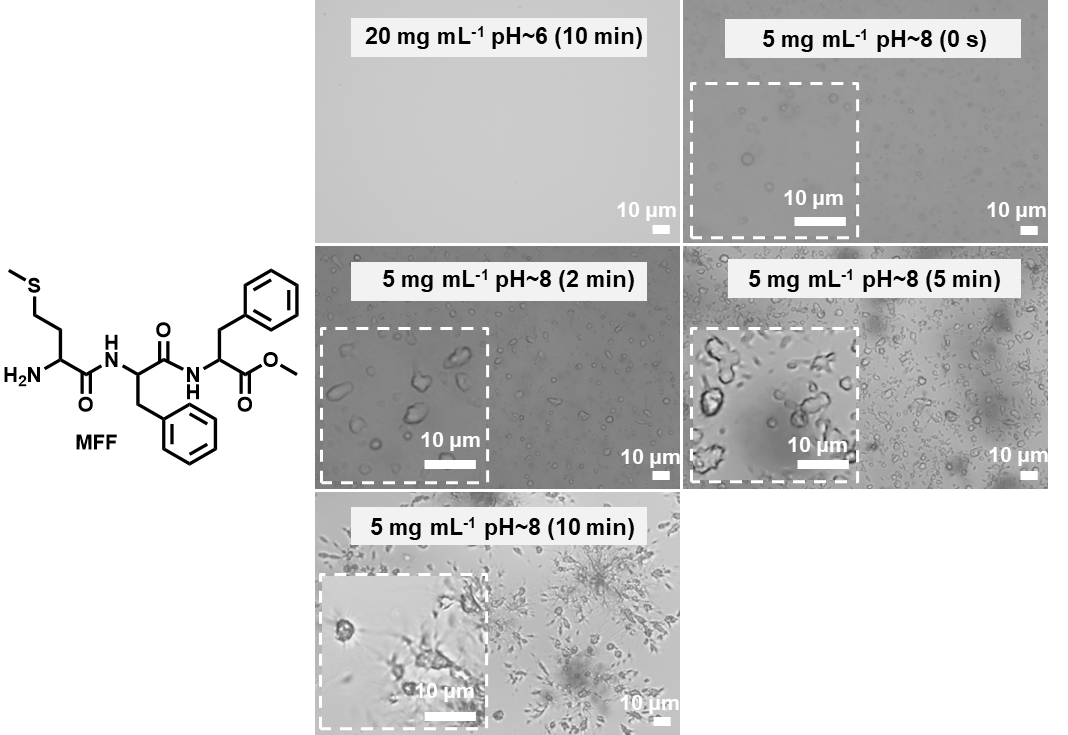


**Figure S43**. Left: Chemical structure of the tripeptide MFF; Right: MFF dissolved at pH ~6 (in 5 mM Hepes buffer) and shows the rapid transition of MFF from small coacervates to large aggregates under conditions of 5 mg mL^-1^ at pH ~8 in 10 minutes. Scale bar = 10 μm in the microscopy image. Similar results were obtained with 3 samples measured independently.

###
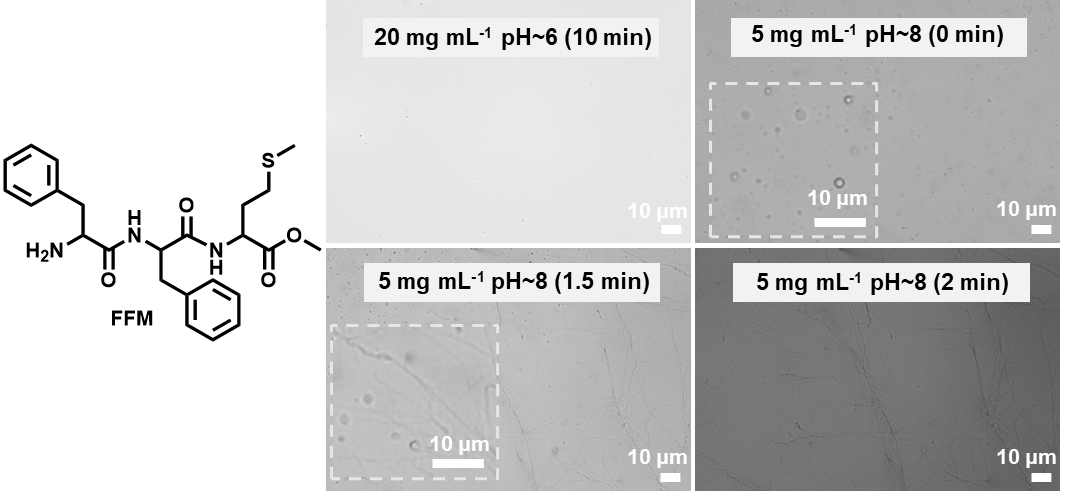


**Figure S44**. Left: Chemical structure of the tripeptide FFM; Right: FFM dissolved at pH ~6 (in 5 mM Hepes buffer) and shows the rapid transition of FFM from small coacervates to long fiber-like morphology under conditions of 5 mg mL^-1^ at pH ~8 in 2 minutes. Scale bar = 10 μm in the microscopy image. Similar results were obtained with 3 samples measured independently.

###
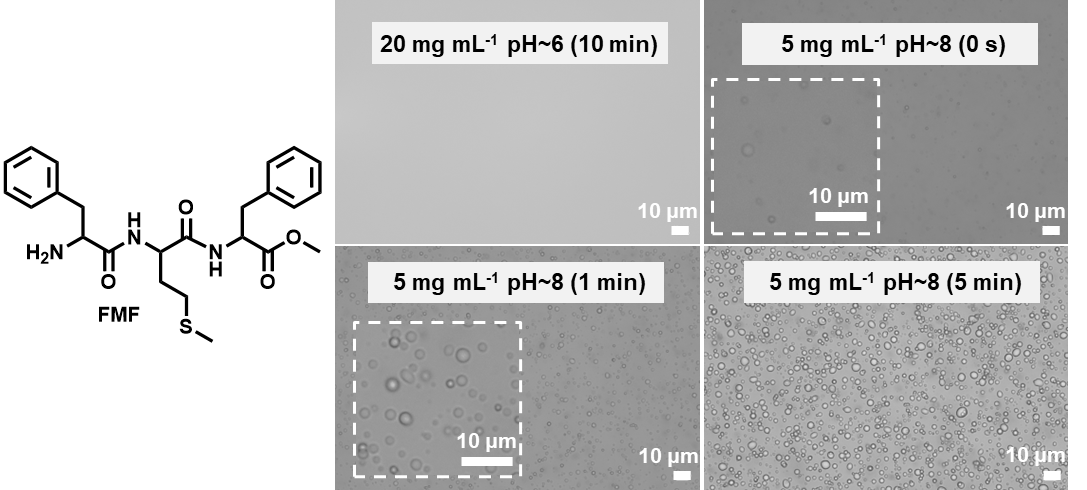


**Figure S45**. Left: Chemical structure of the tripeptide FMF; Right: FMF dissolved at pH ~6 (in 5 mM Hepes buffer) and forms stable coacervates at 5 mg mL^-1^ and pH ~8 in 5 minutes. Scale bar = 10 μm in the microscopy image. Similar results were obtained with 3 samples measured independently.

###
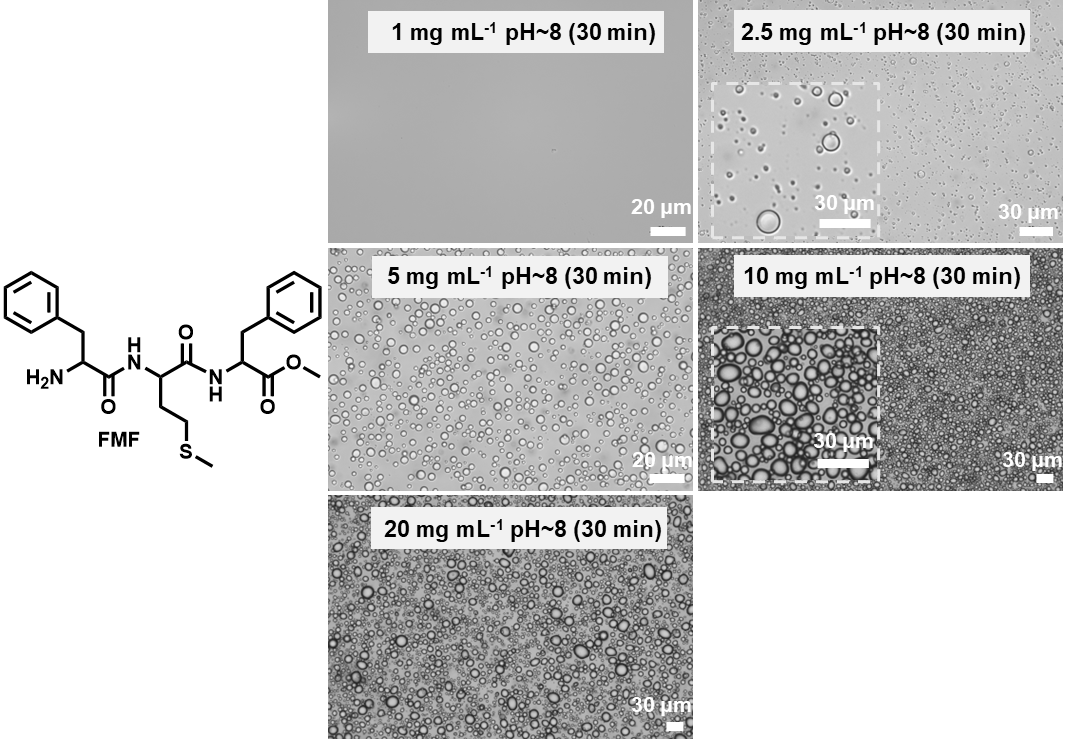


**Figure S46**. Left: Chemical structure of the tripeptide FMF; Right: Concentration-dependent phase separation of FMF at pH ~8 (in 5 mM Hepes buffer). Based on the microscopy imaging, the threshold concentration for FMF coacervate formation was approximately between 1 and 2.5 mg mL^-1^. Scale bar = 20 or 30 μm in all microscopy images. Similar results were obtained with 3 samples measured independently.

###
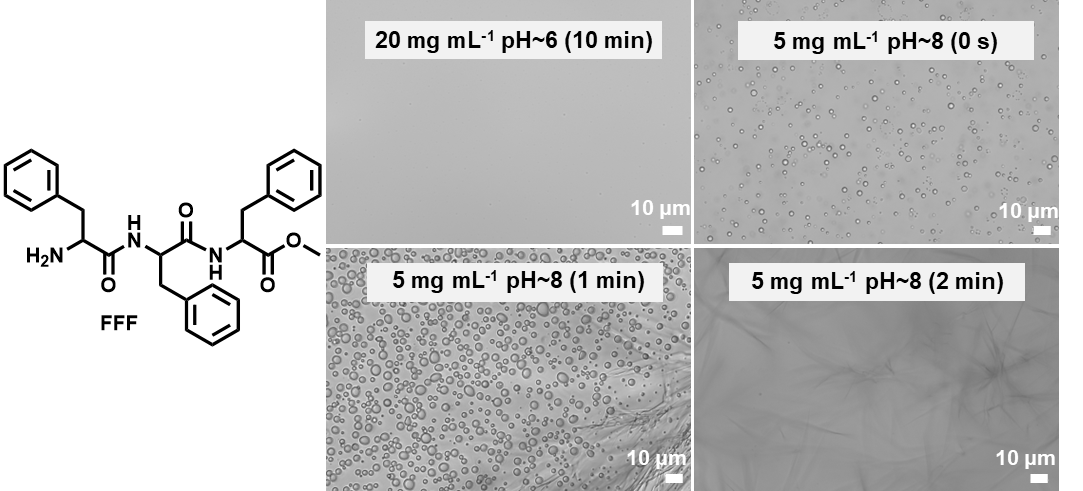


**Figure S47**. Left: Chemical structure of the tripeptide FFF; Right: FFF dissolved at pH ~6 (in 5 mM Hepes buffer) and shows the rapid transition of FFF from small coacervates to long fiber-like morphology under conditions of 5 mg mL^-1^ at pH ~8 in 2 minutes. Scale bar = 10 μm in the microscopy image. Similar results were obtained with 3 samples measured independently.

###
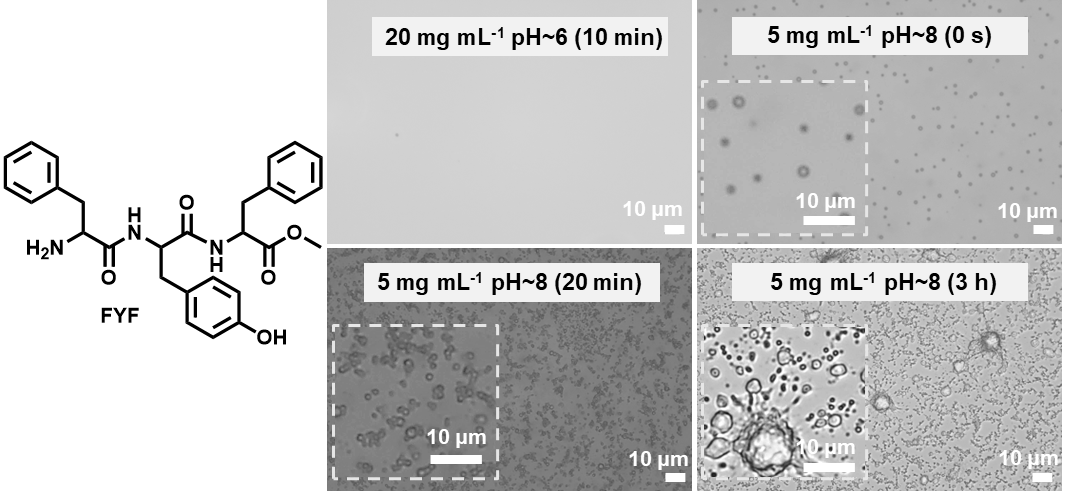


**Figure S48**. Left: Chemical structure of the tripeptide FYF; Right: FYF dissolved at pH ~6 (in 5 mM Hepes buffer) and shows the rapid transition of FYF from small aggregates to large aggregates under conditions of 5 mg mL^-1^ at pH ~8 in 3h. Scale bar = 10 μm in the microscopy image. Similar results were obtained with 3 samples measured independently.

###
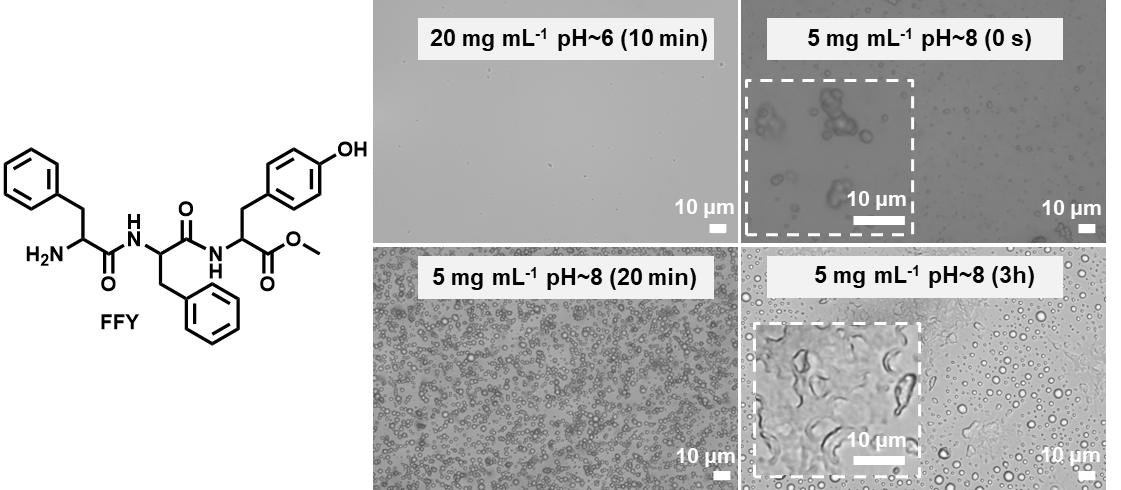


**Figure S49**. Left: Chemical structure of the tripeptide FFY; Right: FFY dissolved at pH ~6 (in 5 mM Hepes buffer) and showed the rapid transition of FFY from small aggregates to large aggregates under conditions of 5 mg mL^-1^ at pH ~8 in 3h. Scale bar = 10 μm in the microscopy image. Similar results were obtained with 3 samples measured independently.

###
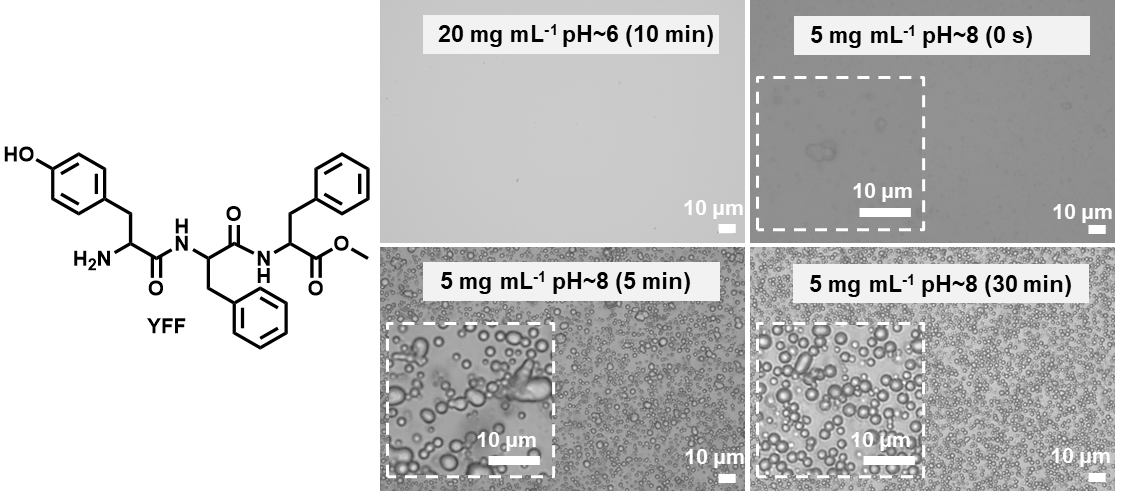


**Figure S50**. Left: Chemical structure of the tripeptide YFF; Right: YFF dissolved at pH ~6 (in 5 mM Hepes buffer) and rapidly formed aggregates-like morphology at pH ~8 in 30 minutes. Scale bar = 10 μm in the microscopy image. Similar results were obtained with 3 samples measured independently.

**Fluorescence Recovery After Photobleaching (FRAP) of YFF (5 mg mL^-1^, pH ~8, 0.001 mg mL^-1^ Nile red)**

###
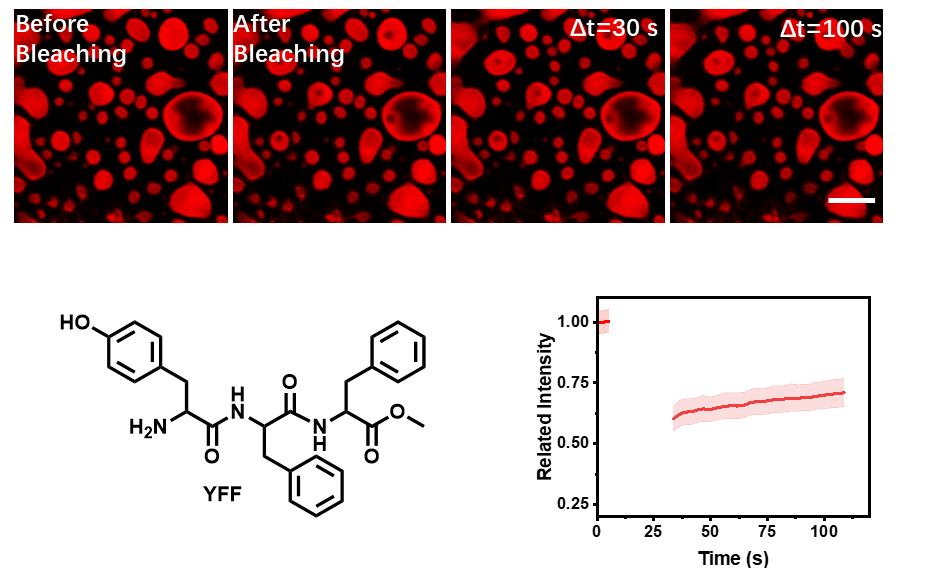


**Figure S51**. Top: Confocal images corresponding to FRAP in YFF aggregates (5 mg mL^-1^) over time, the red emission is Nile Red. Scale bar = 10 μm. Down: FRAP traces of YFF aggregates (5 mg mL^-1^) over time. Fluorescence recovery is slow, indicating that aggregates are less dynamic. Similar results were obtained with 3 samples measured independently. Data represent mean ± SD for n = 5 representative microscopic images.

## 8. **FMF tripeptide coacervates and active condensates microreactors**

###
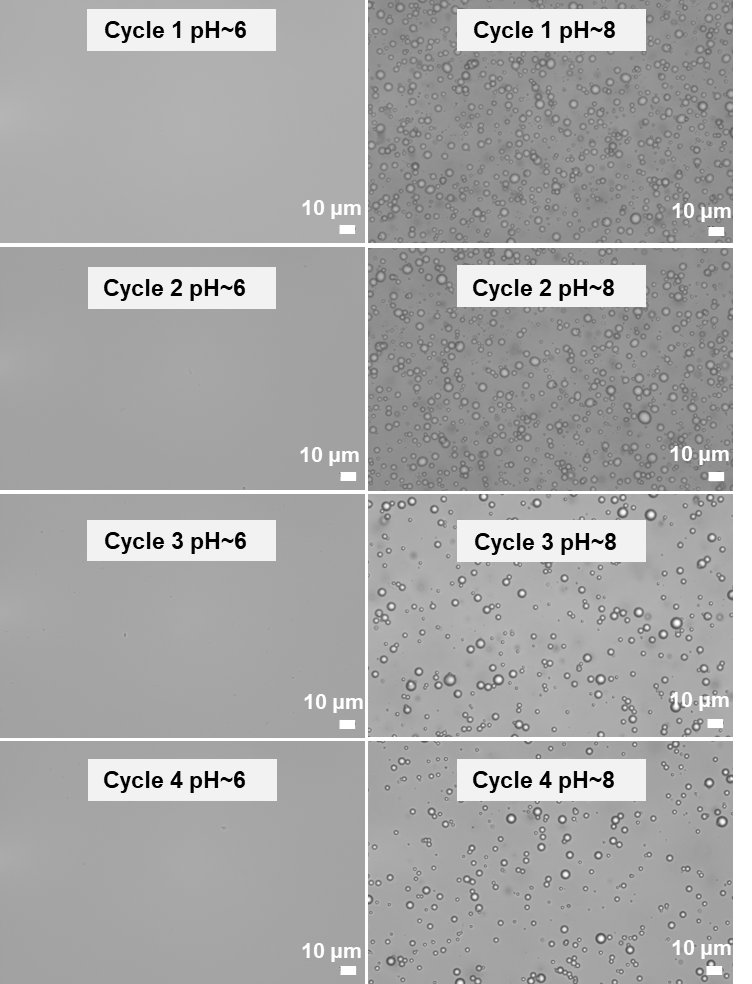


**Figure S52**. pH reversible phase separation of FMF. Initially, the 5 mg mL^-1^ FMF peptides are solubilized in pH 6 (in 5 mM Hepes buffer). Increasing the pH to ~8 induces the formation of liquid droplets. The solubilization and formation of FMF coacervates can be repeated several times by sequentially decreasing or increasing the pH by adding a concentrated HCl or NaOH solution, scale bar=10 μm in all images.

###
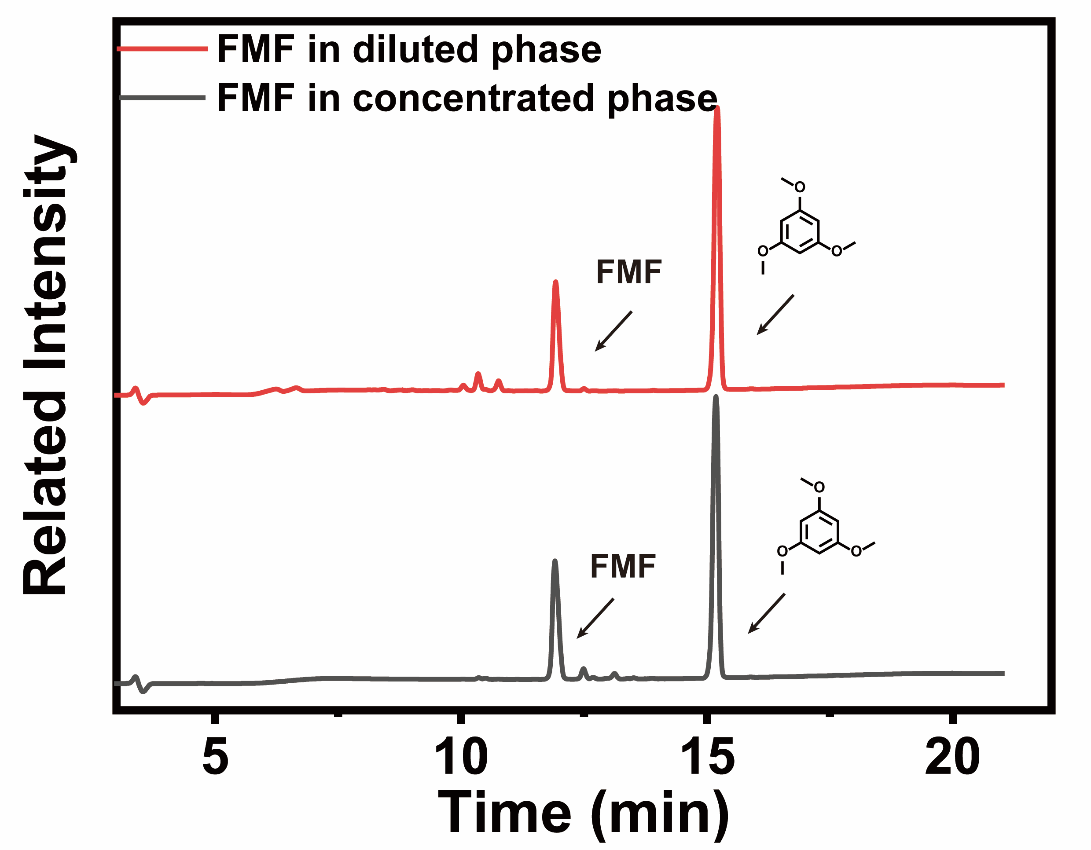


**Figure S53**. HPLC analysis of relative peptide content in the dilute phase and concentrated phase for FMF coacervates. Phase separation was achieved by low-speed centrifugation, followed by redissolution in a MeCN/5 mM Hepes buffer (1:1, v/v) mixture. Each phase was analyzed using 1,3,5 trimethoxybenzene as an external standard. Integration of the peptide peaks indicated that the ratio of total peptide content in the concentrated phase compared to the diluted phase was approximately 1.14:1.

###
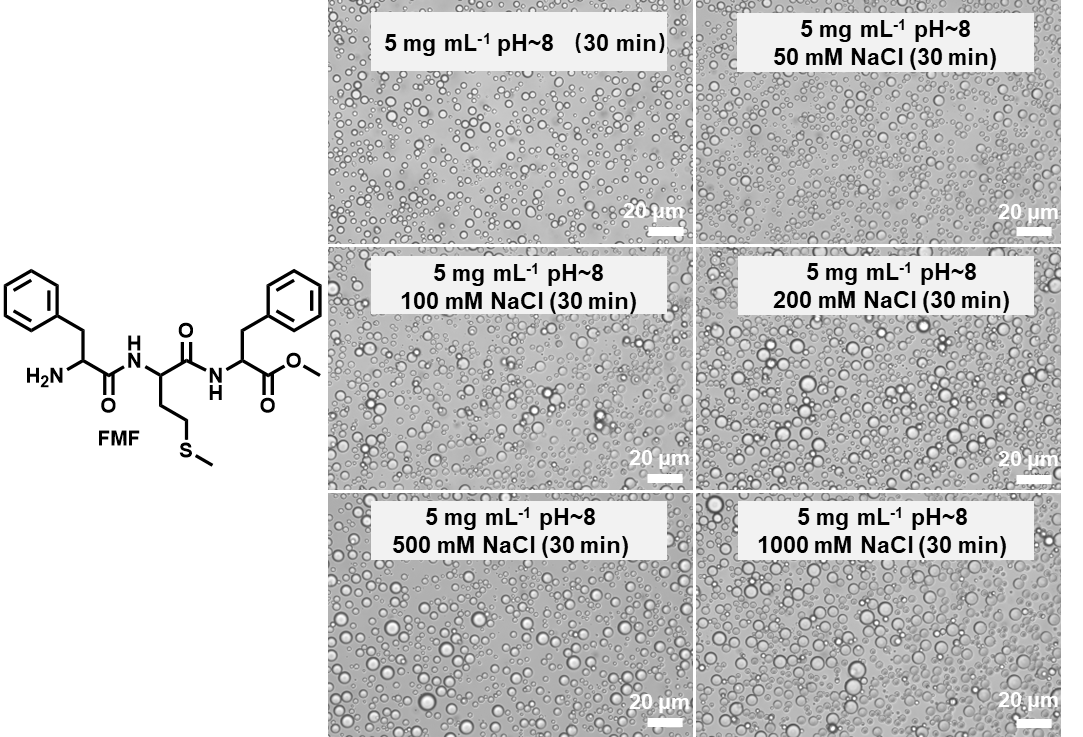


**Figure S54**. Left: Chemical structure of the tripeptide FMF; Right: Microscopic images of FMF at pH 8 (in 5 mM Hepes buffer) showed LLPS behaviors at 5 mg mL^-1^ and different NaCl concentrations. Scale bar=20 μm in the microscopy image. Similar results were obtained with 3 samples measured independently.

###
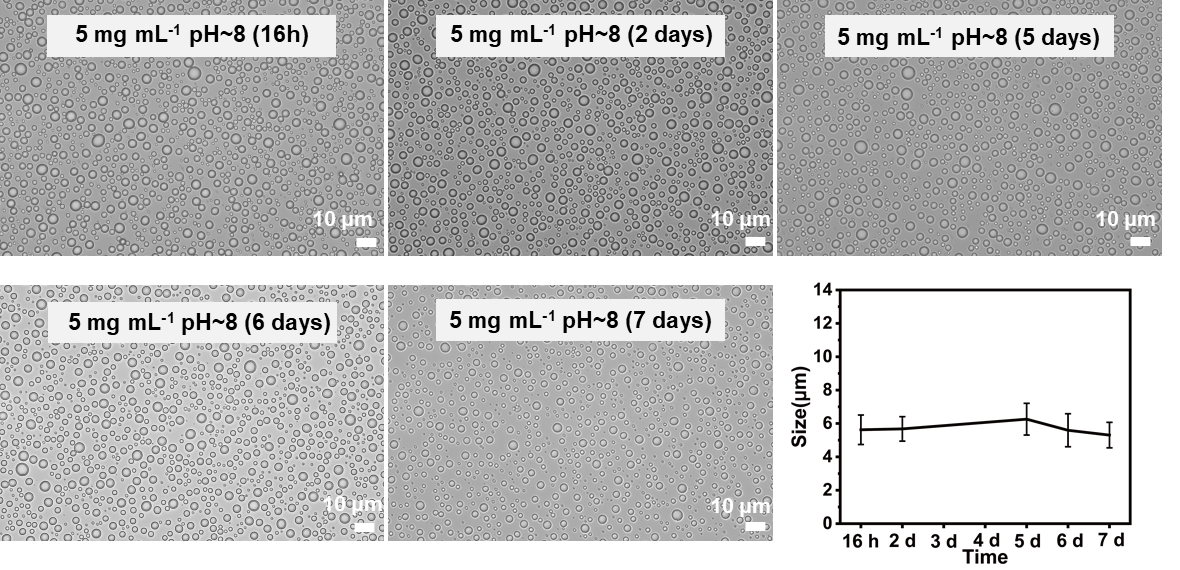


**Figure S55**. Stability of FMF coacervates (5 mg mL^-1^, 5 mM Hepes buffer pH~8) during the incubation for 7 days. Scale bar = 10 μm in all microscopy images.

###
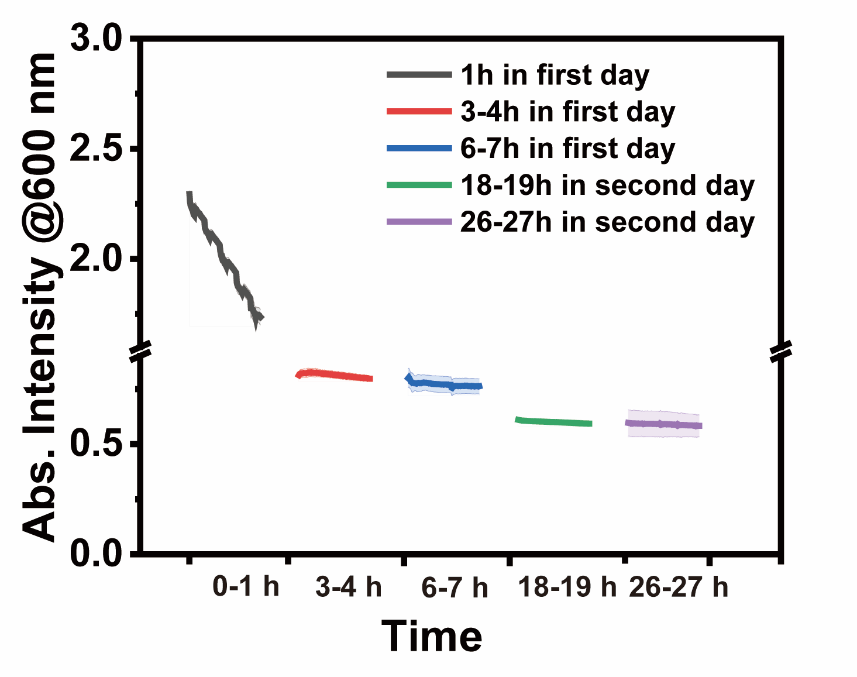


**Figure S56**. Turbidity change of FMF coacervate solution (5 mg mL^-1^ peptides, 5 mM Hepes buffer, pH~8) measured over two days incubation.

**Fluorescence Recovery After Photobleaching (FRAP) of MFF (5 mg ml^-1^, pH ~ 8, 0.001 mg mL^-1^ Nile red)**

###
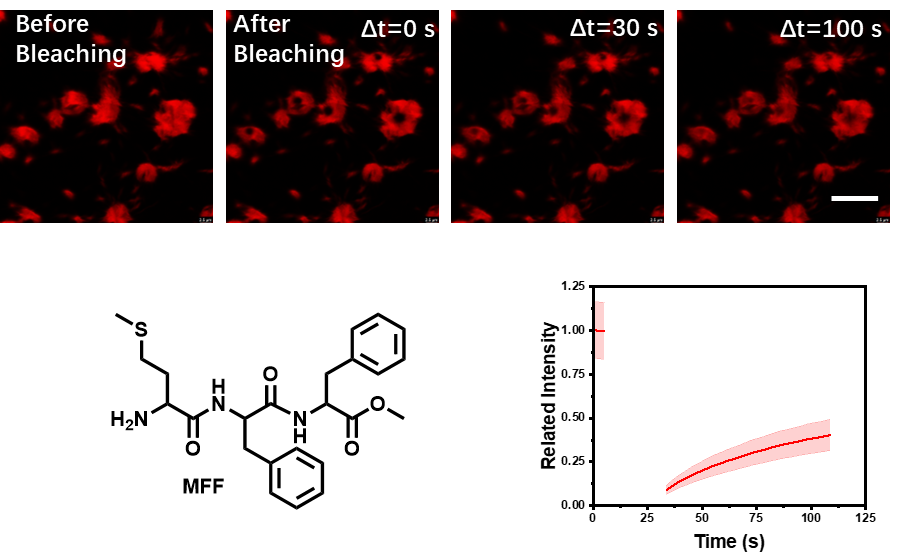


**Figure S57**. Top: Confocal images corresponding to FRAP in MFF aggregates (5 mg mL^-1^) over time, the red emission is Nile Red. Scale bar = 10 μm. Down: FRAP traces of MFF aggregates (5 mg mL^-1^) over time. Fluorescence recovery is slow, indicating that aggregates are less dynamic. Similar results were obtained with 3 samples measured independently. Data represent mean ± SD for n = 5 representative microscopic images.

###
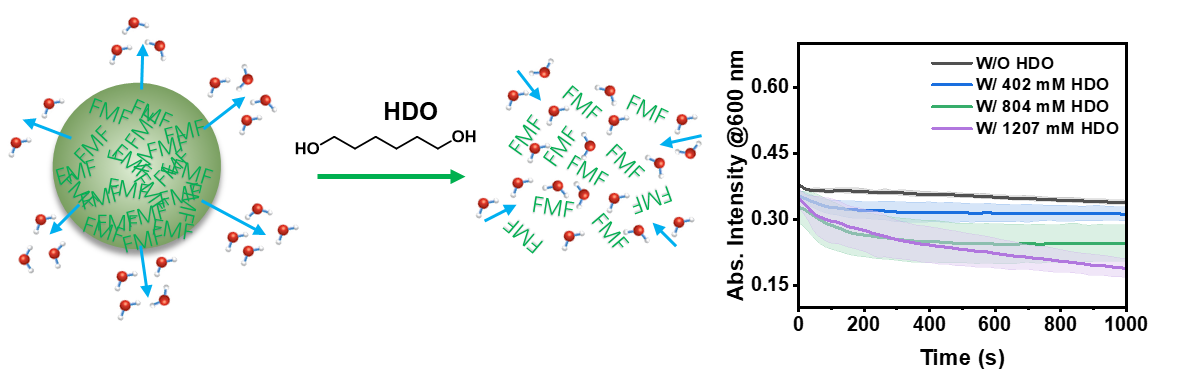


###
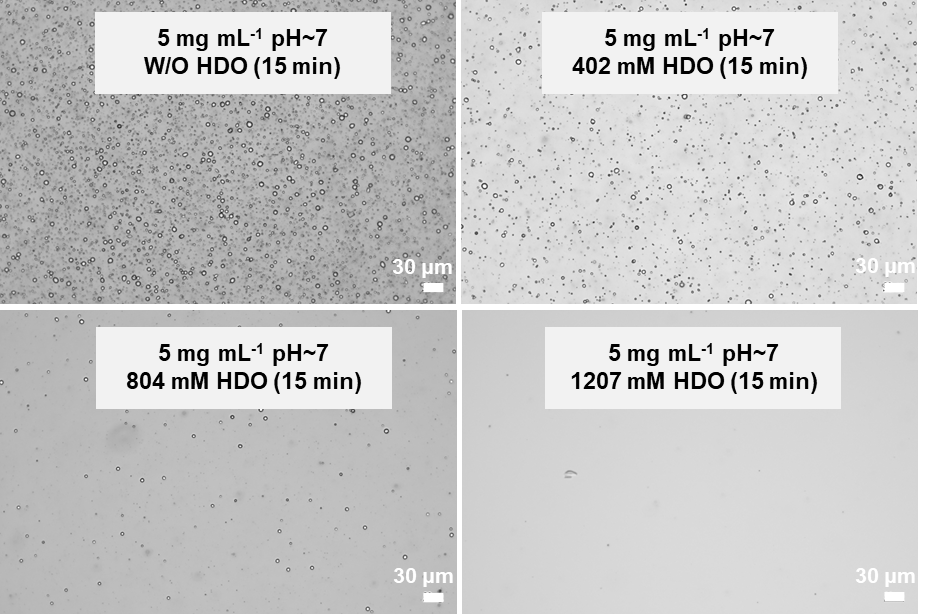


**Figure S58**. The phase separation behavior of FMF peptide was affected by the presence of HDO (1, 6-Hexanediol). The FMF coacervates (5 mg mL^-1^, 10.9 mM, 5 mM Hepes buffer, pH ~7) were able to maintain their structure up to 804 mM HDO. The presence of 1207 mM HDO induced the solvation of the coacervates. Similar results were obtained with 3 samples measured independently. Scale bar = 30 μm.

###

**Figure S59**. The phase separation behavior of FMF peptide was affected by the presence of Urea. The FMF coacervates (5 mg mL^-1^, 10.9 mM, 5 mM Hepes buffer, pH ~7) were able to maintain their structure up to 3M Urea. The presence of 5 M Urea induced the solvation of the coacervates. Similar results were obtained with 3 samples measured independently. Scale bar = 30 μm.

###

**Figure S60**. The phase separation behavior of FMF peptide was affected by the presence of H_2_O_2_. The FMF coacervates (5 mg mL^-1^, 10.9 mM, 5 mM Hepes buffer, pH ~7) were able to maintain their structure when fed with 32.6 mM H_2_O_2_. The presence of 332 mM H_2_O_2_ induced the solvation of the coacervates in 10 minutes. Similar results were obtained with 3 samples measured independently. Scale bar = 30 μm.

###

**Figure S61**. (a) Scheme illustration of the FMF oxidation into sulfoxide or sulfone product. (b) HPLC measurements of FMF (5 mg mL^-1^, 10.9 mM, in 5 mM Hepes buffer, pH~8) incubated with 332 mM H_2_O_2_ over 10 min. A decrease in the FMF peak is observed, accompanied by the emergence of a new peak as evidence of generation of FMF oxidized products.

###

**Figure S62**. Turbidity changes of FMF coacervates (5 mg mL^-1^, 10.9 mM, 5 mM Hepes buffer) mixed with 0.02 mg mL^-1^ urease when fed with different concentrations of urea. Data represent mean ± SD for n = 3 independent samples. Error bars depict the standard deviation (SD) obtained from microplate reader analysis.

###

**Figure S63**. Reversible control of FMF coacervate generation and disappearance using urease/urea (1 μmol urea every time) and hydrochloric acid (0.5 μmol HCl every time) and corresponding photos of the bright field. Scale bar = 10 μm. Data represent mean ± SD for n = 3 independent samples. Error bars depict the standard deviation (SD) obtained from microplate reader analysis.

###

**Figure S64**. (a) HPLC measurements of FMF (5 mg mL^-1^ in 5 mM Hepes buffer, 10.9 mM, pH~8) incubated with 0.2 g L^-1^ GOx and 30 mM glucose over 40 min. Bare change of FMF peak was found during the experiment. (b) The change of pH was monitored over time using the same condition in (a). Based on the results, the reduction in pH is the primary factor promoting the dissolution of peptide coacervates.

###

**Figure S65**. Molecular structure of the fluorescent molecules used in the partitioning assays with FMF coacervates and corresponding fluorescence field images (final dye concentration about 0.005 mg mL^-1^). Scale bar = 20 μm.

###

**Figure S66**. Partitioning behaviors of guest molecules in FMF coacervates analyzed by fluorescence spectroscopy. After adding the dye to the coacervates, the mixture was stabilized for 5 min and subjected to low-speed centrifugation to separate the concentrated phase and diluted phases. Then DMSO and Hepes buffer solution were added to the above two phase to prepare solutions with ratio of DMSO compared to Hepes buffer close to 1:1. And their fluorescence intensities were measured to calculate the partitioning efficiency (concentrated phase/diluted phase). (a-h) Fluorescence spectra of the concentrated and diluted phases for various dyes: (a) DTB, (b) Nile red, (c) Rho B, (d) Rho 6G, (e) Rho 110, (f) Calcein, (g) Fluorescein, and (h) FITC-BSA. (i) Partition coefficients of the corresponding guest molecules. 1: DTB, 2: Nile red, 3: Rho B, 4: Rho 6G, 5: Rho 110, 6: Calcein, 7: Fluorescein, 8: FITC-BSA.

###

**Figure S67**. Fluorescent intensity of Nile red (0.05 mg mL^-1^) dispersed in 5 mM Hepes buffer, CH_3_CN, and FMF coacervates (5 mg mL^-1^, 10.9 mM in 5 mM Hepes buffer).

###

**Figure S68**. The distribution of Ph_3_P between dilute and concentrated phases was analyzed by HPLC. (a) The standard curve was constructed first. (b) Ph_3_P (76 mM in DMSO) was then introduced into 1000 μL coacervates (5 mg mL^-1^ FMF, 10.9 mM, 5 mM Hepes buffer, pH~8, finally concentrated of Ph_3_P about 0.228 mM) and subjected to low-speed centrifugation, separating it into dilute phase and concentrated phase. The content ratio of Ph_3_P in concentrated phase to the dilute phase was calculated to be approximately 87:1. Thus the loading efficiency of Ph_3_P inside the coacervates was calculated to be ~98%.

###

**Figure S69**. (a) Assessment of N_3_-coumarin water solubility by HPLC. A mixture of 2 mg N_3_-coumarin in 5 mL of 5 mM Hepes buffer (pH ∼6.5) was shaken for 18 h. After centrifugation, the supernatant was analyzed. Compared to a methanol solution, almost no substrate peak was detected, indicating poor water solubility of N_3_-coumarin through this preliminary experiment. (b) Analysis of N_3_-coumarin distribution between dilute and concentrated phases. N_3_-coumarin (57 mM in DMSO) was added to 1000 μL of coacervates (5 mg mL^-1^ FMF in 5 mM Hepes, 10.9 mM, pH ~8; final N_3_-coumarin concentration ∼0.285 mM), followed by low-speed centrifugation to separate the two phases. The content ratio of N_3_-coumarin in concentrated phase to the dilute phase was calculated to be approximately 97:3.

###

**Figure S70**. Stability of FMF coacervates as microreactor (0.228 mM Ph_3_P, 0.285 mM N_3_-coumarin, 10.9 mM peptide, 5 mM Hepes buffer,100 mM NaCl, pH~8) by incubation for 4 days. During the initial incubation period, the microreactor exhibited highly active fusion and adhesion processes, which were stabilized after prolonged incubation. Scale bar = 10 or 30 μm in microscopy images.

###

**Figure S71**. FRAP traces of FMF coacervates as microreactors (0.228 mM Ph_3_P, 0.285 mM N_3_-coumarin, 10.9 mM peptide, 5 mM Hepes buffer, 100 mM NaCl, pH~8) over time. Data represent mean ± SD for n = 5 representative microscopic images. Error bars (red shaded area) depict the standard deviation (SD) from confocal imaging analysis. Scale bar = 5 μm.

###

###

**Figure S72.** Fluorescence emission from the Staudinger reaction. Upon incubation in the presence of Ph_3_P containing microreactors (0.228 mM Ph_3_P, 10.9 mM FMF peptides, pH ~7, 100 mM NaCl), there is a significant increase in fluorescence emission (λ*_em_* = 450 nm). In contrast, only a very slight increase in emission was observed in the absence of Ph_3_P microreactors (the same condition without Ph_3_P) and no microreactor. Data represent mean ± SD for n = 3 independent samples; Error bars (blue shaded area) represent the standard deviation (n = 3) from microplate reader analysis. And corresponding fluorescence emission spectra of the reaction progress under all the conditions.

###

**Figure S73**. The distribution of N_3_-g-coumarin between dilute and concentrated phases was analyzed by UV-vis spectroscopy. (a) The standard curve was constructed by plotting absorbance against substrate mass of N_3_-g-coumarin, with the substrate dissolved in a 1:1 mixture of MeCN and 5 mM Hepes buffer. (b) N_3_-g-coumarin (29 mM in DMSO) was then introduced into 1000 μL coacervates (5 mg mL^-1^ FMF, 10.9 mM, 5 mM Hepes buffer, pH~8, finally concentrated of N_3_-g-coumarin about 0.29 mM) and subjected to low-speed centrifugation, separating it into dilute phase and concentrated phase. Each fraction was dissolved in a 1:1 mixture of MeCN and 5 mM Hepes buffer for absorbance measurement. The content ratio of N_3_-g-coumarin in concentrated phase to the dilute phase was calculated to be approximately 2.3:1.

###

**Figure S74**. The distribution of g-coumarin between dilute and concentrated phases was analyzed by UV-vis spectroscopy. The standard curve was constructed by plotting absorbance against substrate mass of g-coumarin, with the substrate dissolved in a 1:1 mixture of MeCN and 5 mM Hepes buffer. g-coumarin (59 mM in DMSO) was then introduced into 1000 μL coacervates (5 mg mL^-1^ FMF, 10.9 mM, 5 mM Hepes buffer, pH~8, finally concentrated of g-coumarin about 0.59 mM) and subjected to low-speed centrifugation, separating it into dilute phase and concentrated phase. Each fraction was dissolved in a 1:1 mixture of MeCN and 5 mM Hepes buffer for absorbance measurement. The content ratio of g-coumarin in concentrated phase to the dilute phase was calculated to be approximately 0.0655:1.

###

**Figure S75**. The distribution of β-Gal between dilute and concentrated phases was analyzed by Fluorescence Spectrometer. First, the enzyme was labelled with FITC to produce FITC-labelled enzyme (FITC-β-Gal). Subsequently, FITC-β-Gal (2 mg mL^-1^ in 5 mM Hepes buffer) was loaded into a 300 μL coacervate (5 mg mL^-1^ peptides, 10.9 mM, 5 mM Hepes buffer, finally enzyme concentration about 0.02 mg mL^-1^). Low-speed centrifugation yielded two phases. Then DMSO and Hepes buffer solution were added to the above two phase to prepare solutions with ratio of DMSO compared to Hepes buffer close to 1:1. The fluorescence intensity measurements revealed a content ratio of 0.23 for the concentrated phase relative to the dilute phase. And the loaded efficiency of β-Gal inside coacervates was calculated to be about 18.7%.

###

###

**Figure S76.** Comparison of fluorescence intensity in the presence or absence of coacervates (0.228 mM Ph_3_P, 3 U mL^-1^ β-Gal). There is a significant increase in fluorescence emission (λ*_em_* = 450 nm) when coacervate was present (5 mg mL^-1^ FMF, 10.9 mM, pH~7, 100mM NaCl). Data represent mean ± SD for n = 3 independent samples; Error bars (blue shaded area) represent the standard deviation (n = 3) from microplate reader analysis. And corresponding fluorescence emission spectra of the reaction progress under all the conditions.

###

**Figure S77**. TOP: (a, b) DLS and TEM measurement of BSA@MnO_2_ nanoparticles, which have an average size ~35 nm, and scale bar = 500 nm in the TEM image. (c) BSA@MnO_2_ stabilized complex coacervates. BSA@MnO_2_ particles are located on the surface. (d) FMF coacervates were integrated as organelles within complex coacervates. Scale bar = 10 μm in all microscopy images. Similar results were obtained with 3 samples measured independently.

###

**Figure S78**. Enzyme-controlled sub-coacervates formation. The different compartments were stained with Nile Red (organelles) and FITC BSA (“cytosol”) and the corresponding fluorescence contrast image. Scale bar=10, 7.5 μm in all microscopy images. Similar results were obtained with 3 samples measured independently.

###

###

###

**Figure S79**. 3D cross-sectional images and reconstruction images of artificial cells with enzyme-controlled sub-coacervate formation. Green channel: FITC-BSA; Red channel: Nile Red. Similar results were obtained with 3 samples measured independently. Scale bar=5 μm in all microscopy images.

###

**Figure S80** (a) Schematic representation of cascade reaction happening in multicompartment artificial cell (MACs). (b, c) Comparison of fluorescence intensity in the presence or absence of coacervates (0.228 mM Ph_3_P, 4 U mL^-1^ β-Gal, 0.145 mM N_3_-g-coumarin). There is a significant increase in fluorescence emission (λ*_em_* = 450 nm) when coacervate was present (5 mg mL^−1^ FMF, 10.9 mM, pH~7, 100mM NaCl) in MACs. Data represent mean ± SD for n = 3 independent samples; Error bars (blue shaded area) represent the standard deviation (n = 3) from microplate reader analysis. And corresponding fluorescence emission spectra of the reaction progress after 12 min under all the conditions.

###

**Figure S81** 3D cross-sectional images and reconstruction images of artificial cells when the reaction was completed. Green channel: FITC-BSA; Blue channel: coumarin. Similar results were obtained with 3 samples measured independently. Scale bar=10 μm in all microscopy images.

## 9. Supplementary References:

[1] S. Cao, P. Zhou, G. Shen, T. Ivanov, X. Yan, K. Landfester, L. Caire da Silva, *Nat Commun* **2025**, *16*, 2407.

[2] S. Cao, T. Ivanov, J. Heuer, C. T. J. Ferguson, K. Landfester, L. Caire da Silva, *Nat Commun* **2024**, *15*, 39.

[3] S. S. Matikonda, D. L. Orsi, V. Staudacher, I. A. Jenkins, F. Fiedler, J. Chen, A. B. Gamble, *Chem. Sci.* **2015**, *6*, 1212.

[4] J. Chen, K. Li, J. S. “Lucy” Shon, S. C. Zimmerman, *J. Am. Chem. Soc.* **2020**, *142*, 4565.

[5] M. Abbas, W. P. Lipiński, K. K. Nakashima, W. T. S. Huck, E. Spruijt, *Nat. Chem.* **2021**, *13*, 1046.
